# Rooting the Domain Archaea by Phylogenomic Analysis Supports the Foundation of the New Kingdom Proteoarchaeota

**DOI:** 10.1093/gbe/evu274

**Published:** 2014-12-19

**Authors:** Céline Petitjean, Philippe Deschamps, Purificación López-García, David Moreira

**Affiliations:** Unité d'Ecologie, Systématique et Evolution, CNRS UMR 8079, Université Paris-Sud, Orsay, France

**Keywords:** Archaea, Euryarchaeota, Proteoarchaeota, root, phylogenomics

## Abstract

The first 16S rRNA-based phylogenies of the Archaea showed a deep division between two groups, the kingdoms Euryarchaeota and Crenarchaeota. This bipartite classification has been challenged by the recent discovery of new deeply branching lineages (e.g., Thaumarchaeota, Aigarchaeota, Nanoarchaeota, Korarchaeota, Parvarchaeota, Aenigmarchaeota, Diapherotrites, and Nanohaloarchaeota) which have also been given the same taxonomic status of kingdoms. However, the phylogenetic position of some of these lineages is controversial. In addition, phylogenetic analyses of the Archaea have often been carried out without outgroup sequences, making it difficult to determine if these taxa actually define lineages at the same level as the Euryarchaeota and Crenarchaeota. We have addressed the question of the position of the root of the Archaea by reconstructing rooted archaeal phylogenetic trees using bacterial sequences as outgroup. These trees were based on commonly used conserved protein markers (32 ribosomal proteins) as well as on 38 new markers identified through phylogenomic analysis. We thus gathered a total of 70 conserved markers that we analyzed as a concatenated data set. In contrast with previous analyses, our trees consistently placed the root of the archaeal tree between the Euryarchaeota (including the Nanoarchaeota and other fast-evolving lineages) and the rest of archaeal species, which we propose to class within the new kingdom Proteoarchaeota. This implies the relegation of several groups previously classified as kingdoms (e.g., Crenarchaeota, Thaumarchaeota, Aigarchaeota, and Korarchaeota) to a lower taxonomic rank. In addition to taxonomic implications, this profound reorganization of the archaeal phylogeny has also consequences on our appraisal of the nature of the last archaeal ancestor, which most likely was a complex organism with a gene-rich genome.

## Introduction

Despite the fact that several archaeal species had been isolated at the beginning of the 20th century (Harrison and Kennedy 1922; Barker 1936), the recognition of their nature as members of an independent domain of life had to wait for several decades, when Woese and Fox (1977) published the proposal, based on the analysis of small subunit rRNA sequences (SSU rRNA), that life can be divided into three primary kingdoms: Bacteria, Archaebacteria, and Urkaryotes (Woese and Fox 1977). Later on, to emphasize on the difference between the two major groups of prokaryotes (Bacteria and Archaebacteria), these three kingdoms were reclassified as the domains Bacteria, Archaea, and Eucarya (Woese et al. 1990). Within the Archaea, the first SSU rRNA phylogenies supported the separation of two groups, one containing methanogenic and extreme halophilic species and another formed by thermoacidophilic ones (Fox et al. 1980). This was confirmed by subsequent analyses with a richer taxonomic sampling, leading to the division of the Archaea into two groups with the taxonomic rank of kingdoms: the Crenarchaeota, all hyperthermophilic, and the Euryarchaeota, containing species with a variety of phenotypes (hyperthermophilic, mesophilic, methanogenic, and halophilic; Woese et al. 1990). However, a number of discoveries have challenged this simple bipartite view in recent years.

First, environmental SSU rRNA sequence analyses revealed the existence of archaeal species related to the Crenarchaeota thriving in nonextreme environments such as the open ocean (DeLong 1992; Fuhrman et al. 1992), soils (Jurgens et al. 1997), lakes (Schleper et al. 1997), and both cold and hot terrestrial springs (Barns et al. 1996). Phylogenetic analysis of conserved genes involved in translation and some differences in gene content with the classical hyperthermophilic Crenarchaeota led to propose that these archaeal species may define a new major archaeal group at the same taxonomic level as the Euryarchaeota and the Crenarchaeota (a phylum according to the authors): the Thaumarchaeota (Brochier-Armanet et al. 2008). More recently, a distant relative of the Thaumarchaeota, the species “*Candidatus* Caldiarchaeum subterraneum,” was proposed to define an additional new phylum—Aigarchaeota—based on its distinct gene content (Nunoura et al. 2011). Similar arguments were used to suggest that the species “*Candidatus* Korarchaeum cryptofilum” represented the first member of the new phylum Korarchaeota, very distantly related to the Crenarchaeota (Elkins et al. 2008). An even more divergent case was found with the discovery of the hyperthermophilic *Nanoarchaeum equitans*, a tiny symbiont of the crenarchaeote *Ignicoccus hospitalis*. The phylogenetic analysis of concatenated ribosomal proteins suggested that this species diverged before the separation of the Crenarchaeota and the Euryarchaeota, thus defining a very ancient new archaeal phylum—the Nanoarchaeota (Waters et al. 2003). Although initially classified within the Euryarchaeota, a very deep-branching position has also been suggested for other ultrasmall archaea of the genera “*Candidatus* Micrarchaeum” and “*Candidatus* Parvarchaeum,” using as argument their unusual gene content (Baker et al. 2010). These two genera have recently been proposed to form the new phylum Parvarchaeota by Rinke et al. (2013). These authors retrieved genome sequence information from other divergent archaeal species using single-cell genome approaches and constructed phylogenetic trees based on SSU rRNA and 38 conserved markers (mostly ribosomal proteins and other proteins involved in translation) that supported a very deep-branching position for some of these species, leading to their classification as the two new phyla Aenigmarchaeota and Diapherotrites (Rinke et al. 2013).

Thus, the last decade has seen a multiplication of deep-branching groups within the Archaea and, as a consequence, of the possibilities to place the root of the archaeal tree, namely, the first divergence within this domain of Life. The first analyses placed the root between the Crenarchaeota and the Euryarchaeota (Woese et al. 1990). However, the subsequent inclusion of the new deep-branching groups, in particular the Nanoarchaeota and other ultrasmall species, has challenged this view. For example, the first phylogenetic trees incorporating the Nanoarchaeota, based on rRNA or on ribosomal protein sequences, supported a rooting between *N. equitans* and the rest of archaea (Waters et al. 2003). Other analyses based on similar markers placed *N. equitans* within the Euryarchaeota and the root of the archaeal tree on the branch leading to the Thaumarchaeota (Brochier-Armanet et al. 2008). Concurrently, phylogenetic analyses using heterogeneous sequence evolution models aimed at determining the precise archaeal origin of the eukaryotic nucleocytoplasm have placed the root of the archaeal domain on the *N. equitans* branch or within the Euryarchaeota (Cox et al. 2008; Guy et al. 2014). Another recent analysis included the new phyla Aenigmarchaeota and Diapherotrites (Rinke et al. 2013) and placed the root of the archaeal domain between a large supergroup informally called “DPANN,” which joined all ultrasmall archaea (Diapherotrites, Parvarchaeota, Aenigmarchaeota, Nanoarchaeota, and Nanohaloarchaeota), and the rest of archaea (Rinke et al. 2013).

These different analyses applied different tree reconstruction methods, different markers, different models of sequence evolution, different archaeal sequence samplings, and different outgroup sequences (eukaryotic and/or bacterial), making them difficult to be compared. On the other hand, most archaeal phylogenetic analyses with a wide taxonomic archaeal sampling did not include outgroup sequences, so they only produced unrooted phylogenies (e.g., Brochier-Armanet et al. 2011; Yutin et al. 2012). The results of all these analyses had different implications concerning the nature of the last common ancestor of Archaea (in particular its degree of complexity in terms of gene content) and the early evolution of this domain. Therefore, phylogenetic analyses specifically aimed at rooting the archaeal tree are imperative to solve these questions. For that, a wide representation of all archaeal phyla as well as an adequate sampling of outgroup sequences are necessary. In this study, we have used this approach to reconstruct archaeal phylogenetic trees rooted with a bacterial outgroup. These trees were based on the classical ribosomal protein data set (32 proteins) and on a collection of 38 new conserved proteins identified by phylogenomic analysis. Our phylogenetic analyses supported the rooting between the Euryarchaeota (including the ultrasmall Nanoarchaeota, Parvarchaeota, and Nanohaloarchaeota) and the rest of archaea (Crenarchaeota, Thaumarchaeota, Aigarchaeota, and Korarchaeota). This suggests that the different lineages of ultrasmall archaea evolved from more complex ancestors by a reductive process. Moreover, this deep division into two major groups prompted us to reclassify several major archaeal lineages from their current status of phyla or divisions into the rank of classes to make the whole archaeal taxonomy much more homogeneous.

## Materials and Methods

### Detection of Conserved Proteins Widespread in Archaea and Bacteria

We gathered the protein sequences coded by all publicly available complete genome sequences of archaeal species (using one genome sequence per species to avoid redundancy) as well as those of 117 bacterial species into a local database (for the list of organisms, see supplementary table S1, Supplementary Material online). We then carried out sequence similarity searches against the archaeal sequences in this database using BLAST (Altschul et al. 1997) and all the protein sequences coded in the genomes of *Nitrosopumilus maritimus* and *Cenarchaeum symbiosum* (Thaumarchaeota) and *Caldiarchaeum subterraneum* (Aigarchaeota) as queries. These species were chosen as starting point because Thaumarchaeota have been proposed to have an intermediate gene content between those of the classical Euryarchaeota and Crenarchaeota (Brochier-Armanet et al. 2008), whereas *C. subterraneum* represents a lineage distantly related to the Thaumarchaeota (Nunoura et al. 2011). The BLAST results were filtered to retain only those containing representatives at least four archaeal sequences with an *E*-value threshold of 1e-05. Partial sequences were also removed at this step. The 4,174 proteins thus identified were submitted to preliminary phylogenetic analysis (see later). The phylogenetic trees obtained were checked manually to retain only those where the species belonging to different archaeal classes (e.g., Thermococcales, Halobacteriales, Sulfolobales, and so on) were monophyletic, irrespective of the relative order of emergence of the different classes. Data sets with evidence for horizontal gene transfer (HGT) events (i.e., with archaeal species from different classes intermixed) were also removed, as well as data sets that produced trees with branching patterns suggesting the presence of paralogs (e.g., multiple sequences for some species or species of the same classes branching in different parts of the tree).

Using this conservative approach, 200 data sets were retained, for which we incorporated the respective homologous bacterial sequences and reconstructed new phylogenetic trees, which we used to finally retain 38 proteins for which the respective individual trees did not show evidence for HGT between archaea and bacteria as well as recent HGT events between different bacterial lineage (see the list in supplementary table S2 and figs. S1–S70, Supplementary Material online). To decrease the calculation time of the subsequent phylogenetic analyses, we selected 27 taxonomically diverse bacterial species to be used as outgroup, representing 24 different phyla, and 81 archaeal species representative of the different archaeal phyla.

### Phylogenetic Analyses

Sets of homologous protein sequences were aligned using MAFFT with default parameters (Katoh and Standley 2013). Conserved positions in the alignments were detected using BMGE with default parameters and the BLOSUM62 substitution matrix, which represents a good compromise between too stringent and too relaxed matrices to deal with sequences with a large range of similarity values as in the case of global archaeal phylogenies (Criscuolo and Gribaldo 2010). The trimmed alignments were verified by hand using the program NET of the MUST package (Philippe 1993). Maximum-likelihood (ML) phylogenetic trees were reconstructed upon each individual protein or different concatenated data sets with RaxML v.7.2.4 (Stamatakis 2006) and the PROTGAMMALGF model. Tree robustness was estimated using the Rapid Bootstrapping method implemented in RaxML. Bayesian inference analyses were carried out using MrBayes 3.2.1 (Ronquist et al. 2012) with a mixed model of amino acid sequence evolution and a Gamma distribution with six discrete categories to accommodate for among site rate variation. Four independent chains were run for 2,000,000 generations and trees sampled every 100 trees. To construct a majority rule consensus tree, the first 5,000 trees were eliminated as burn-in.

## Results

### Identification of New Conserved Markers Widely Distributed in Archaea and Bacteria

It has been proposed that the gene content in thaumarchaeotal genomes is to some extent intermediate between those of the two major groups of Archaea (the Euryarchaeota and the Crenarchaeota; Brochier-Armanet et al. 2008, 2012; Spang et al. 2010). For this reason, we used two thaumarchaeotal genomes (*Ce. symbiosum* and *Ni. maritimus*) and one from the closely related Aigarchaeota (*C. subterraneum*) as queries to look for conserved genes widely distributed in Archaea. Using BLAST searches (Altschul et al. 1997) and phylogenetic analyses, we detected 200 proteins with orthologs in most of the available archaeal complete genome sequences. Among them, 81 also had homologues in bacterial species. We reconstructed ML phylogenetic trees for all these proteins to verify that their presence in both Archaea and Bacteria was not due to HGT events between these two domains. We also checked that these proteins were present in at least 24 bacterial phyla to have a sufficiently diverse set of outgroup sequences. This yielded 38 well conserved proteins widely distributed in Archaea and Bacteria (supplementary table S2, Supplementary Material online). We excluded eukaryotes from all our analyses because of the possibility that they might have inherited part of their genes from Archaea during their very early evolution (López-García and Moreira 1999; Embley and Martin 2006; Cox et al. 2008; Gribaldo et al. 2010). If this was actually the case, their use as outgroup may lead to infer an erroneous rooting of the archaeal tree.

In contrast with the limited range of functions of the markers used until now to reconstruct rooted phylogenies of the Archaea (most often ribosomal proteins and a few other proteins also involved in translation), our newly detected proteins participate in large a variety of cellular processes, including the metabolism of amino acids, nucleotides and coenzymes, posttranslational protein modification, and other functions (supplementary table S2, Supplementary Material online). This functional diversity is important because it can contribute to retrieve more homogeneous branch lengths among species as it is unlikely that this entire set of very different cellular processes might have a significantly more accelerated evolutionary rate in some lineages than in the rest. Such very unequal evolutionary rates among lineages represent a major problem for phylogenetic reconstruction and are more likely to occur for sets of proteins involved in a same process. This is exemplified in the case of Archaea by the acceleration of the evolutionary rate of the translation machinery of the species *Methanopyrus kandleri*, which is misplaced in phylogenetic trees based on ribosomal proteins by this reason (Brochier et al. 2004).

In addition to the new 38 protein markers, we also included the classical set of ribosomal proteins (32 proteins, see supplementary table S2, Supplementary Material online) to construct concatenated sequence data sets for the subsequent phylogenetic analyses.

### Rooted Phylogenetic Analyses of Archaea

**Fig. 1.—:**
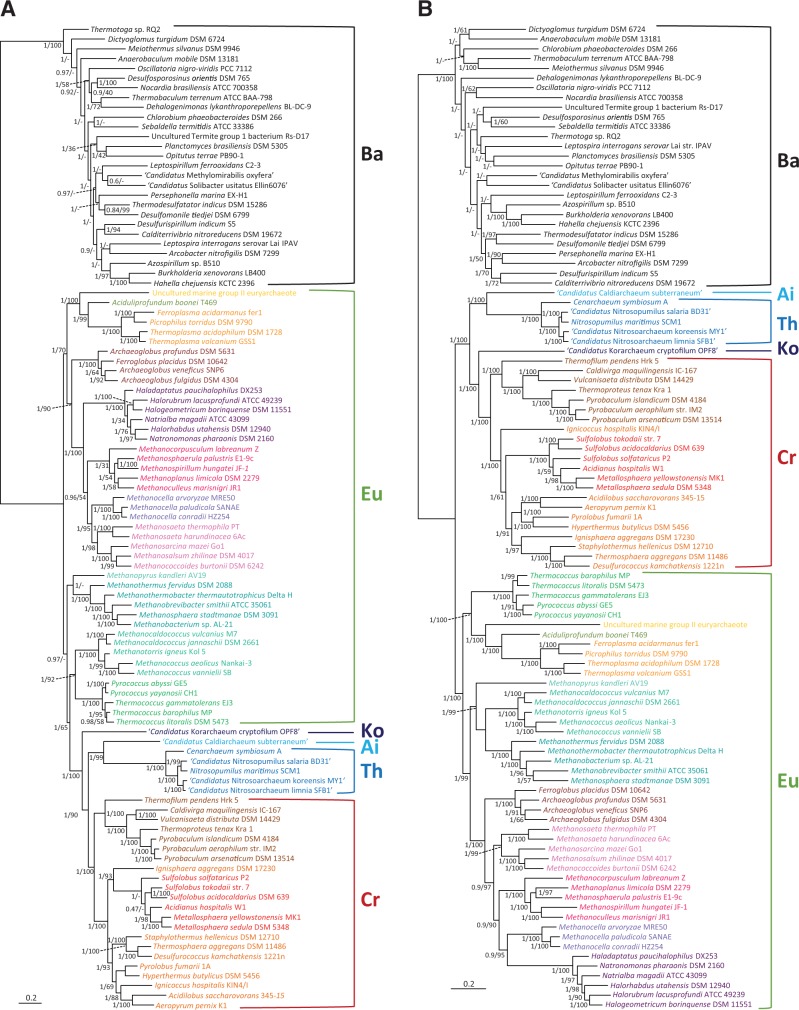
Bayesian phylogenetic trees of Archaea rooted on bacterial sequences. (*A*) Tree based on the concatenation of 32 ribosomal proteins (2,560 sites). (*B*) Tree based on the concatenation of 38 new conserved protein markers (9,540 sites). The groups Bacteria (Ba), Aigarchaeota (Ai), Crenarchaeota (Cr), Korarchaeota (Ko), Thaumarchaeota (Th), and Euryarchaeota (Eu) are indicated. Numbers at branches are Bayesian PPs followed by ML bootstrap values. The scale bar indicates the number of substitutions per position.

Multimarker phylogenetic analyses have revealed that several archaeal lineages have very high evolutionary rates, producing very long branches in phylogenetic trees which can lead to long-branch attraction artefacts (LBA). This is notably the case for the ultrasmall archaea of the genera *Nanoarchaeum*, *Micrarchaeum*, *Parvarchaeum,* and the Nanohaloarchaea (e.g., Brochier-Armanet et al. 2011). Therefore, we carried out a first set of phylogenetic analyses excluding all these fast-evolving organisms. The first data set that we analyzed contained the classical set of ribosomal proteins that have traditionally been used to reconstruct rooted and unrooted archaeal phylogenies (e.g., Cox et al. 2008; Elkins et al. 2008; Brochier-Armanet et al. 2011; Guy and Ettema 2011). We updated the sequence data set for those markers (32 proteins, 2,560 amino acid sites) by incorporating representative species from all major archaeal lineages with the exception of the fast-evolving ones, as mentioned earlier. The resulting phylogenetic tree was rooted with the bacterial sequences and placed the root of the Archaea within the Euryarchaeota (fig. 1*A*). In fact, different euryarchaeotal lineages emerged as paraphyletic branches at the base of the tree, with strong statistical support in Bayesian trees (posterior probabilities [PPs] = 0.97–1) but moderate bootstrap support in ML trees (BP < 70%). This result was in agreement with a similar tree published by Cox et al. (2008). The apical part of our tree was occupied by a strongly supported group (PP = 1, BP = 100%) containing the Thaumarchaeota, Aigarchaeota, Crenarchaeota, and Korarchaeota (a group tentatively defined as the “TACK” superphylum [Guy and Ettema 2011]).

**Fig. 2.—:**
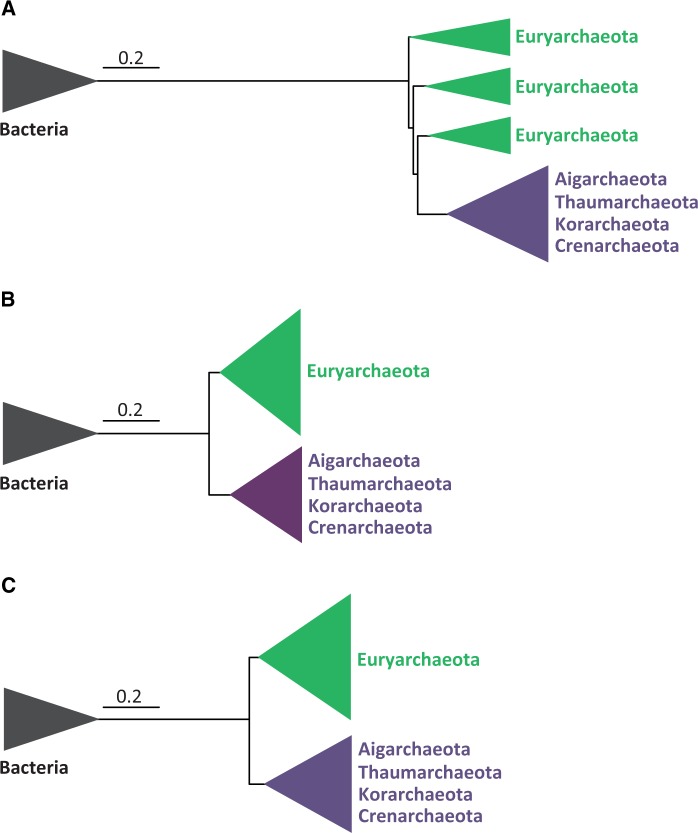
Schematic Bayesian phylogenetic trees of Archaea rooted on bacterial sequences. All trees are shown at the same scale to compare the distance between Archaea and Bacteria. (*A*) Tree based on 32 ribosomal proteins (2,560 sites). (*B*) Tree based on 38 new conserved proteins (9,540 sites). (*C*) Tree based on the complete data set of 70 proteins (10,963 sites). The scale bar indicates the number of substitutions per position.

We then carried out a phylogenetic analysis of a concatenation of our new markers (38 markers, 6,890 amino acid sites). In sharp contrast with the previous tree based on translation-related proteins, the Bayesian phylogenetic tree based on this new data set was rooted between the whole clade of the Euryarchaeota and a group containing the rest of archaeal species, namely the TACK supergroup (fig. 1*B*). This rooting was robustly supported (PP = 1, BP = 99–100%). Moreover, the tree also strongly supported (PP = 1, BP > 90%) the monophyly of most of the archaeal classes commonly accepted to be monophyletic based on phylogenomic and gene-content analyses (Brochier-Armanet et al. 2011, Wolf et al. 2012). This suggested that this data set contained exploitable phylogenetic signal. One important difference between the phylogenetic tree based on these proteins and the one based on the ribosomal proteins concerned the length of the branch uniting the Archaea and the Bacteria. Unexpectedly, despite the presumed high conservation of the ribosomal structure and activity in all living beings, this branch was much longer in the ribosomal proteins tree than in the tree based on the new markers (a 2.6× difference, fig. 2), indicating that the new markers have kept a higher sequence similarity between these two life domains than the ribosomal proteins.

**Fig. 3.—:**
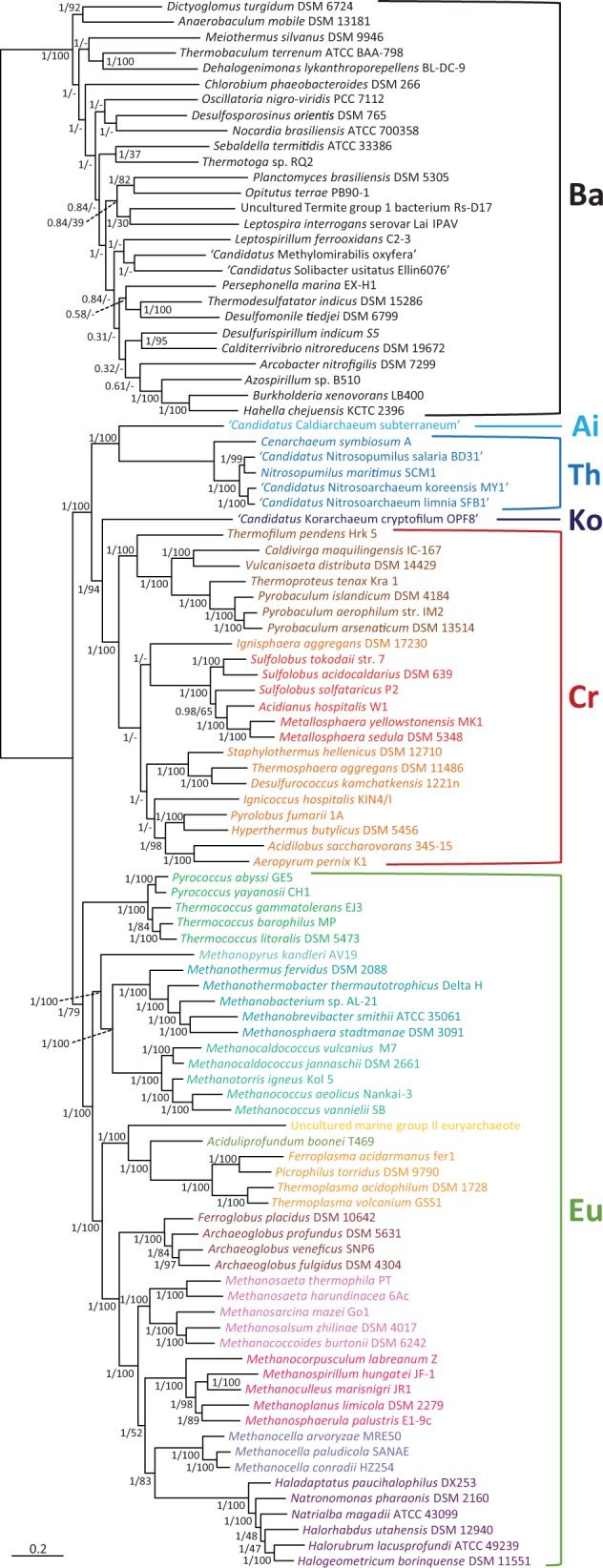
Bayesian phylogenetic tree of Archaea rooted on bacterial sequences based on the concatenation of 32 ribosomal proteins and 38 new conserved proteins (10,963 sites). The groups Bacteria (Ba), Aigarchaeota (Ai), Crenarchaeota (Cr), Korarchaeota (Ko), Thaumarchaeota (Th), and Euryarchaeota (Eu) are indicated. Numbers at branches are PPs followed by ML bootstrap values. The scale bar indicates the number of substitutions per position.

Finally, we analyzed a combined data set containing all the previous markers (translation-related proteins plus our new markers, for a total of 70 markers and 9,540 amino acid sites). The resulting tree (fig. 3) was very similar to the one obtained from the analysis of the concatenation of the new markers. In fact, the root was placed between the Euryarchaeota and the rest of archaeal species with strong support, especially by the Bayesian analysis (PP = 1 for all deep nodes in the tree) and moderately by the ML analysis (BP = 79% for the monophyly of the Euryarchaeota and 100% for the TACK group). The distance between the Bacteria and the Archaea was only slightly longer than for the tree based on ribosomal proteins (1.3×, fig. 2) The internal phylogeny of the different archaeal lineages was also well supported in the Bayesian tree, even for several difficult-to-resolve relationships. For example, we retrieved full support (PP = 1, BP = 100%) for the monophyly of the so-called “Methanogen Class I” containing the Methanopyrales, Methanobacteriales, and Methanococcales, a relationship that was not supported by the ribosomal proteins, which placed the Methanococcales as sister group of the Thermococcales (fig. 1*A*). Another highly supported (PP = 1, BP = 99%) interesting result was the position of the Halobacteria as a derived group within the class Methanomicrobia (also known as “Methanogen Class II”).

### The Position of Long-Branching Ultrasmall Archaea

As mentioned earlier, we excluded from our initial phylogenetic analyses a number of taxa characterized by their very long branches. These included the Nanoarchaeota, Parvarchaeota, and Nanohaloarchaea. An important problem concerning these species was the very large amount of missing data in our concatenated alignments (>50% in the complete data set of 70 proteins), in agreement with their very small genome sizes (especially for *N. equitans*, with only 540 protein-coding genes, and *Micrarchaeum* and *Parvarchaeum*, with approximately 1,000 protein-coding genes). In the case of the new ultrasmall archaeal lineages Aenigmarchaeota and Diapherotrites, this problem was even more pronounced due to the incomplete genome sequence coverage for these species. This is concomitant to the single-cell genome approach used to study them, which requires a step of whole genome amplification that is known to be biased and to lead to uneven genome coverage (Worden et al. 2011). Thus, we had 72.3% and 71% of missing data for these two lineages in our data set. In addition, phylogenetic trees reconstructed with 16S rRNA sequences and ribosomal conserved proteins showed that most often they have very long branches, as it also the case for Nanoarchaeota, Parvarchaeota, and Nanohaloarchaea (supplementary figs. S71–S77, Supplementary Material online). Those long branches suggested an accelerated evolutionary rate that makes these species very prone to potential LBA artifacts (Roure et al. 2013). Because of their accelerated evolutionary rate and the large amount of missing data, we decided to remove the Aenigmarchaeota and Diapherotrites from our analyses to keep only the other ultrasmall archaeal species with bona fide complete genome sequences available.

**Fig. 4.—:**
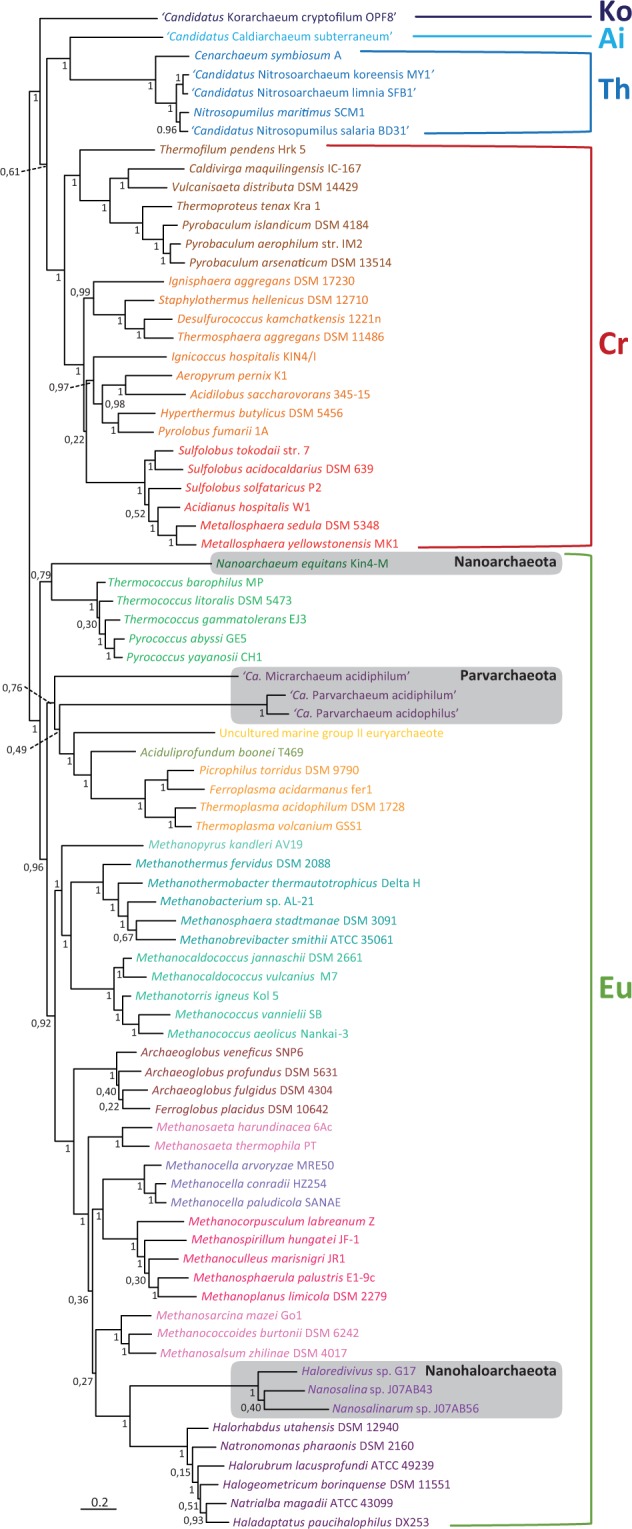
Unrooted Bayesian phylogenetic tree of Archaea including ultrasmall archaeal taxa (Nanoarchaeota, Parvarchaeota, and Nanohaloarchaeota). The tree is based on the concatenation of 32 ribosomal proteins and 38 new conserved proteins (10,963 sites). The groups Bacteria (Ba), Aigarchaeota (Ai), Crenarchaeota (Cr), Korarchaeota (Ko), Thaumarchaeota (Th), and Euryarchaeota (Eu) are indicated Numbers at branches are PPs. The scale bar indicates the number of substitutions per position.

LBA problems can be exacerbated by the inclusion of distant outgroup sequences, leading to a basal emergence of the long-branching taxa attracted by the outgroup (Philippe and Laurent 1998; Brinkmann et al. 2005). In fact, although poorly supported, the ML tree based on our complete set of markers and rooted using the bacterial sequences as outgroup showed a basal emergence of the long-branching ultrasmall archaeal taxa within a monophyletic group (BP = 62%, supplementary fig. S78, Supplementary Material online). This would support that the root of the archaeal tree is located between these species and the rest of archaea, as recently proposed by Rinke et al. (2013). However, previous analyses have provided strong support for alternative placements of these species, in particular for *Nanoarchaeum* as sister group of the Thermococcales (Brochier et al. 2005) and the Nanohaloarchaea as sister group of the Halobacteria (Narasingarao et al. 2012). We thus tested different ways to reduce the potential LBA artefact responsible of the basal emergence of the long-branching archaea. First, we carried out an unrooted phylogenetic analysis excluding the bacterial sequences. In the resulting ML tree all the ultrasmall archaea branched again as a strongly supported monophyletic group placed between the Crenarchaeota and the Euryarchaeota (supplementary fig. S79, Supplementary Material online). This suggested that the removal of the distant bacterial outgroup sequences was not enough to alleviate the possible LBA artefact responsible for their basal emergence. We thus reanalyzed the same data set using Bayesian inference with a mixed model of amino acid sequence evolution, which can better accommodate the different patterns of evolution shown by the different markers than simpler methods and models (Pagel and Meade 2004), such as the approximate ML reconstruction with the very simple site-homogeneous model Jones, Taylor, and Thorton (JTT) used by Rinke et al. (2013). Inspection of our Bayesian run results revealed that, in fact, several sequence evolution models were explored, especially in the initial phase of the run where the different Markov Chain Monte Carlo parameters fluctuated. Our Bayesian tree showed a topology very different from the Rinke et al.’s basal emergence of ultrasmall lineages. In our tree, the four groups of long-branching ultrasmall archaea emerged well nested at different locations within the Euryarchaeota (fig. 4). Nanoarchaeota branched as sister group of the Thermococcales, the genera *Micrarchaeum* and *Parvarchaeum* as sisters of the Thermoplasmata, and the Nanohaloarchaea as sisters of the Halobacteria. These results supported that these taxa can be considered as bona fide euryarchaeotal species and that, hence, the root of the archaeal tree does not lie between them and the other archaea.

## Discussion

### The Root of the Archaeal Tree

Since the initial proposal by Woese et al. (1990) that the deepest division in the archaeal domain was between the two kingdoms Crenarchaeota and Euryarchaeota, many other alternatives have been advanced, most of them linked to the discovery of new deeply branching nanosized archaeal lineages. As summarized in the Background section, this has been the case especially for the hyperthermophilic *N. equitans* (Waters et al. 2003; Cox et al. 2008), the Thaumarchaeota (Brochier-Armanet et al. 2008), the acidophilic genera *Candidatus* “Micrarchaeum” and “Parvarchaeum” (Baker et al. 2010), and the Aenigmarchaeota and Diapherotrites (Rinke et al. 2013). Di Giulio (2006) has fervently argued for the rooting on the Nanoarchaeota and even proposed that this group should be considered as a “living fossil.” His conclusion was based not only on phylogenetic analyses of rRNA sequences (Branciamore et al. 2008) but also on the unusual discovery of some genes split in two fragments in the genome of *N. equitans* (Randau et al. 2005), which was interpreted as an ancestral character according to the “introns early” hypothesis (Di Giulio 2008). However, several findings have undermined this proposal. Split genes have also been found in another small archaeon, *Micrarchaeum acidiphilum*, which is phylogenetically not closely related to *N. equitans* (Baker et al. 2010). In addition, phylogenetic analyses based on individual conserved proteins and on concatenated translation-related proteins strongly support that Nanoarchaeota are not basal but sisters to the Thermococcales (Brochier et al. 2005; Brochier-Armanet et al. 2011). Likewise, a very deep-branching position for the genera *Micrarchaeum* and *Parvarchaeum* based on their gene content with both typical euryarchaeotal and crenarchaeotal genes (Baker et al. 2010) has not been validated by phylogenetic analysis of translation-related proteins, which placed them nested within the Euryarchaeota (Brochier-Armanet et al. 2011). Finally, the halophilic Nanohaloarchaea have been shown to robustly branch close to the Halobacteria (Narasingarao et al. 2012). Our Bayesian analyses with the complete set of markers are in agreement with these results, showing all the long-branching archaea well nested within the Euryarchaeota, with the Thermococcales + Nanoarchaeota as the first group to diverge (fig. 4). Interestingly, when bacterial sequences were added to root this tree, all those long-branching archaea emerged together at the base of the archaeal tree, strongly suggesting a LBA artefact. In a different analysis based on the concatenation of 67 conserved proteins and including also eukaryotic sequences, Guy et al. (2014) made a comparable observation. Although they retrieved the root of the archaeal tree within the Euryarchaeota, in agreement with previous studies with a similar taxonomic sampling (Cox et al. 2008), the three ultrasmall archaeal genera included in the analysis (*Nanoarchaeum*, *Micrarchaeum*, and *Parvarchaeum*) formed a well-supported monophyletic group that branched between the paraphyletic Euryarchaeota and the TACK supergroup.

The possibility that the root of the archaeal tree lies on the ultrasmall archaeal lineages gained support recently after the characterization of new genome sequences obtained by single-cell-based approaches that led to the discovery of two new lineages, the Aenigmarchaeota and the Diapherotrites (Rinke et al. 2013). As mentioned in the Introduction, a phylogenetic analysis based on 38 markers, most of them ribosomal proteins, retrieved a monophyletic group, informally dubbed DPANN, containing these two lineages and the other ultrasmall archaeal groups (Nanoarchaeota, Parvarchaeota, and Nanohaloarchaeota, see supplementary fig. S11, Supplementary Material online, in Rinke et al. [2013]). Interestingly, the root of the Archaea was located between this group and the rest of archaeal lineages. This analysis was done with an approximate ML method (Price et al. 2010) and a simple sequence evolution model (the JTT one; Jones et al. 1992). Recently, Williams and Embley (2014) have investigated the phylogeny of the ultrasmall archaea. On the one hand, they carried out a Bayesian analysis based on a set of 29 conserved universal proteins. This analysis placed the DPANN lineages as sister to the rest of archaea although with very poor support (PP = 0.54). On the other hand, they reanalyzed Rinke et al.’s sequence data sets and unveiled a number of problems. The most important one was the detection of sequences of mitochondrial and plastid origin in the eukaryotic data. When these markers were replaced by the bona fide eukaryotic homologs and the corrected data set analyzed with a more realistic substitution model (CAT + general time reversible), the DPANN group branched within the Euryarchaeota, which emerged as a paraphyletic group.

All these results tend to support that the root of the archaeal tree does not lie on the fast-evolving ultrasmall archaea. Thus, the atypical characteristics found in these archaea are most likely derived characters rather than ancestral features. One example concerns the split genes of *Nanoarchaeum* and *Micrarchaeum*, which have likely evolved by convergence in these two lineages. This can also be the case for the fused primase genes found in *Nanoarchaeum*, *Parvarchaeum,* and the nanohaloarchaeote *Nanosalinarum* (but not in the closely related *Nanosalina*), although it has also been proposed that the distribution of these genes in the ultrasmall archaeal lineages may be the result of HGT events (Raymann et al. 2014). Massive genome size reduction is another likely convergent trait in these organisms, all of them having gene numbers smaller than those of their respective closest relatives. It has been accompanied by a large acceleration of evolutionary rate, as attested by the long branches exhibited by all these species. It may be possible that anomalous characters, such as the split genes, are side products of that evolutionary acceleration and genome reduction. In fact, convergent acquisition of exceptional features has already been noticed in fast-evolving highly reduced genomes. One outstanding example in eukaryotic species is the migration of the rRNA genes to subtelomeric regions in the chromosomes of the highly reduced nucleomorph genomes of two unrelated lineages, the cryptophytes and chlorarachniophytes (Moore and Archibald 2009).

One remarkable result of our analyses was the very long distance between Archaea and Bacteria observed in the tree based on ribosomal proteins (fig. 1*A*), more than twice longer than the distance calculated with the new 38 proteins or the complete 70 proteins data set (fig. 2). This was unexpected because ribosomes are considered to be among the most conserved molecular machineries in bacteria, archaea, and eukaryotes. However, despite this overall conservation, archaeal and bacterial ribosomes exhibit important differences in the set of proteins that build them. Whereas more than 30 proteins are shared by the three domains of life, 33 are shared between archaea and eukaryotes to the exclusion of bacteria, and 23 are bacteria-specific (Lecompte et al. 2002; Yutin et al. 2012). Ribosomes are integrated macromolecular structures were rRNAs and proteins are tightly connected displaying a large number of highly specific interactions that are essential for ribosomal function. Consequently, changing some proteins would have a global effect on the whole ribosome structure, which is the reason why it is commonly believed that rRNAs and ribosomal proteins are less affected by HGT than genes coding for proteins not involved in macromolecular assemblages (Jain et al. 1999; Brochier et al. 2000). However, as mentioned earlier, archaeal and bacterial ribosomes have significantly different protein compositions despite a shared core. It is thus possible that the acquisition of those different protein sets may have affected the evolutionary rate of the shared proteins, which have had to adapt to new interactions with the nonshared proteins. This acceleration of evolutionary rate would have occurred in the stem branches of the domains Bacteria and Archaea, namely after their separation but before the diversification of the lineages now present in these two domains. This would explain the very long distance between Bacteria and Archaea in the ribosomal protein tree (fig. 2). In addition, most ribosomal proteins are small, so their concatenation only yielded 2,560 sites, whereas the new proteins produced a much longer concatenation of 6,890 sites despite a comparable number of proteins (32 versus 38, respectively). The very long branch leading to the outgroup and the small number of sites may explain the anomalous result observed in ribosomal proteins trees, in particular the paraphyly of the Euryarchaeota at the base of the Archaea (fig. 1*A*). This leads to asymmetrical tree topologies, which are known to often reflect tree reconstruction artifacts (Philippe and Adoutte 1998; Moreira et al. 1999). Thus, despite being commonly used (e.g., Cox et al. 2008), ribosomal proteins alone are probably not the most appropriate markers to reconstruct very ancient evolutionary events such as the root of the archaeal tree or the relationships among the three domains of Life.

### A Bipartite Division of the Domain Archaea and Proposal for the New Archaeal Kingdom Proteoarchaeota

Originally, the domain Archaea was divided into two major groups based on the analysis of 16S rRNA sequences: the Euryarchaeota and the Crenarchaeota (Woese et al. 1990). These two groups were given the taxonomic rank of Kingdom as the one immediately below that of Domain and to insist on their clear phylogenetic distinctiveness (Woese et al. 1990). However, since the publication of this proposal, several new archaeal lineages have been discovered and some of them have been named using the suffix “archaeota” to equal their rank to that of the Euryarchaeota and Crenarchaeota. This is the case of the Nanoarchaeota (Waters et al. 2003), Thaumarchaeota (Brochier-Armanet et al. 2008), Korarchaeota (Elkins et al. 2008), Aigarchaeota (Nunoura et al. 2011), Parvarchaeota, and Aenigmarchaeota (Rinke et al. 2013). The lack of accurate criteria to establish a rank for those different lineages has fostered discussion on different taxonomic aspects, such as the weight that has to be given to molecular phylogeny on the definition of new major taxa (Garrity and Oren 2012; Gribaldo and Brochier-Armanet 2012).

Our phylogenetic analyses excluding the fast-evolving taxa (*Nanoarchaeum*, Parvarchaeota, Nanohaloarchaea, Aenigmarchaeota, and Diapherotrites) strongly supported that the root of the archaeal tree lies between the Euryarchaeota and the rest of archaeal lineages. This, together with the observation that the long-branching archaea can be considered as bona fide Euryarchaeota (they branch within this archaeal group when LBA problems are minimized by using robust methods and the taxa with the smallest amount of missing data), advocates for a major division of the domain Archaea into two major groups between which the root is located: the Euryarchaeota and the so-called TACK supergroup. In addition to these phylogenetic considerations, these two groups have similar levels of ecological and evolutionary diversity. As their name evokes (*eurus* meaning wide), Euryarchaeota have for long been known to exhibit a variety of metabolic capacities and to occupy a broad range of habitats (Woese et al. 1990). This is also the case for the TACK supergroup (Guy and Ettema 2011), thriving in high-temperature environments (Crenarchaeota, Aigarchaeota, and Korarchaeota) but also in lower-temperature ones (many Thaumarchaeota) and relying on a large variety of metabolisms. On the other hand, the evolutionary divergence among distant TACK members is very similar to that among distant Euryarchaeota. For example, the average 16S rRNA sequence identity between Thaumarchaeota and Desulfurococcales (Crenarchaeota) is of approximately 75%, identical to that between two distant euryarchaeotal lineages, Thermococcales and Halobacteria. The phylogenetic depth, in terms of sequence divergence, for our set of protein markers was also similar for the Euryarchaeota and the TACK group (∼65% amino acid sequence similarity for comparisons of the same taxa as earlier).

The separation of the Euryarchaeota and the TACK group represents the primary split among the known archaeal species and these two groups have comparable ecological and phylogenetic diversities. Thus, it would be logical to give the TACK group the same taxonomic level as the Euryarchaeota, namely a kingdom rank to keep the initial Woesian nomenclature, or a superphylum rank as commonly used for Bacteria. This would require providing a formal name to the TACK group. We propose to call this new kingdom or superphylum Proteoarchaeota, making reference to the Greek god of the sea Proteus, able to display many different forms. The same prefix Proteo- was used in the name Proteobacteria also to point to the vast phenotypic diversity found in this bacterial group (Stackebrandt et al. 1988). To avoid confusion and make the archaeal taxonomy more homogeneous, the erection of the kingdom Proteoarchaeota would entail the relegation in rank of several archaeal lineages that were given a kingdom (or superphylum) level. As mentioned earlier, this concerns the Nanoarchaeota, Thaumarchaeota, Aigarchaeota, Korarchaeota, Aenigmarchaeota, and Parvarchaeota. They should be reclassified as classes as it is the case for the different lineages that compose the Euryarchaeota. Therefore, we propose to apply them the new names Nanoarchaea, Thaumarchaea, Aigarchaea, Korarchaea, Aenigmarchaea, and Parvarchaea with their respective orders Nanoarchaeales, Thaumarchaeales, Aigarchaeales, Korarchaeales, Aenigmarchaeales, and Parvarchaeales (table 1). Likewise, the former kingdom Crenarchaeota should be renamed as a class (Crenarchaea, which would be synonym of Thermoprotei). We consider that this amended scheme is the one that requires the smallest number of taxonomic changes (thus respecting the principle of taxonomic stability) and, at the same time, would be much more consistent for the whole archaeal domain than the current mix of different ranks (kingdoms, classes and orders) to refer to lineages that, actually, have similar phylogenetic breath and can be joined into only two major groups.

**Table 1:** Revised Classification of Archaea into Two Major Superphyla or Kingdoms

Superphylum (Kingdom)	Class	Order
Euryarchaeota	Archaeoglobi	Archaeoglobales
	Methanobacteria	Methanobacteriales
	Methanococci	Methanococcales
	Methanomicrobia	Methanocellales
		Methanomicrobiales
		Methanosarcinales
		Halobacteriales
	Methanopyri	Methanopyrales
	Nanoarchaea	Nanoarchaeales
	Parvarchaea	Parvarchaeales
	Thermococci	Thermococcales
	Thermoplasmata	Thermoplasmatales
Proteoarchaeota	Aigarchaea	Aigarchaeales
	Crenarchaea^a^	Acidilobales
		Desulfurococcales
		Fervidicoccales
		Sulfolobales
		Thermoproteales
	Korarchaea	Korarchaeales
	Thaumarchaea	Cenarchaeales
		Nitrosopumilales
		Nitrososphaerales

^a^The current class Thermoprotei would be synonym of Crenarchaea.

## Conclusions

In addition to the classical phylogenetic markers commonly used until now, most of them ribosomal proteins, our phylogenomic survey has allowed identifying 38 additional conserved proteins that can be used to reconstruct phylogenetic trees of the archaea rooted on bacterial homologues. The phylogenetic analyses of the complete set of all those markers (32 ribosomal and 38 new ones) converge to support a deep division of the domain Archaea into two major lineages. One corresponds to the kingdom Euryarchaeota, already defined two decades ago (Woese et al. 1990) and the second to a miscellaneous collection of lineages that has been tentatively grouped under the informal denomination of TACK supergroup (Guy and Ettema 2011). This second group is clearly monophyletic in our analyses and has a level of phylogenetic diversity comparable to the one exhibited by the Euryarchaeota. In addition, the lineages composing it show a large panel of ecological adaptations. These points are not reflected by the taxonomic classification of the Archaea currently used, which gives similar status to groups as different in phylogenetic diversity and depth as the extremely wide Euryarchaeota and the much more reduced Nanoarchaeota, Korarchaeota or Aigarchaeota. We think that the best alternative would be to give the two major archaeal lineages the same taxonomic rank. For that, the most parsimonious solution is keeping the kingdom/superphylum rank already given to the Euryarchaeota and to erect a new kingdom/superphylum to contain the TACK lineages. We propose to call these new kingdom Proteoarchaeota to highlight its high ecological and phylogenetic diversity.

The rooting of the archaeal tree on the ultrasmall archaeal branches, either the Nanoarchaeota (Di Giulio 2006) of the DPANN supergroup (Rinke et al. 2013), supported the idea that the last common ancestor of Archaea was a simple organism with a small genome. However, the rooting between the Euryarchaeota and the Proteoarchaeota, two groups containing both species with both large and small genomes, opens the possibility that the ancestor had a complex, gene-rich genome. In fact, a recent analysis aimed at reconstructing the ancestral gene content in archaea by an ML approach agrees with this possibility, as it supports that the last common ancestor of Archaea was a complex organism with at least 2,500 protein-coding genes (Wolf et al. 2012). The results of this type of analysis are highly dependent on the taxonomic sampling (e.g., Wolf et al. only considered one DPANN lineage, the Nanoarchaeota), the tree topology, and the position of the root. Our Euryarchaeota versus Proteoarchaeota rooting agrees with the view of a last common archaeal ancestor with a gene content most likely larger than that of ultrasmall archaea. It has been recently shown that most major archaeal lineages have increased their gene repertoires by massive HGT acquisition from bacterial donors (Nelson-Sathi et al. 2012, 2014; Deschamps et al. 2014). Thus, whereas HGT-mediated genome size increase appears to have been a common evolutionary trend in many archaea, massive gene loss has probably occurred in the different groups of ultrasmall species that emerged within the Euryarchaeota. This phenomenon has most likely occurred independently in these lineages, so that their current small genomes would be the result of convergence and not an ancestral character.

## Supplementary Material

Supplementary tables S1 and S2 and figures S1–S77 are available at *Genome Biology and Evolution* online (http://www.gbe.oxfordjournals.org/).

Supplementary Data
