# Supplementary material for: Rooting the Domain Archaea by Phylogenomic Analysis Supports the Foundation of the New Kingdom Proteoarchaeota
Source: Genome Biol Evol. 2014 Dec 19;7(1):191–204. doi: 10.1093/gbe/evu274 (PMC4316627; doi:10.1093/gbe/evu274)
Supplement: Supplementary Data [file supp_7_1_191__index.html]

Rooting the Domain Archaea by Phylogenomic Analysis Supports the Foundation of the New Kingdom Proteoarchaeota — Rooting the Domain Archaea by Phylogenomic Analysis Supports the Foundation of the New Kingdom Proteoarchaeota — Supplementary Data 

# Rooting the Domain Archaea by Phylogenomic Analysis Supports the Foundation of the New Kingdom Proteoarchaeota

## Supplementary Data

files

**Files in this Data Supplement:**

- Supplementary Data - pdf file
