# Supplementary material for: Rooting the Domain Archaea by Phylogenomic Analysis Supports the Foundation of the New Kingdom Proteoarchaeota
Source: Genome Biol Evol. 2014 Dec 19;7(1):191–204. doi: 10.1093/gbe/evu274 (PMC4316627; doi:10.1093/gbe/evu274)
Supplement: Supplementary Data [file supp_evu274_Petitjean-etal-Supplementary-Material_revised.pdf]

**Supplementary table S1.** List of archaeal and bacterial complete genome sequences used in this work.

| | TaxID | Species | | Phylum | Class |
|---|---|---|---|---|---|
| | | * : species retained for the construction of the final datasets (108 species) | | | |
| 1 | 311458 | *Candidatus* Caldiarchaeum subterraneum | * | Aigarchaeota | Unclassified |
| 2 | 414004 | *Cenarchaeum symbiosum A* | * | Thaumarchaeota | Cenarchaeales |
| 3 | 436308 | *Nitrosopumilus maritimus SCM1* | * | Thaumarchaeota | Nitrosopumilales |
| 4 | 886738 | *Candidatus* Nitrosoarchaeum limnia SFB1 | * | Thaumarchaeota | Nitrosopumilales |
| 5 | 1001994 | *Candidatus* Nitrosoarchaeum koreensis MY1 | * | Thaumarchaeota | Nitrosopumilales |
| 6 | 859350 | *Candidatus* Nitrosopumilus salaria BD31 | * | Thaumarchaeota | Nitrosopumilales |
| 7 | 797209 | *Haladaptatus paucihalophilus DX253* | * | Euryarchaeota | Halobacteriales |
| 8 | 795797 | *Halalkalicoccus jeotgali B3* | | Euryarchaeota | Halobacteriales |
| 9 | 634497 | *Haloarcula hispanica ATCC 33960* | | Euryarchaeota | Halobacteriales |
| 10 | 272569 | *Haloarcula marismortui ATCC 43049* | | Euryarchaeota | Halobacteriales |
| 11 | 478009 | *Halobacterium salinarum R1* | | Euryarchaeota | Halobacteriales |
| 12 | 309800 | *Haloferax volcanii DS2* | | Euryarchaeota | Halobacteriales |
| 13 | 469382 | *Halogeometricum borinquense DSM 11551* | * | Euryarchaeota | Halobacteriales |
| 14 | 485914 | *Halomicrobium mukohataei DSM 12286* | | Euryarchaeota | Halobacteriales |
| 15 | 756883 | *halophilic archaeon DL31* | | Euryarchaeota | Halobacteriales |
| 16 | 797210 | *Halopiger xanaduensis SH-6* | | Euryarchaeota | Halobacteriales |
| 17 | 362976 | *Haloquadratum walsbyi DSM 16790* | | Euryarchaeota | Halobacteriales |
| 18 | 1033806 | *Halorhabdus tiamatea SARL4B* | | Euryarchaeota | Halobacteriales |
| 19 | 519442 | *Halorhabdus utahensis DSM 12940* | * | Euryarchaeota | Halobacteriales |
| 20 | 416348 | *Halorubrum lacusprofundi ATCC 49239* | * | Euryarchaeota | Halobacteriales |
| 21 | 543526 | *Haloterrigena turkmenica DSM 5511* | | Euryarchaeota | Halobacteriales |
| 22 | 547559 | *Natrialba magadii ATCC 43099* | * | Euryarchaeota | Halobacteriales |
| 23 | 797303 | *Natrinema pellirubrum DSM 15624* | | Euryarchaeota | Halobacteriales |
| 24 | 797304 | *Natronobacterium gregoryi SP2* | | Euryarchaeota | Halobacteriales |
| 25 | 348780 | *Natronomonas pharaonis DSM 2160* | * | Euryarchaeota | Halobacteriales |
| 26 | 1072681 | *Candidatus* Haloredivivus sp. G17 | * | Euryarchaeota | Nanohaloarchaea |
| 27 | 889948 | *Candidatus* Nanosalina sp. J07AB43 | * | Euryarchaeota | Nanohaloarchaea |
| 28 | 889962 | *Candidatus* Nanosalinarum sp. J07AB56 | * | Euryarchaeota | Nanohaloarchaea |
| 29 | 410358 | *Methanocorpusculum labreanum Z* | * | Euryarchaeota | Methanomicrobiales |
| 30 | 368407 | *Methanoculleus marisnigri JR1* | * | Euryarchaeota | Methanomicrobiales |
| 31 | 882090 | *Methanolinea tarda NOBI-1* | | Euryarchaeota | Methanomicrobiales |
| 32 | 937775 | *Methanoplanus limicola DSM 2279* | * | Euryarchaeota | Methanomicrobiales |
| 33 | 679926 | *Methanoplanus petrolearius DSM 11571* | | Euryarchaeota | Methanomicrobiales |
| 34 | 456442 | *Methanoregula boonei 6A8* | | Euryarchaeota | Methanomicrobiales |
| 35 | 521011 | *Methanosphaerula palustris E1-9c* | * | Euryarchaeota | Methanomicrobiales |
| 36 | 323259 | *Methanospirillum hungatei JF-1* | * | Euryarchaeota | Methanomicrobiales |

| | | | | |
|---|---|---|---|---|
| 37 | 259564 | *Methanococcoides burtonii DSM 6242* | * Euryarchaeota | Methanosarcinales |
| 38 | 644295 | *Methanohalobium evestigatum Z-7303* | Euryarchaeota | Methanosarcinales |
| 39 | 547558 | *Methanohalophilus mahii DSM 5219* | Euryarchaeota | Methanosarcinales |
| 40 | 990316 | *Methanosaeta concilii GP6* | Euryarchaeota | Methanosarcinales |
| 41 | 1110509 | *Methanosaeta harundinacea 6Ac* | * Euryarchaeota | Methanosarcinales |
| 42 | 349307 | *Methanosaeta thermophila PT* | * Euryarchaeota | Methanosarcinales |
| 43 | 679901 | *Methanosalsum zhilinae DSM 4017* | * Euryarchaeota | Methanosarcinales |
| 44 | 188937 | *Methanosarcina acetivorans C2A* | Euryarchaeota | Methanosarcinales |
| 45 | 269797 | *Methanosarcina barkeri str. Fusaro* | Euryarchaeota | Methanosarcinales |
| 46 | 192952 | *Methanosarcina mazei Go1* | * Euryarchaeota | Methanosarcinales |
| 47 | 351160 | *Methanocella arvoryzae MRE50* | * Euryarchaeota | Methanomicrobia |
| 48 | 1041930 | *Methanocella conradii HZ254* | * Euryarchaeota | Methanomicrobia |
| 49 | 304371 | *Methanocella paludicola SANAE* | * Euryarchaeota | Methanomicrobia |
| 50 | 224325 | *Archaeoglobus fulgidus DSM 4304* | * Euryarchaeota | Archaeoglobales |
| 51 | 572546 | *Archaeoglobus profundus DSM 5631* | * Euryarchaeota | Archaeoglobales |
| 52 | 693661 | *Archaeoglobus veneficus SNP6* | * Euryarchaeota | Archaeoglobales |
| 53 | 589924 | *Ferroglobus placidus DSM 10642* | * Euryarchaeota | Archaeoglobales |
| 54 | 439481 | *Aciduliprofundum boonei T469* | * Euryarchaeota | Thermoplasmatales |
| 55 | 333146 | *Ferroplasma acidarmanus fer1* | * Euryarchaeota | Thermoplasmatales |
| 56 | 263820 | *Picrophilus torridus DSM 9790* | * Euryarchaeota | Thermoplasmatales |
| 57 | 273075 | *Thermoplasma acidophilum DSM 1728* | * Euryarchaeota | Thermoplasmatales |
| 58 | 273116 | *Thermoplasma volcanium GSS1* | * Euryarchaeota | Thermoplasmatales |
| 59 | 573064 | *Methanocaldococcus fervens AG86* | Euryarchaeota | Methanococcales |
| 60 | 573063 | *Methanocaldococcus infernus ME* | * Euryarchaeota | Methanococcales |
| 61 | 243232 | *Methanocaldococcus jannaschii DSM 2661* | * Euryarchaeota | Methanococcales |
| 62 | 644281 | *Methanocaldococcus sp. FS406-22* | Euryarchaeota | Methanococcales |
| 63 | 579137 | *Methanocaldococcus vulcanius M7* | Euryarchaeota | Methanococcales |
| 64 | 419665 | *Methanococcus aeolicus Nankai-3* | * Euryarchaeota | Methanococcales |
| 65 | 402880 | *Methanococcus maripaludis C5* | Euryarchaeota | Methanococcales |
| 66 | 406327 | *Methanococcus vannielii SB* | * Euryarchaeota | Methanococcales |
| 67 | 456320 | *Methanococcus voltae A3* | Euryarchaeota | Methanococcales |
| 68 | 647113 | *Methanothermococcus okinawensis IH1* | Euryarchaeota | Methanococcales |
| 69 | 647171 | *Methanotorris formicicus Mc-S-70* | Euryarchaeota | Methanococcales |
| 70 | 880724 | *Methanotorris igneus Kol 5* | * Euryarchaeota | Methanococcales |
| 71 | 868132 | *Methanobacterium sp. AL-21* | * Euryarchaeota | Methanobacteriales |
| 72 | 634498 | *Methanobrevibacter ruminantium M1* | Euryarchaeota | Methanobacteriales |
| 73 | 420247 | *Methanobrevibacter smithii ATCC 35061* | * Euryarchaeota | Methanobacteriales |
| 74 | 339860 | *Methanosphaera stadtmanae DSM 3091* | * Euryarchaeota | Methanobacteriales |

| | | | | | |
|---|---|---|---|---|---|
| 75 | 79929 | *Methanothermobacter marburgensis str. Marburg* | | Euryarchaeota | Methanobacteriales |
| 76 | 187420 | *Methanothermobacter thermautotrophicus Delta H* | * | Euryarchaeota | Methanobacteriales |
| 77 | 523846 | *Methanothermus fervidus DSM 2088* | * | Euryarchaeota | Methanobacteriales |
| 78 | 190192 | *Methanopyrus kandleri AV19* | * | Euryarchaeota | Methanopyrales |
| 79 | 272844 | *Pyrococcus abyssi GE5* | * | Euryarchaeota | Thermococcales |
| 80 | 186497 | *Pyrococcus furiosus DSM 3638* | | Euryarchaeota | Thermococcales |
| 81 | 70601 | *Pyrococcus horikoshii OT3* | | Euryarchaeota | Thermococcales |
| 82 | 342949 | *Pyrococcus sp. NA2* | | Euryarchaeota | Thermococcales |
| 83 | 529709 | *Pyrococcus yayanosii CH1* | * | Euryarchaeota | Thermococcales |
| 84 | 391623 | *Thermococcus barophilus MP* | * | Euryarchaeota | Thermococcales |
| 85 | 593117 | *Thermococcus gammatolerans EJ3* | * | Euryarchaeota | Thermococcales |
| 86 | 69014 | *Thermococcus kodakarensis KOD1* | | Euryarchaeota | Thermococcales |
| 87 | 523849 | *Thermococcus litoralis DSM 5473* | * | Euryarchaeota | Thermococcales |
| 88 | 523850 | *Thermococcus onnurineus NA1* | | Euryarchaeota | Thermococcales |
| 89 | 604354 | *Thermococcus sibiricus MM 739* | | Euryarchaeota | Thermococcales |
| 90 | 246969 | *Thermococcus sp. AM4* | | Euryarchaeota | Thermococcales |
| 91 | 425595 | *Candidatus* Micrarchaeum acidiphilum ARMAN-2 | * | Euryarchaeota | ARMAN |
| 92 | 662760 | *Candidatus* Parvarchaeum acidiphilum ARMAN-4 | * | Euryarchaeota | ARMAN |
| 93 | 662762 | *Candidatus* Parvarchaeum acidophilus ARMAN-5 | * | Euryarchaeota | ARMAN |
| 94 | 228908 | *Nanoarchaeum equitans Kin4-M* | * | Euryarchaeota | Nanoarchaeota |
| 95 | 274854 | uncultured marine group II euryarchaeote | * | Euryarchaeota | Unclassified |
| 96 | 666510 | *Acidilobus saccharovorans 345-15* | * | Crenarchaeota | Desulfurococcales |
| 97 | 272557 | *Aeropyrum pernix K1* | * | Crenarchaeota | Desulfurococcales |
| 98 | 768672 | *Desulfurococcus fermentans DSM 16532* | | Crenarchaeota | Desulfurococcales |
| 99 | 490899 | *Desulfurococcus kamchatkensis 1221n* | * | Crenarchaeota | Desulfurococcales |
| 100 | 765177 | *Desulfurococcus mucosus DSM 2162* | | Crenarchaeota | Desulfurococcales |
| 101 | 415426 | *Hyperthermus butylicus DSM 5456* | * | Crenarchaeota | Desulfurococcales |
| 102 | 453591 | *Ignicoccus hospitalis KIN4/I* | * | Crenarchaeota | Desulfurococcales |
| 103 | 583356 | *Ignisphaera aggregans DSM 17230* | * | Crenarchaeota | Desulfurococcales |
| 104 | 694429 | *Pyrolobus fumarii 1A* | * | Crenarchaeota | Desulfurococcales |
| 105 | 591019 | *Staphylothermus hellenicus DSM 12710* | * | Crenarchaeota | Desulfurococcales |
| 106 | 399550 | *Staphylothermus marinus F1* | | Crenarchaeota | Desulfurococcales |
| 107 | 633148 | *Thermosphaera aggregans DSM 11486* | * | Crenarchaeota | Desulfurococcales |
| 108 | 933801 | *Acidianus hospitalis W1* | * | Crenarchaeota | Sulfolobales |
| 109 | 1006006 | *Metallosphaera cuprina Ar-4* | | Crenarchaeota | Sulfolobales |
| 110 | 399549 | *Metallosphaera sedula DSM 5348* | * | Crenarchaeota | Sulfolobales |

| | | | | | |
|---|---|---|---|---|---|
| 111 | 671065 | *Metallosphaera yellowstonensis MK1* | * | Crenarchaeota | Sulfolobales |
| 112 | 330779 | *Sulfolobus acidocaldarius DSM 639* | * | Crenarchaeota | Sulfolobales |
| 113 | 429572 | *Sulfolobus islandicus L.S.2.15* | | Crenarchaeota | Sulfolobales |
| 114 | 273057 | *Sulfolobus solfataricus P2* | * | Crenarchaeota | Sulfolobales |
| 115 | 273063 | *Sulfolobus tokodaii str. 7* | * | Crenarchaeota | Sulfolobales |
| | | | | | |
| 116 | 397948 | *Caldivirga maquilingensis IC-167* | * | Crenarchaeota | Thermoproteales |
| 117 | 178306 | *Pyrobaculum aerophilum str. IM2* | * | Crenarchaeota | Thermoproteales |
| 118 | 340102 | *Pyrobaculum arsenaticum DSM 13514* | * | Crenarchaeota | Thermoproteales |
| 119 | 410359 | *Pyrobaculum calidifontis JCM 11548* | | Crenarchaeota | Thermoproteales |
| 120 | 384616 | *Pyrobaculum islandicum DSM 4184* | * | Crenarchaeota | Thermoproteales |
| 121 | 698757 | *Pyrobaculum oguniense TE7* | | Crenarchaeota | Thermoproteales |
| 122 | 1104324 | *Pyrobaculum sp. 1860* | | Crenarchaeota | Thermoproteales |
| 123 | 368408 | *Thermofilum pendens Hrk 5* | * | Crenarchaeota | Thermoproteales |
| 124 | 444157 | *Thermoproteus neutrophilus V24Sta* | | Crenarchaeota | Thermoproteales |
| 125 | 768679 | *Thermoproteus tenax Kra 1* | * | Crenarchaeota | Thermoproteales |
| 126 | 999630 | *Thermoproteus uzoniensis 768-20* | | Crenarchaeota | Thermoproteales |
| 127 | 572478 | *Vulcanisaeta distributa DSM 14429* | * | Crenarchaeota | Thermoproteales |
| 128 | 985053 | *Vulcanisaeta moutnovskia 768-28* | | Crenarchaeota | Thermoproteales |
| | | | | | |
| 129 | 374847 | *Candidatus* Korarchaeum cryptofilum OPF8 | * | Korachaeota | |
| | | | | | |
| 130 | 134676 | *Actinoplanes sp. SE50/110* | | Actinobacteria | Actinobacteria |
| 131 | 1133849 | *Nocardia brasiliensis ATCC 700358* | * | Actinobacteria | Actinobacteria |
| 132 | 101510 | *Rhodococcus jostii RHA1* | | Actinobacteria | Actinobacteria |
| 133 | 1179773 | *Saccharothrix espanaensis DSM 44229* | | Actinobacteria | Actinobacteria |
| 134 | 463191 | *Streptomyces sviceus ATCC 29083* | | Actinobacteria | Actinobacteria |
| | | | | | |
| 135 | 224324 | *Aquifex aeolicus VF5* | | Aquificae | Aquificae |
| 136 | 608538 | *Hydrogenobacter thermophilus TK-6* | | Aquificae | Aquificae |
| 137 | 123214 | *Persephonella marina EX-H1* | * | Aquificae | Aquificae |
| 138 | 436114 | *Sulfurihydrogenibium sp. YO3AOP1* | | Aquificae | Aquificae |
| 139 | 648996 | *Thermovibrio ammonificans HB-1* | | Aquificae | Aquificae |
| | | | | | |
| 140 | 485918 | *Chitinophaga pinensis DSM 2588* | | Bacteroidetes/Chlorobi | Bacteroidetes |
| 141 | 760192 | *Haliscomenobacter hydrossis DSM 1100* | | Bacteroidetes/Chlorobi | Bacteroidetes |
| 142 | 700598 | *Niastella koreensis GR20-10* | | Bacteroidetes/Chlorobi | Bacteroidetes |
| 143 | 290317 | *Chlorobium phaeobacteroides DSM 266* | * | Bacteroidetes/Chlorobi | Chlorobi |
| 144 | 517418 | *Chloroherpeton thalassium ATCC 35110* | | Bacteroidetes/Chlorobi | Chlorobi |
| 145 | 945713 | *Ignavibacterium album JCM 16511* | | Bacteroidetes/Chlorobi | Ignavibacteria |
| | | | | | |
| 146 | 511051 | *Caldisericum exile AZM16c01* | | Caldiserica | Caldisericia |

| | | | | | |
|---|---|---|---|---|---|
| 147 | 765952 | *Parachlamydia acanthamoebae UV-7* | | Chlamydiae/Verrucomicrobia | Chlamydiae |
| 148 | 331113 | *Simkania negevensis Z* | | Chlamydiae/Verrucomicrobia | Chlamydiae |
| 149 | 716544 | *Waddlia chondrophila WSU 86-1044* | | Chlamydiae/Verrucomicrobia | Chlamydiae |
| 150 | 583355 | *Coraliomargarita akajimensis DSM 45221* | | Chlamydiae/Verrucomicrobia | Verrucomicrobia |
| 151 | 452637 | *Opitutus terrae PB90-1* | * | Chlamydiae/Verrucomicrobia | Verrucomicrobia |
| 152 | 926569 | *Anaerolinea thermophila UNI-1* | | Chloroflexi | Anaerolineae |
| 153 | 926550 | *Caldilinea aerophila DSM 14535 = NBRC 104270* | | Chloroflexi | Caldilineae |
| 154 | 326427 | *Chloroflexus aggregans DSM 9485* | | Chloroflexi | Chloroflexi |
| 155 | 552811 | *Dehalogenimonas lykanthroporepellens BL-DC-9* | * | Chloroflexi | Dehalococcoidetes |
| 156 | 309801 | *Thermomicrobium roseum DSM 5159* | | Chloroflexi | Thermomicrobia |
| 157 | 653733 | *Desulfurispirillum indicum S5* | * | Chrysiogenetes | Chrysiogenetes |
| 158 | 329726 | *Acaryochloris marina MBIC11017* | | Cyanobacteria | Chroococcales |
| 159 | 251221 | *Gloeobacter violaceus PCC 7421* | | Cyanobacteria | Gloeobacteria |
| 160 | 63737 | *Nostoc punctiforme PCC 73102* | | Cyanobacteria | Nostocales |
| 161 | 179408 | *Oscillatoria nigro-viridis PCC 7112* | * | Cyanobacteria | Oscillatoriales |
| 162 | 251229 | *Chroococcidiopsis thermalis PCC 7203* | | Cyanobacteria | Pleurocapsales |
| 163 | 59922 | *Prochlorococcus marinus str. MIT 9303* | | Cyanobacteria | Prochlorales |
| 164 | 768670 | *Calditerrivibrio nitroreducens DSM 19672* | * | Deferribacteres | Deferribacteres |
| 165 | 639282 | *Deferribacter desulfuricans SSM1* | | Deferribacteres | Deferribacteres |
| 166 | 522772 | *Denitrovibrio acetiphilus DSM 12809* | | Deferribacteres | Deferribacteres |
| 167 | 717231 | *Flexistipes sinusarabici DSM 4947* | | Deferribacteres | Deferribacteres |
| 168 | 937777 | *Deinococcus peraridilitoris DSM 19664* | | Deinococcus-Thermus | Deinococci |
| 169 | 526227 | *Meiothermus silvanus DSM 9946* | * | Deinococcus-Thermus | Deinococci |
| 170 | 670487 | *Oceanithermus profundus DSM 14977* | | Deinococcus-Thermus | Deinococci |
| 171 | 743525 | *Thermus scotoductus SA-01* | | Deinococcus-Thermus | Deinococci |
| 172 | 649638 | *Truepera radiovictrix DSM 17093* | | Deinococcus-Thermus | Deinococci |
| 173 | 309799 | *Dictyoglomus thermophilum H-6-12* | | Dictyoglomi | Dictyoglomia |
| 174 | 515635 | *Dictyoglomus turgidum DSM 6724* | * | Dictyoglomi | Dictyoglomia |
| 175 | 445932 | *Elusimicrobium minutum Pei191* | | Elusimicrobia | Elusimicrobia |
| 176 | 471821 | uncultured Termite group 1 bacterium Rs-D17 | * | Elusimicrobia | environmental samples |
| 177 | 240015 | *Acidobacterium capsulatum ATCC 51196* | | Fibrobacteres/Acidobacteria | Acidobacteria |
| 178 | 234267 | *Candidatus* Solibacter usitatus Ellin6076 | * | Fibrobacteres/Acidobacteria | Acidobacteria |
| 179 | 682795 | *Granulicella mallensis MP5ACTX8* | | Fibrobacteres/Acidobacteria | Acidobacteria |
| 180 | 926566 | *Terriglobus roseus DSM 18391* | | Fibrobacteres/Acidobacteria | Acidobacteria |

| | | | | | |
|---|---|---|---|---|---|
| 181 | 59374 | *Fibrobacter succinogenes subsp. Succinogenes* | | Fibrobacteres/Acidobacteria | Fibrobacteres |
| 182 | 1195464 | *Bacillus thuringiensis MC28* | | Firmicutes | Bacilli |
| 183 | 997761 | *Paenibacillus mucilaginosus K02* | | Firmicutes | Bacilli |
| 184 | 573061 | *Clostridium cellulovorans 743B* | | Firmicutes | Clostridia |
| 185 | 768706 | *Desulfosporosinus orientis DSM 765* | * | Firmicutes | Clostridia |
| 186 | 650150 | *Erysipelothrix rhusiopathiae* | | Firmicutes | Erysipelotrichi |
| 187 | 479436 | *Veillonella parvula DSM 2008* | | Firmicutes | Negativicutes |
| 188 | 190304 | *Fusobacterium nucleatum subsp. Nucleatum* | | Fusobacteria | Fusobacteriia |
| 189 | 572544 | *Ilyobacter polytropus DSM 2926* | | Fusobacteria | Fusobacteriia |
| 190 | 523794 | *Leptotrichia buccalis C-1013-b* | | Fusobacteria | Fusobacteriia |
| 191 | 526218 | *Sebaldella termitidis ATCC 33386* | * | Fusobacteria | Fusobacteriia |
| 192 | 519441 | *Streptobacillus moniliformis DSM 12112* | | Fusobacteria | Fusobacteriia |
| 193 | 379066 | *Gemmatimonas aurantiaca T-27* | | Gemmatimonadetes | Gemmatimonadetes |
| 194 | 330214 | *Candidatus* Nitrospira defluvii | | Nitrospirae | Nitrospira |
| 195 | 1048260 | *Leptospirillum ferriphilum ML-04* | | Nitrospirae | Nitrospira |
| 196 | 1162668 | *Leptospirillum ferrooxidans C2-3* | * | Nitrospirae | Nitrospira |
| 197 | 289376 | *Thermodesulfovibrio yellowstonii DSM 11347* | | Nitrospirae | Nitrospira |
| 198 | 1142394 | *Phycisphaera mikurensis NBRC 102666* | | Planctomycetes | Phycisphaerae |
| 199 | 530564 | *Pirellula staleyi DSM 6068* | | Planctomycetes | Planctomycetia |
| 200 | 756272 | *Planctomyces brasiliensis DSM 5305* | * | Planctomycetes | Planctomycetia |
| 201 | 243090 | *Rhodopirellula baltica SH 1* | | Planctomycetes | Planctomycetia |
| 202 | 886293 | *Singulisphaera acidiphila DSM 18658* | | Planctomycetes | Planctomycetia |
| 203 | 311403 | *Agrobacterium radiobacter K84* | | Proteobacteria | Alphaproteobacteria |
| 204 | 137722 | *Azospirillum sp. B510* | * | Proteobacteria | Alphaproteobacteria |
| 205 | 1037409 | *Bradyrhizobium japonicum USDA 6* | | Proteobacteria | Alphaproteobacteria |
| 206 | 460265 | *Methylobacterium nodulans ORS 2060* | | Proteobacteria | Alphaproteobacteria |
| 207 | 216596 | *Rhizobium leguminosarum bv. viciae 3841* | | Proteobacteria | Alphaproteobacteria |
| 208 | 762376 | *Achromobacter xylosoxidans A8* | | Proteobacteria | Betaproteobacteria |
| 209 | 266265 | *Burkholderia xenovorans LB400* | * | Proteobacteria | Betaproteobacteria |
| 210 | 1042878 | *Cupriavidus necator N-1* | | Proteobacteria | Betaproteobacteria |
| 211 | 398578 | *Delftia acidovorans SPH-1* | | Proteobacteria | Betaproteobacteria |
| 212 | 381666 | *Ralstonia eutropha H16* | | Proteobacteria | Betaproteobacteria |
| 213 | 572480 | *Arcobacter nitrofigilis DSM 7299* | * | Proteobacteria | delta/epsilon subdivisions |
| 214 | 706587 | *Desulfomonile tiedjei DSM 6799* | * | Proteobacteria | delta/epsilon subdivisions |
| 215 | 502025 | *Haliangium ochraceum DSM 14365* | | Proteobacteria | delta/epsilon subdivisions |

| | | | | | |
|---|---|---|---|---|---|
| 216 | 246197 | *Myxococcus xanthus DK 1622* | | Proteobacteria | delta/epsilon subdivisions |
| 217 | 709032 | *Sulfuricurvum kujiense DSM 16994* | | Proteobacteria | delta/epsilon subdivisions |
| 218 | 349521 | *Hahella chejuensis KCTC 2396* | * | Proteobacteria | Gammaproteobacteria |
| 219 | 1191061 | *Klebsiella oxytoca E718* | | Proteobacteria | Gammaproteobacteria |
| 220 | 592316 | *Pantoea sp. At-9b* | | Proteobacteria | Gammaproteobacteria |
| 221 | 220664 | *Pseudomonas protegens Pf-5* | | Proteobacteria | Gammaproteobacteria |
| 222 | 338187 | *Vibrio harveyi ATCC BAA-1116* | | Proteobacteria | Gammaproteobacteria |
| 223 | 573825 | *Leptospira interrogans serovar Lai str. IPAV* | * | Spirochaetes | Spirochaetia |
| 224 | 158190 | *Sphaerochaeta pleomorpha str. Grapes* | | Spirochaetes | Spirochaetia |
| 225 | 573413 | *Spirochaeta smaragdinae DSM 11293* | | Spirochaetes | Spirochaetia |
| 226 | 545694 | *Treponema primitia ZAS-2* | | Spirochaetes | Spirochaetia |
| 227 | 869212 | *Turneriella parva DSM 21527* | | Spirochaetes | Spirochaetia |
| 228 | 572547 | *Aminobacterium colombiense DSM 12261* | | Synergistetes | Synergistia |
| 229 | 584708 | *Aminomonas paucivorans DSM 12260* | | Synergistetes | Synergistia |
| 230 | 891968 | *Anaerobaculum mobile DSM 13181* | * | Synergistetes | Synergistia |
| 231 | 580340 | *Thermovirga lienii DSM 17291* | | Synergistetes | Synergistia |
| 232 | 441768 | *Acholeplasma laidlawii PG-8A* | | Tenericutes | Mollicutes |
| 233 | 322098 | *Aster yellows witches'-broom phytoplasma AYWB* | | Tenericutes | Mollicutes |
| 234 | 265311 | *Mesoplasma florum L1* | | Tenericutes | Mollicutes |
| 235 | 272633 | *Mycoplasma penetrans HF-2* | | Tenericutes | Mollicutes |
| 236 | 565575 | *Ureaplasma urealyticum serovar 10 ATCC 33699* | | Tenericutes | Mollicutes |
| 237 | 667014 | *Thermodesulfatator indicus DSM 15286* | * | Thermodesulfobacteria | Thermodesulfobacteria |
| 238 | 795359 | *Thermodesulfobacterium sp. OPB45* | | Thermodesulfobacteria | Thermodesulfobacteria |
| 239 | 521045 | *Kosmotoga olearia TBF 19.5.1* | | Thermotogae | Thermotogae |
| 240 | 443254 | *Marinitoga piezophila KA3* | | Thermotogae | Thermotogae |
| 241 | 660470 | *Mesotoga prima MesG1.Ag.4.2* | | Thermotogae | Thermotogae |
| 242 | 403833 | *Petrotoga mobilis SJ95* | | Thermotogae | Thermotogae |
| 243 | 126740 | *Thermotoga sp. RQ2* | * | Thermotogae | Thermotogae |
| 244 | 880073 | *Caldithrix abyssi DSM 13497* | | unclassified Bacteria | Caldithrix |
| 245 | 671143 | *Candidatus* Methylomirabilis oxyfera | * | unclassified Bacteria | candidate division NC10 |
| 246 | 525904 | *Thermobaculum terrenum ATCC BAA-798* | * | unclassified Bacteria | Thermobaculum |

**Supplementary table S2.** List of all protein markers used in this work.

| Annotation | gi number | Accession # | COG number | COG class | COG definition |
|---|---|---|---|---|---|
| argininosuccinate synthase | 161528794 | YP_001582620.1 | COG0137 | E | Amino acid transport and metabolism |
| homoserine kinase | 161527553 | YP_001581379.1 | COG0083 | E | Amino acid transport and metabolism |
| homoserine dehydrogenase | 161528204 | YP_001582030.1 | COG0460 | E | Amino acid transport and metabolism |
| aspartate kinase | 161529262 | YP_001583088.1 | COG0527 | E | Amino acid transport and metabolism |
| aspartate carbamoyltransferase | 161529194 | YP_001583020.1 | COG0540 | F | Nucleotide transport and metabolism |
| phosphoribosylformylglycinamidine cyclo-ligase | 161528090 | YP_001581916.1 | COG0150 | F | Nucleotide transport and metabolism |
| uridylate kinase putative | 161529213 | YP_001583039.1 | COG0528 | F | Nucleotide transport and metabolism |
| phosphoribosylformylglycinamidine synthase II | 161528288 | YP_001582114.1 | COG0046 | F | Nucleotide transport and metabolism |
| adenylosuccinate lyase | 161528963 | YP_001582789.1 | COG0015 | F | Nucleotide transport and metabolism |
| RdgB/HAM1 family non-canonical purine NTP pyrophosphatase | 161529038 | YP_001582864.1 | COG0127 | F | Nucleotide transport and metabolism |
| glutamine amidotransferase class-II | 161528289 | YP_001582115.1 | COG0034 | F | Nucleotide transport and metabolism |
| phosphoglycerate kinase | 161528008 | YP_001581834.1 | COG0126 | G | Carbohydrate transport and metabolism |
| phosphopantothenoylcysteine decarboxylase | 161529229 | YP_001583055.1 | COG0452 | H | Coenzyme transport and metabolism |
| pyridoxine biosynthesis protein | 161528987 | YP_001582813.1 | COG0214 | H | Coenzyme transport and metabolism |
| UbiD family decarboxylase | 161529105 | YP_001582931.1 | COG0043 | H | Coenzyme transport and metabolism |
| porphobilinogen deaminase | 118576540 | YP_876283.1 | COG0181 | H | Coenzyme transport and metabolism |
| glutamate-1-semialdehyde-2 1-aminomutase | 161527998 | YP_001581824.1 | COG0001 | H | Coenzyme transport and metabolism |
| molybdenum cofactor biosynthesis protein C | 315427188 | BAJ48802.1 | COG0315 | H | Coenzyme transport and metabolism |
| conserved hypothetical protein | 315426923 | BAJ48542.1 | COG3425 | I | Lipid transport and metabolism |
| beta-lactamase domain-containing protein | 161529047 | YP_001582873.1 | COG1236 | J | Translation, ribosomal structure and biogenesis |
| glutamyl-tRNA(Gln) amidotransferase subunit E | 161527611 | YP_001581437.1 | COG2511 | J | Translation, ribosomal structure and biogenesis |
| tRNA-guanine transglycosylase | 161529044 | YP_001582870.1 | COG0343 | J | Translation, ribosomal structure and biogenesis |
| glutamyl-tRNA(Gln) amidotransferase B subunit | 161528376 | YP_001582202.1 | COG0064 | J | Translation, ribosomal structure and biogenesis |
| exosome complex exonuclease 1 | 161527940 | YP_001581766.1 | COG0689 | J | Translation, ribosomal structure and biogenesis |
| MiaB-like tRNA modifying enzyme | 161528769 | YP_001582595.1 | COG0621 | J | Translation, ribosomal structure and biogenesis |
| phenylalanyl-tRNA synthetase alpha subunit | 161528997 | YP_001582823.1 | COG0016 | J | Translation, ribosomal structure and biogenesis |
| prolyl-tRNA synthetase | 315425642 | BAJ47301.1 | COG0442 | J | Translation, ribosomal structure and biogenesis |
| histidyl-tRNA synthetase | 315425704 | BAJ47360.1 | COG0124 | J | Translation, ribosomal structure and biogenesis |
| 5-nucleotidase SurE | 315426747 | BAJ48371.1 | COG0522 | J | Translation, ribosomal structure and biogenesis |
| peptidase M50 | 161527799 | YP_001581625.1 | COG0750 | M | Cell wall/membrane/envelope biogenesis |
| cytidyltransferase-like protein | 161528806 | YP_001582632.1 | COG0615 | MI | Cell wall/membrane/envelope biogenesis |
| metalloendopeptidase glycoprotease family | 161529041 | YP_001582867.1 | COG0533 | O | Post-translational modification, protein turnover, and chaperones |
| hydrogenase maturation protein HypF | 315426449 | BAJ48087.1 | COG0068 | O | Post-translational modification, protein turnover, and chaperones |
| hydrogenase expression formation protein HypD | 315426462 | BAJ48095.1 | COG0409 | O | Post-translational modification, protein turnover, and chaperones |
| GTP1/OBG protein | 161529113 | YP_001582939.1 | COG2262 | R | General function prediction only |
| LPPG:FO 2-phospho-L-lactate transferase | 161528134 | YP_001581960.1 | COG0391 | S | Function unknown |
| GTP-binding signal recognition particle | 161528039 | YP_001581865.1 | COG0541 | U | Intracellular trafficking, secretion, and vesicular transport |
| signal recognition particle receptor | 315425933 | BAJ47583.1 | COG0552 | U | Intracellular trafficking, secretion, and vesicular transport |

| Annotation | gi number | Accession # | COG number | COG class | COG definition |
|---|---|---|---|---|---|
| 50S ribosomal protein L1 | 161527890 | YP_001581716 | | J | Translation, ribosomal structure and biogenesis |
| 50S ribosomal protein L2 | 161527614 | YP_001581440 | | J | Translation, ribosomal structure and biogenesis |
| 50S ribosomal protein L3 | 161528317 | YP_001582143 | | J | Translation, ribosomal structure and biogenesis |
| 50S ribosomal protein L4 | 161528316 | YP_001582142 | | J | Translation, ribosomal structure and biogenesis |
| 50S ribosomal protein L5 | 161528305 | YP_001582131 | | J | Translation, ribosomal structure and biogenesis |
| 50S ribosomal protein L6 | 161528302 | YP_001582128 | | J | Translation, ribosomal structure and biogenesis |
| acidic ribosomal protein P0 – L10 | 161527889 | YP_001581715 | | J | Translation, ribosomal structure and biogenesis |
| 50S ribosomal protein L10e | 161527957 | YP_001581783 | | J | Translation, ribosomal structure and biogenesis |
| 50S ribosomal protein L11 | 161527893 | YP_001581719 | | J | Translation, ribosomal structure and biogenesis |
| 50S ribosomal protein L13 | 161527934 | YP_001581760 | | J | Translation, ribosomal structure and biogenesis |
| 50S ribosomal protein L14 | 161528308 | YP_001582134 | | J | Translation, ribosomal structure and biogenesis |
| 50S ribosomal protein L15 | 161527909 | YP_001581735 | | J | Translation, ribosomal structure and biogenesis |
| 50S ribosomal protein L18 | 161527906 | YP_001581732 | | J | Translation, ribosomal structure and biogenesis |
| 50S ribosomal protein L22 | 161528313 | YP_001582139 | | J | Translation, ribosomal structure and biogenesis |
| 50S ribosomal protein L25 | 161528315 | YP_001582141 | | J | Translation, ribosomal structure and biogenesis |
| 50S ribosomal protein L24 | 161528307 | YP_001582133 | | J | Translation, ribosomal structure and biogenesis |
| 50S ribosomal protein L29 | 161528311 | YP_001582137 | | J | Translation, ribosomal structure and biogenesis |
| 50S ribosomal protein L30 | 161527908 | YP_001581734 | | J | Translation, ribosomal structure and biogenesis |
| 30S ribosomal protein S2 | 161527824 | YP_001581650 | | J | Translation, ribosomal structure and biogenesis |
| 30S ribosomal protein S3 | 161528312 | YP_001582138 | | J | Translation, ribosomal structure and biogenesis |
| 30S ribosomal protein S4 | 161527832 | YP_001581658 | | J | Translation, ribosomal structure and biogenesis |
| 30S ribosomal protein S5 | 161527907 | YP_001581733 | | J | Translation, ribosomal structure and biogenesis |
| 30S ribosomal protein S7 | 161527863 | YP_001581689 | | J | Translation, ribosomal structure and biogenesis |
| 30S ribosomal protein S8 | 161528303 | YP_001582129 | | J | Translation, ribosomal structure and biogenesis |
| 30S ribosomal protein S9 | 161527933 | YP_001581759 | | J | Translation, ribosomal structure and biogenesis |
| 30S ribosomal protein S10 | 161528541 | YP_001582367 | | J | Translation, ribosomal structure and biogenesis |
| 30S ribosomal protein S11 | 161528958 | YP_001582784 | | J | Translation, ribosomal structure and biogenesis |
| 30S ribosomal protein S12 | 161527862 | YP_001581688 | | J | Translation, ribosomal structure and biogenesis |
| 30S ribosomal protein S13 | 161527833 | YP_001581659 | | J | Translation, ribosomal structure and biogenesis |
| 30S ribosomal protein S15 | 161529016 | YP_001582842 | | J | Translation, ribosomal structure and biogenesis |
| 30S ribosomal protein S17 | 161528309 | YP_001582135 | | J | Translation, ribosomal structure and biogenesis |
| 30S ribosomal protein S19 | 161528314 | YP_001582140 | | J | Translation, ribosomal structure and biogenesis |

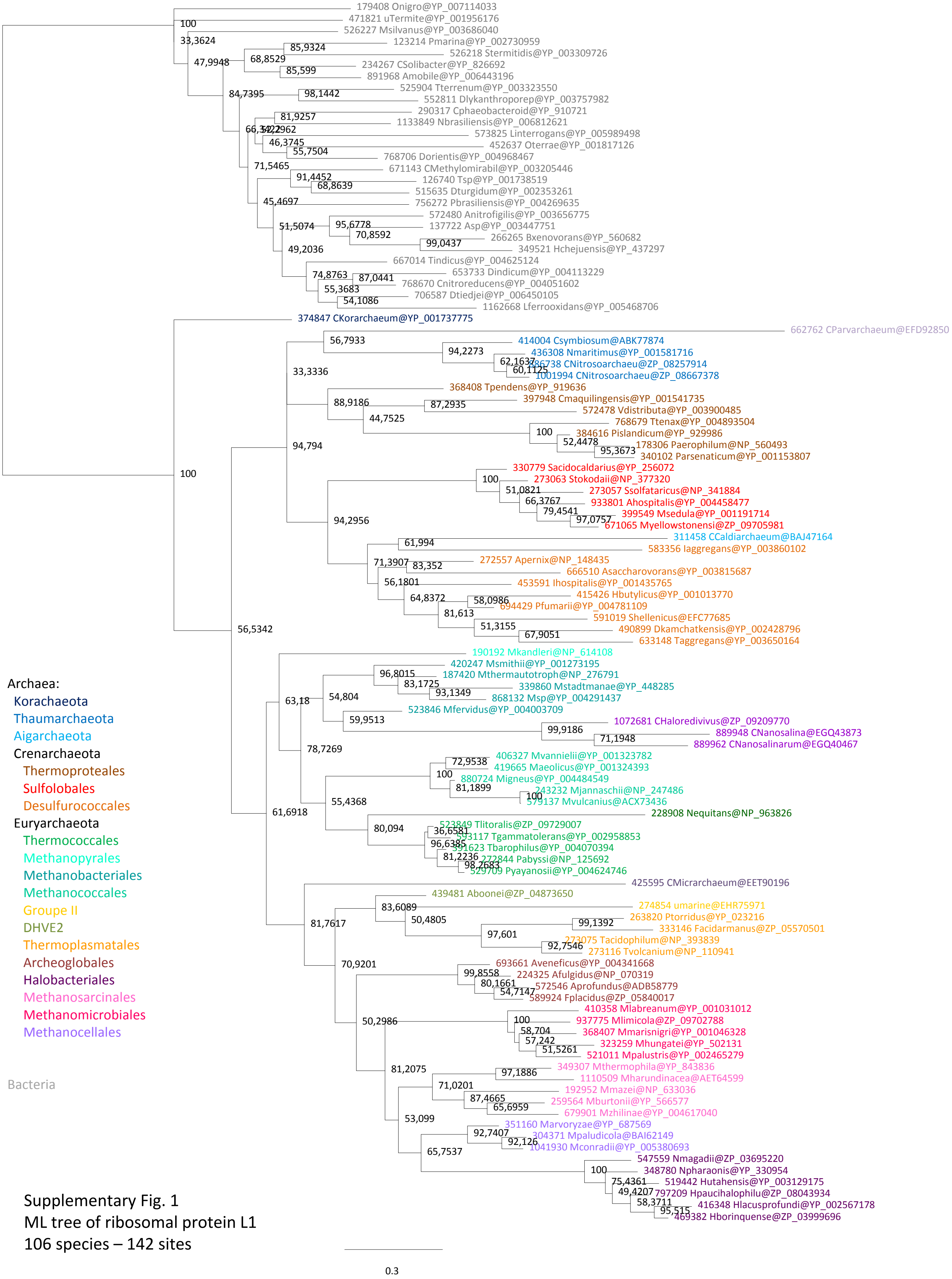


Supplementary Fig. 1
ML tree of ribosomal protein L1
106 species – 142 sites

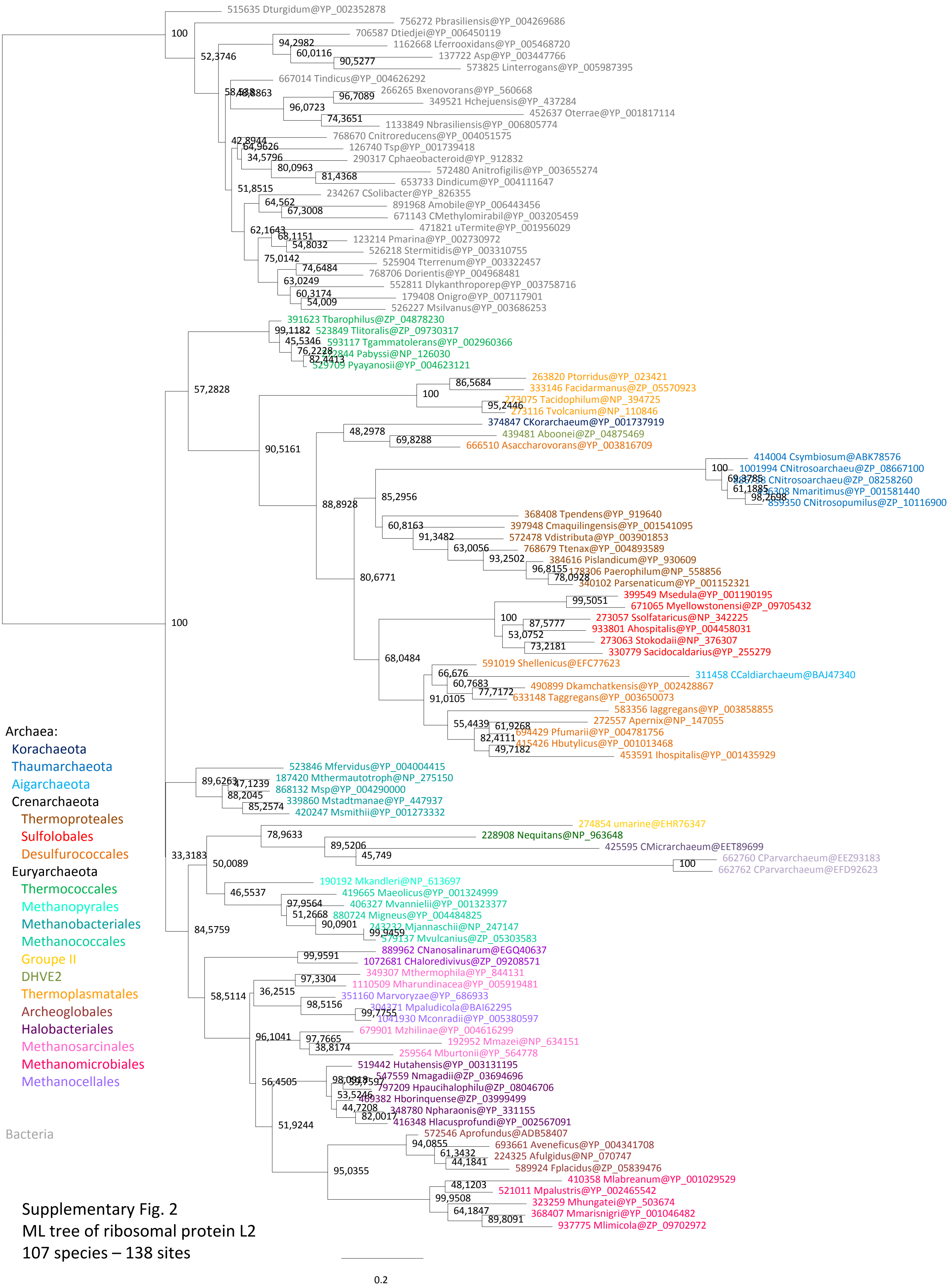


Supplementary Fig. 2
ML tree of ribosomal protein L2
107 species – 138 sites

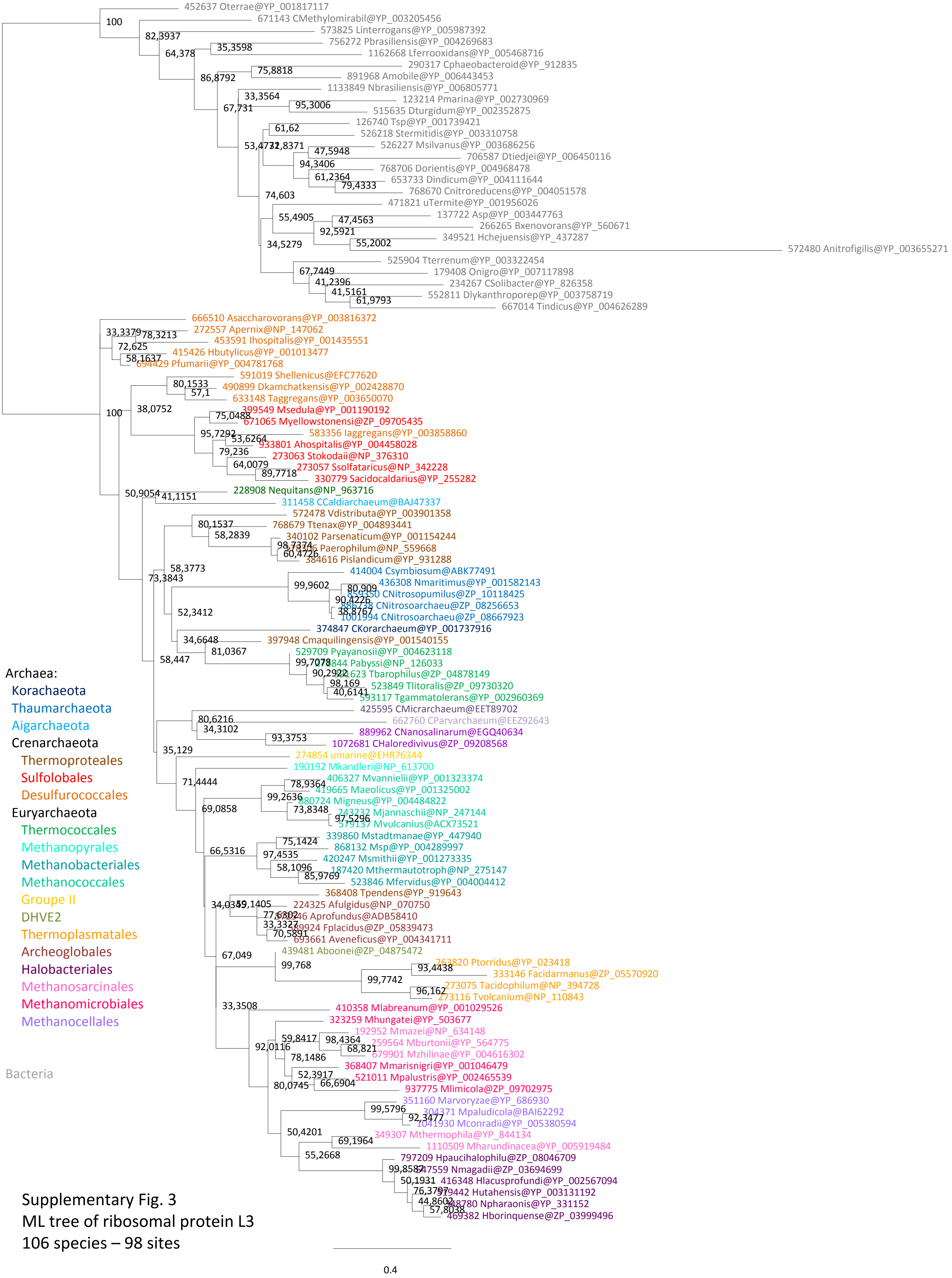


Supplementary Fig. 3
ML tree of ribosomal protein L3
106 species – 98 sites

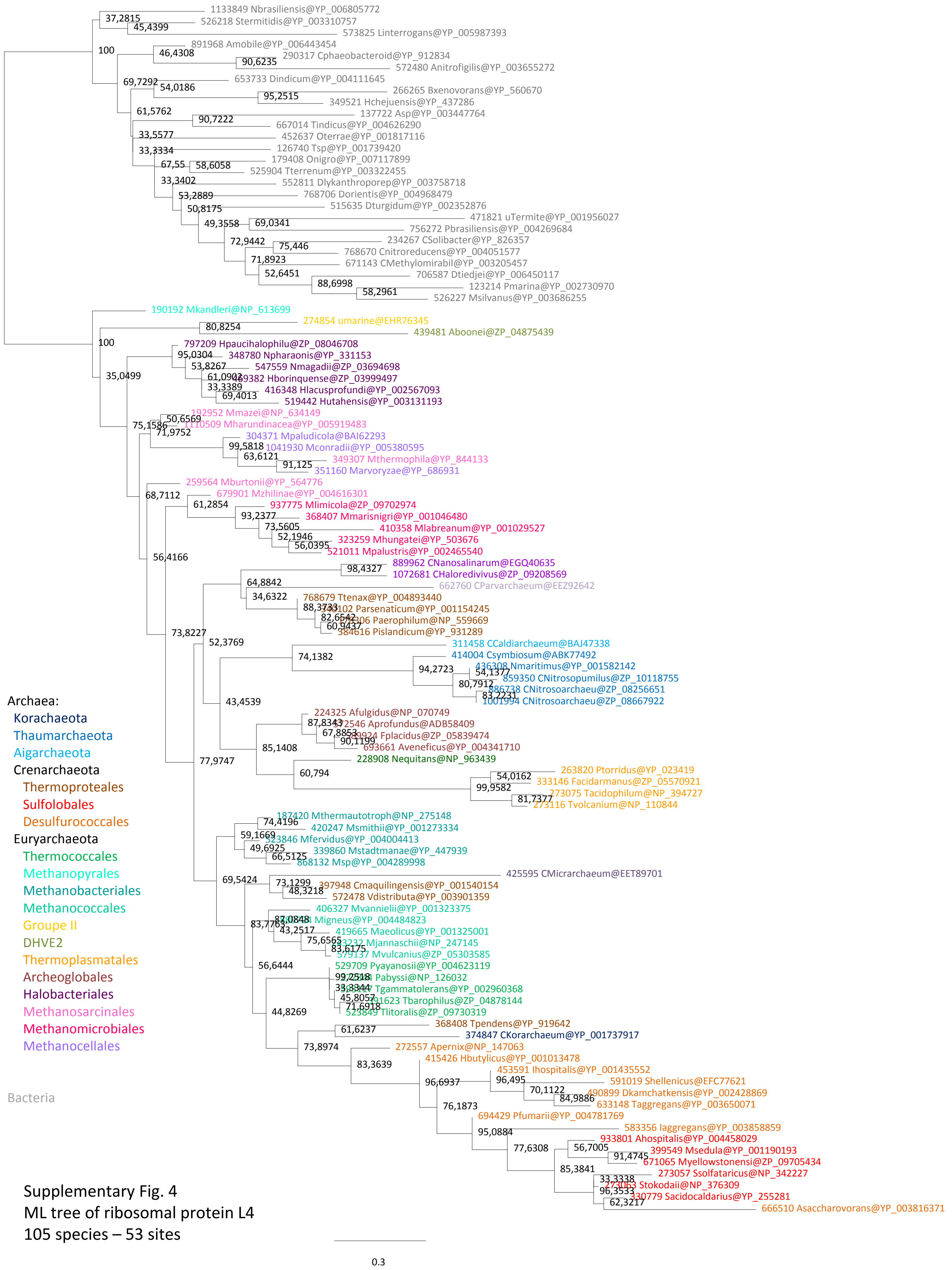


Supplementary Fig. 4
ML tree of ribosomal protein L4
105 species – 53 sites

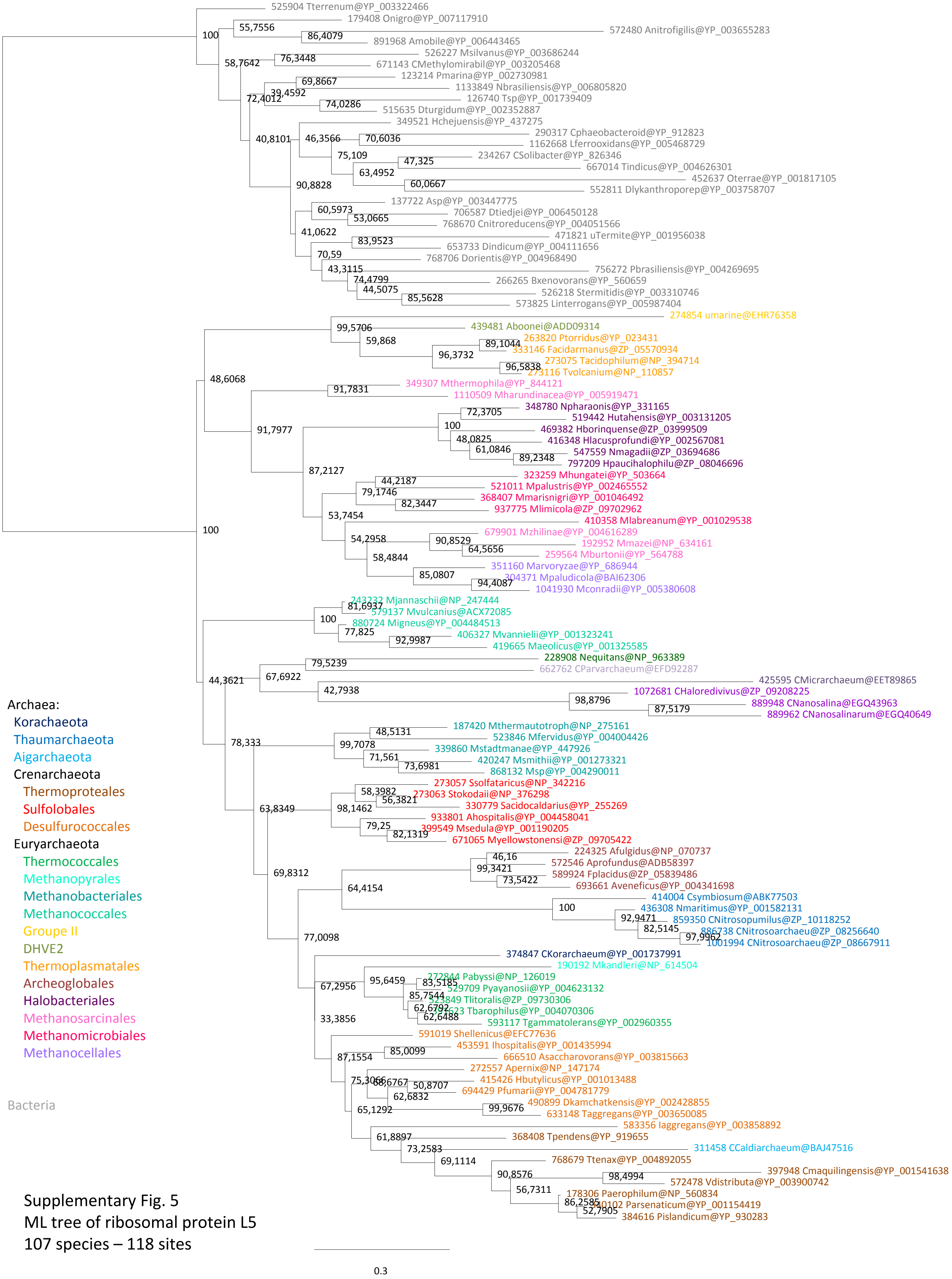


Supplementary Fig. 5
ML tree of ribosomal protein L5
107 species – 118 sites

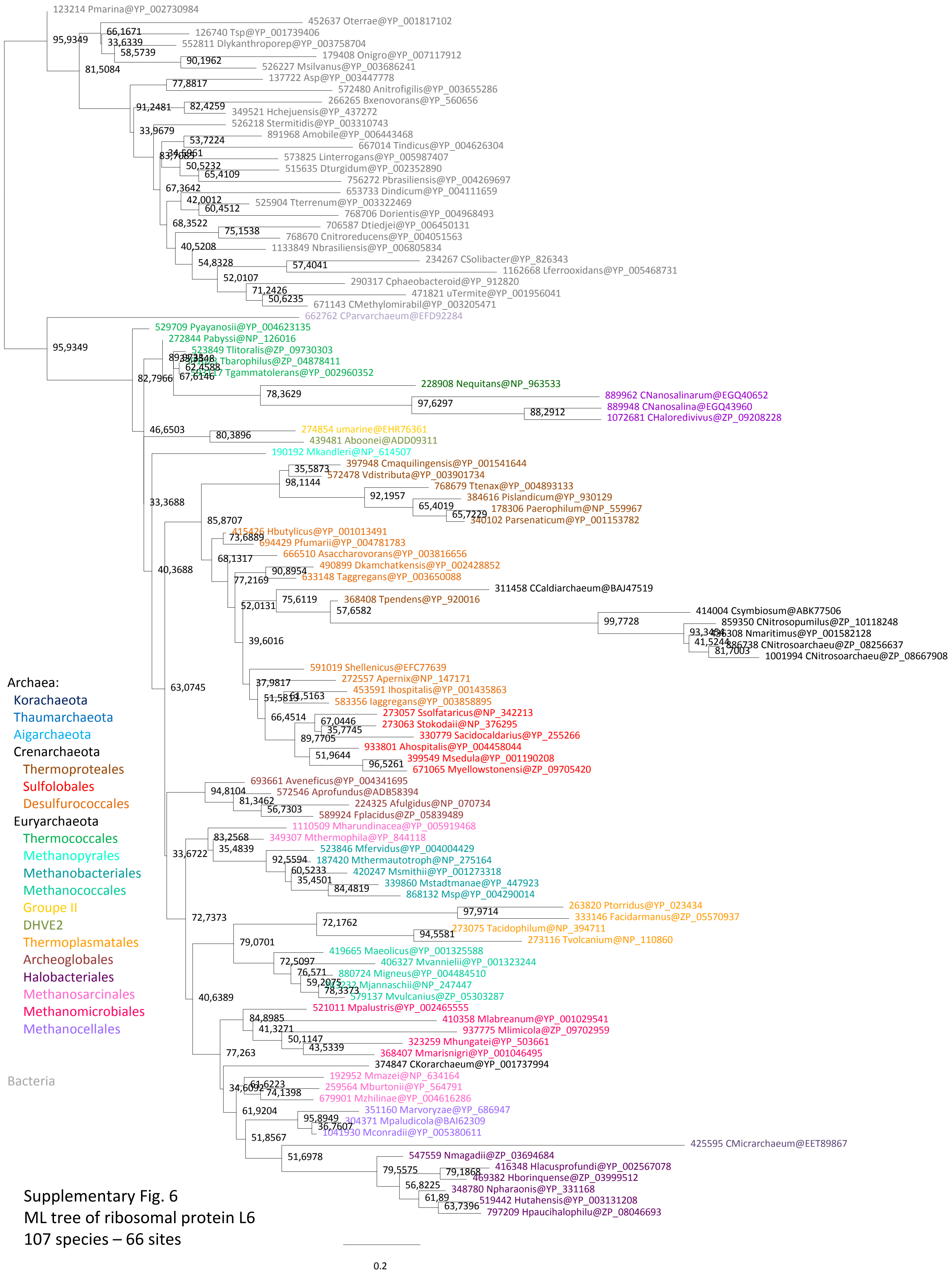


Supplementary Fig. 6
ML tree of ribosomal protein L6
107 species – 66 sites

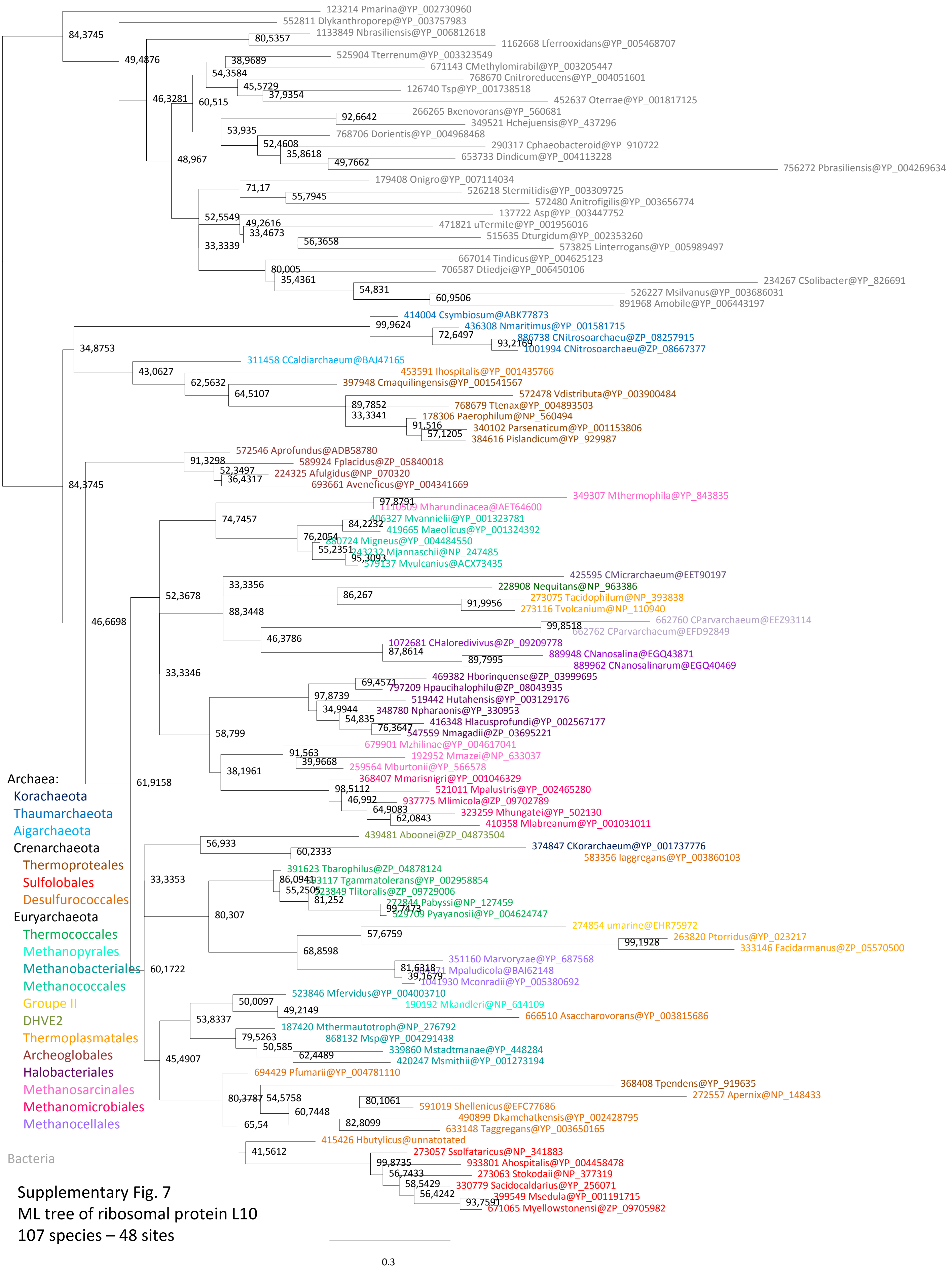


Supplementary Fig. 7
ML tree of ribosomal protein L10
107 species – 48 sites

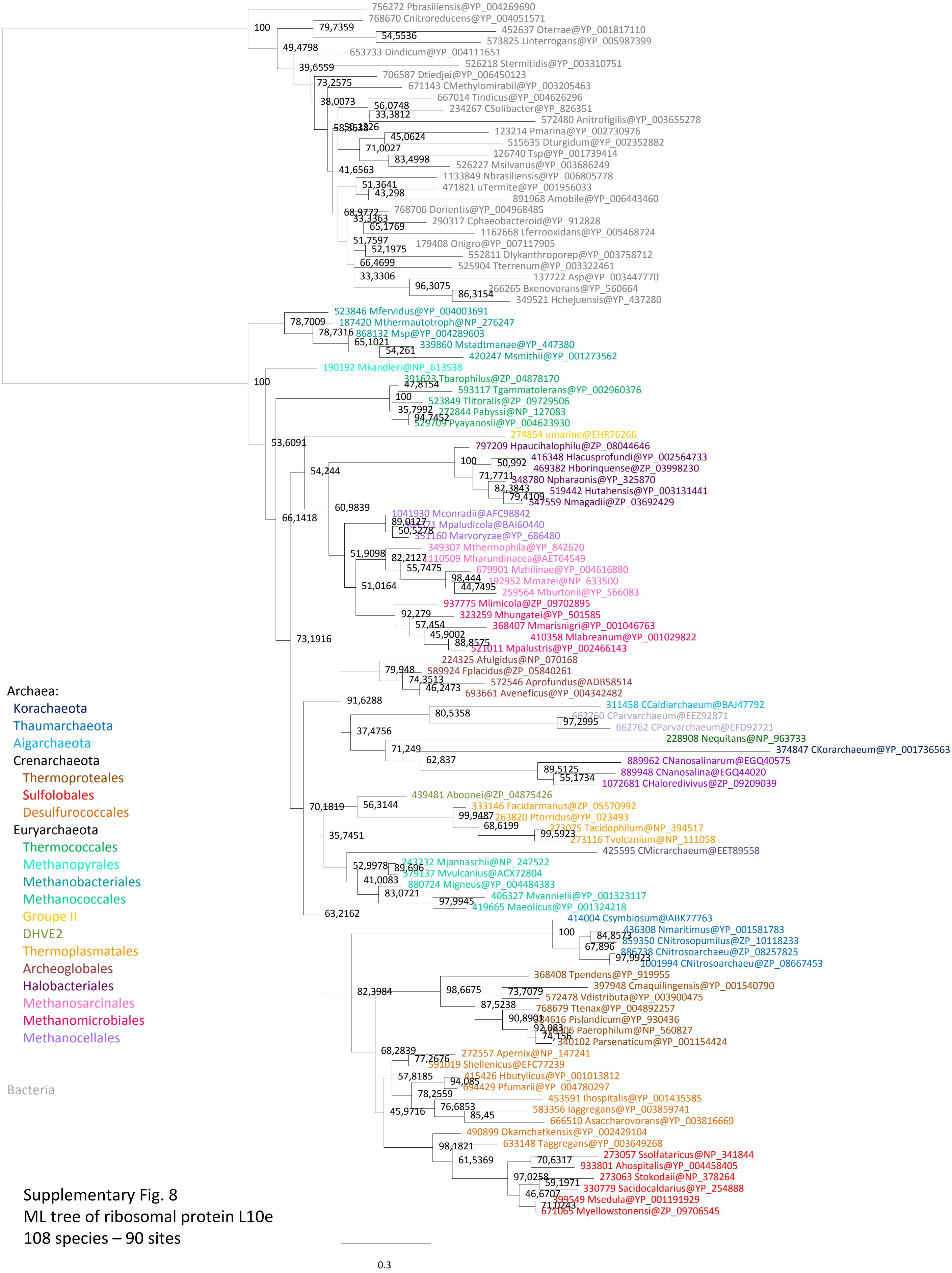


Supplementary Fig. 8
ML tree of ribosomal protein L10e
108 species – 90 sites

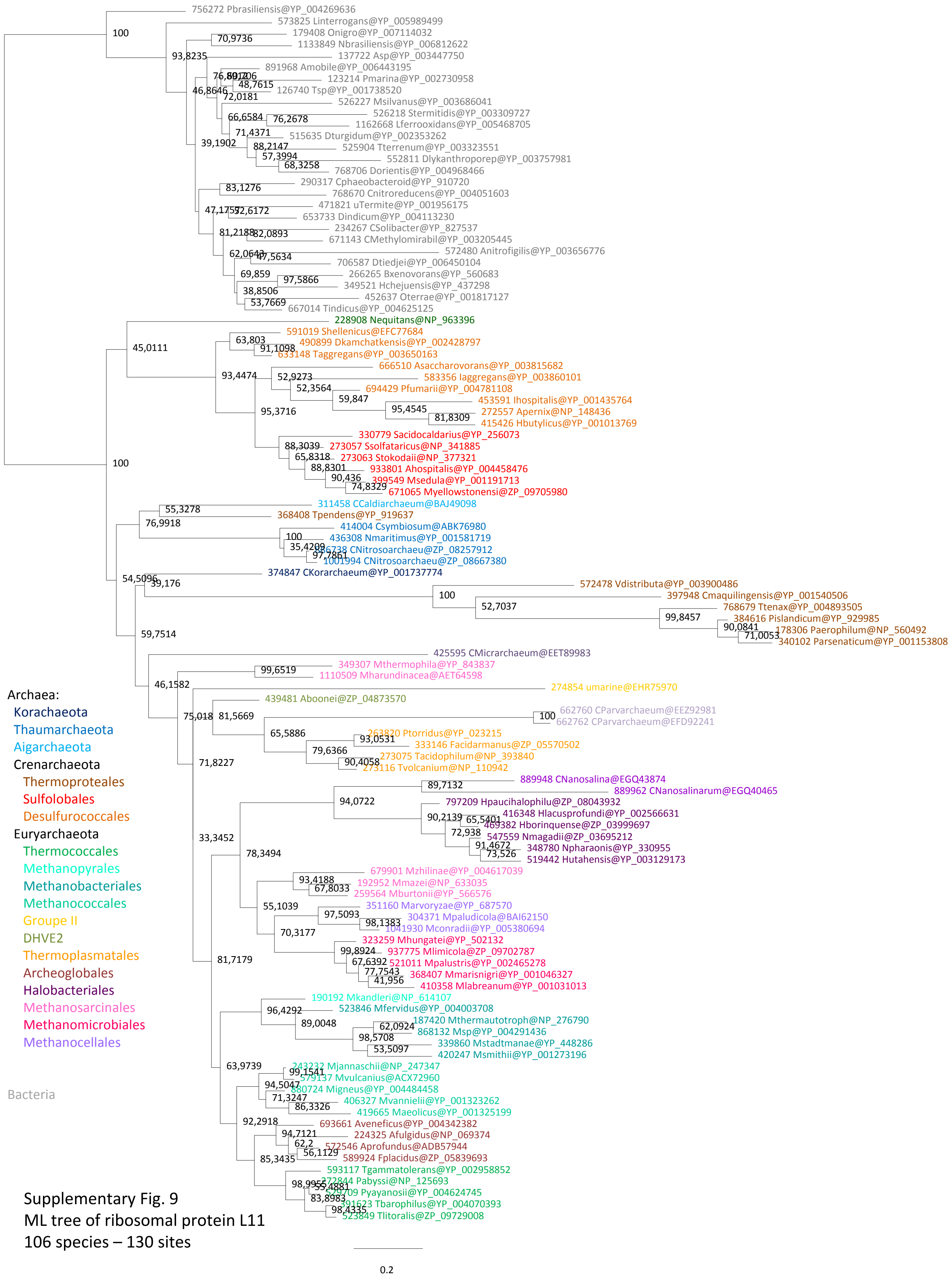


Supplementary Fig. 9
ML tree of ribosomal protein L11
106 species – 130 sites

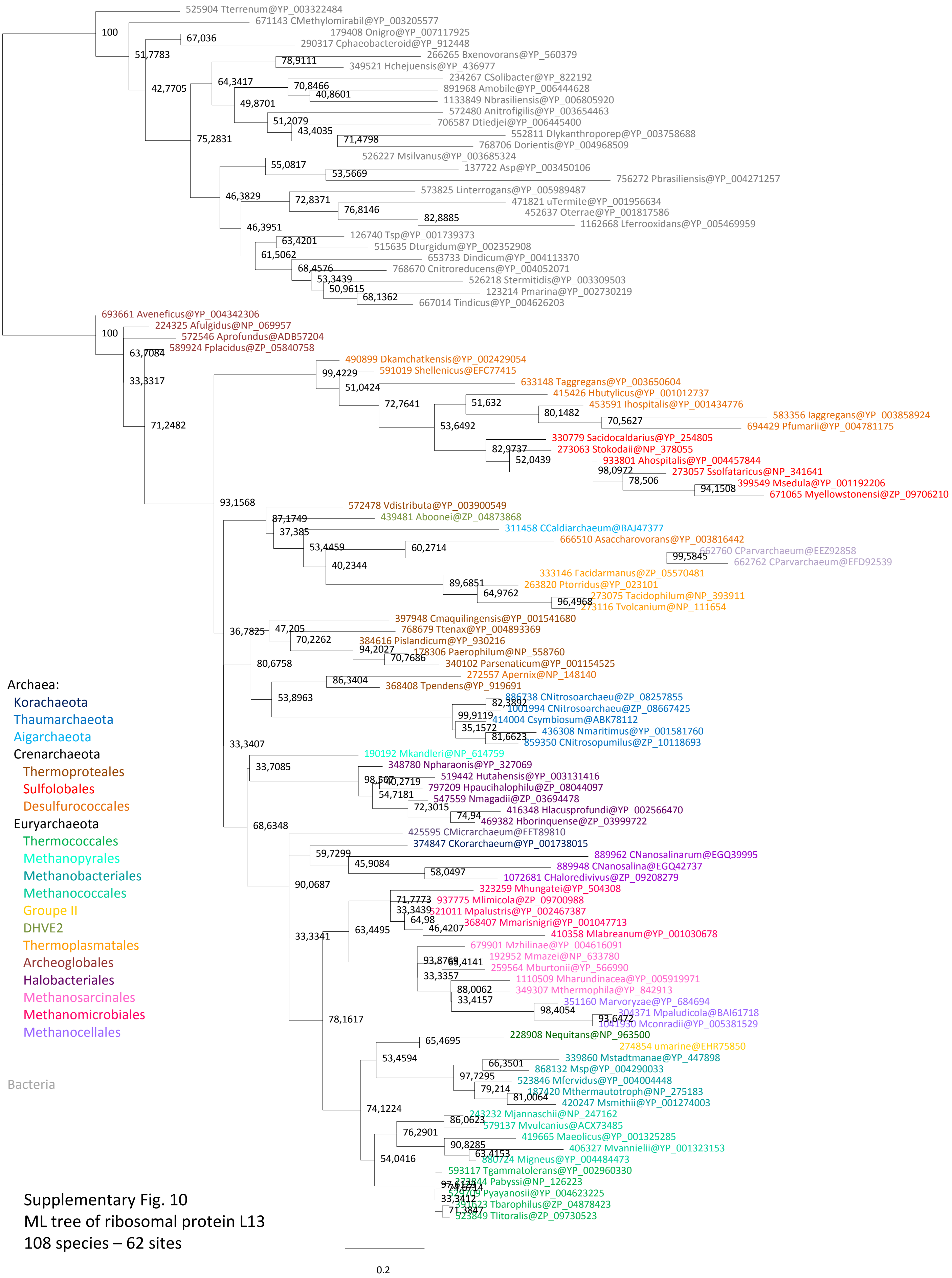


Supplementary Fig. 10
ML tree of ribosomal protein L13
108 species – 62 sites

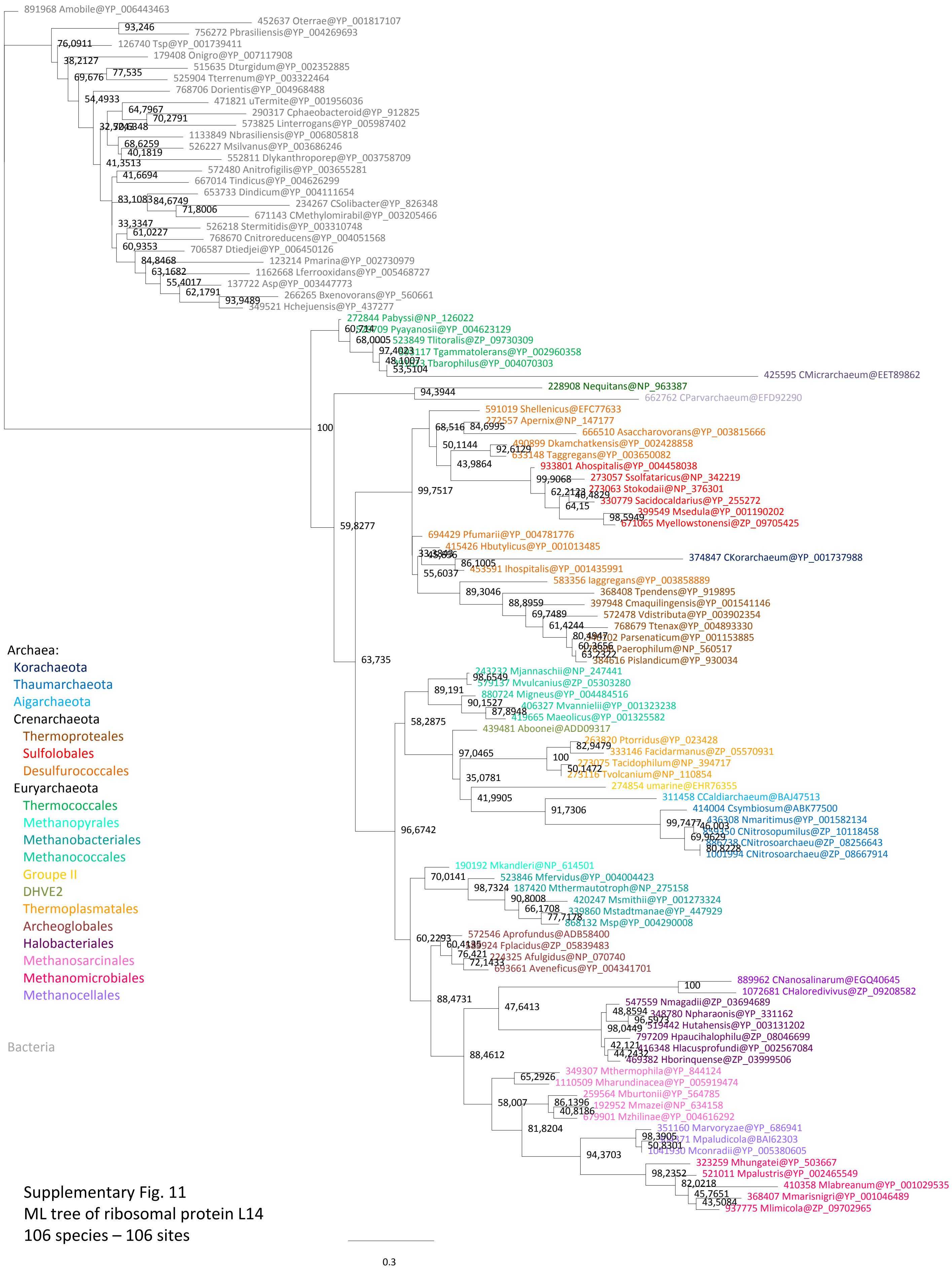


Supplementary Fig. 11
ML tree of ribosomal protein L14
106 species – 106 sites

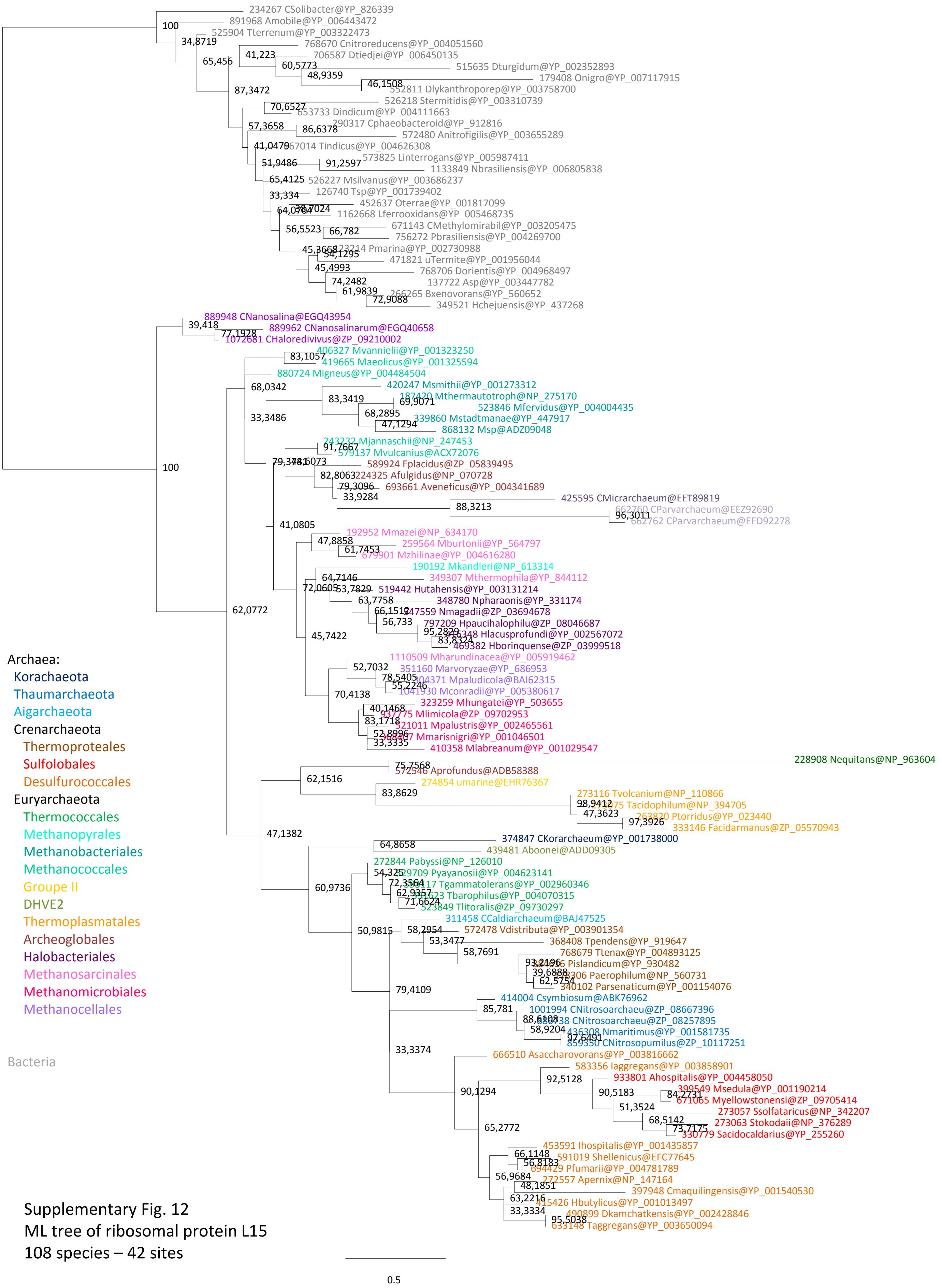


Supplementary Fig. 12
ML tree of ribosomal protein L15
108 species – 42 sites

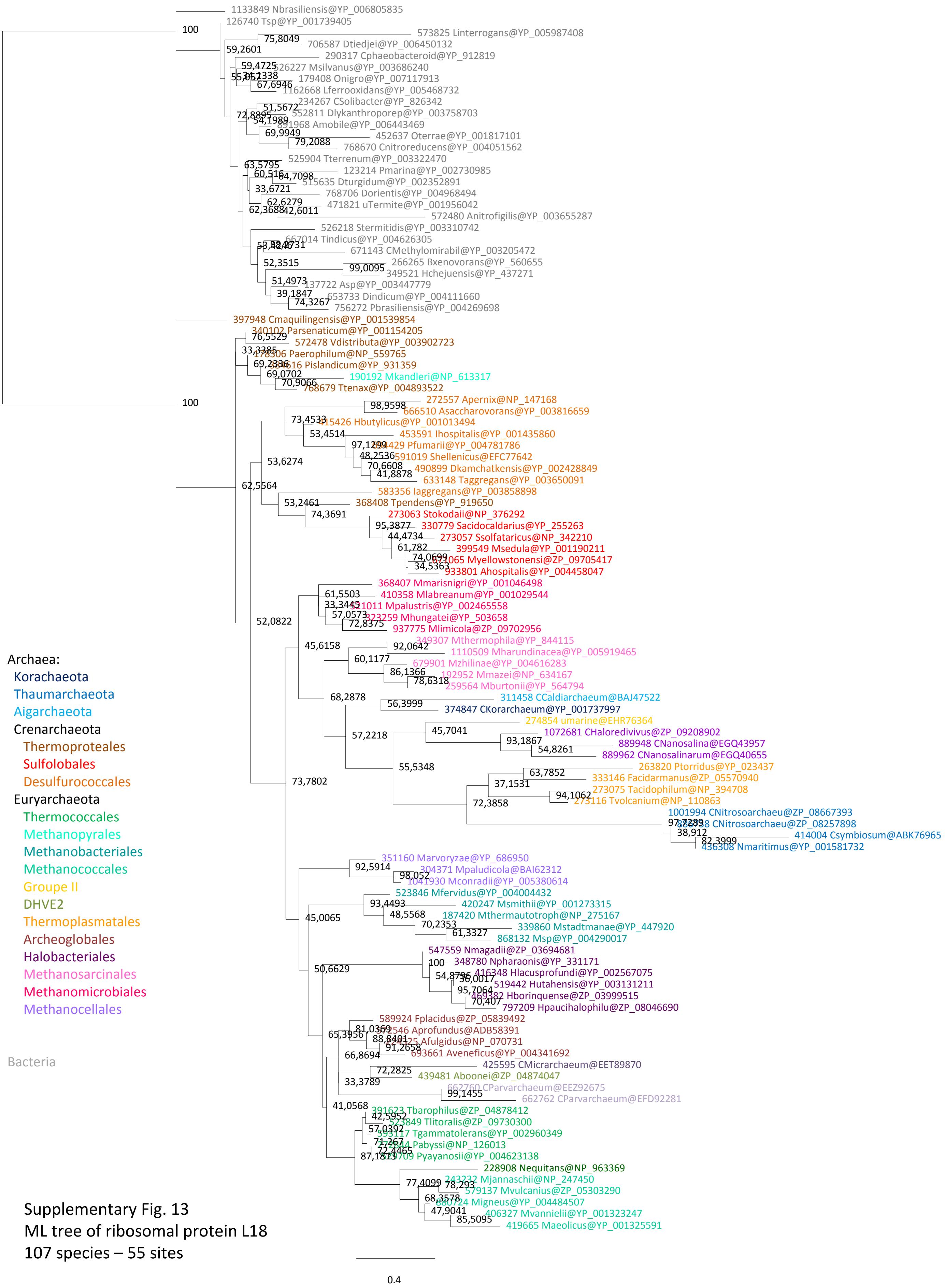


Supplementary Fig. 13
ML tree of ribosomal protein L18
107 species – 55 sites

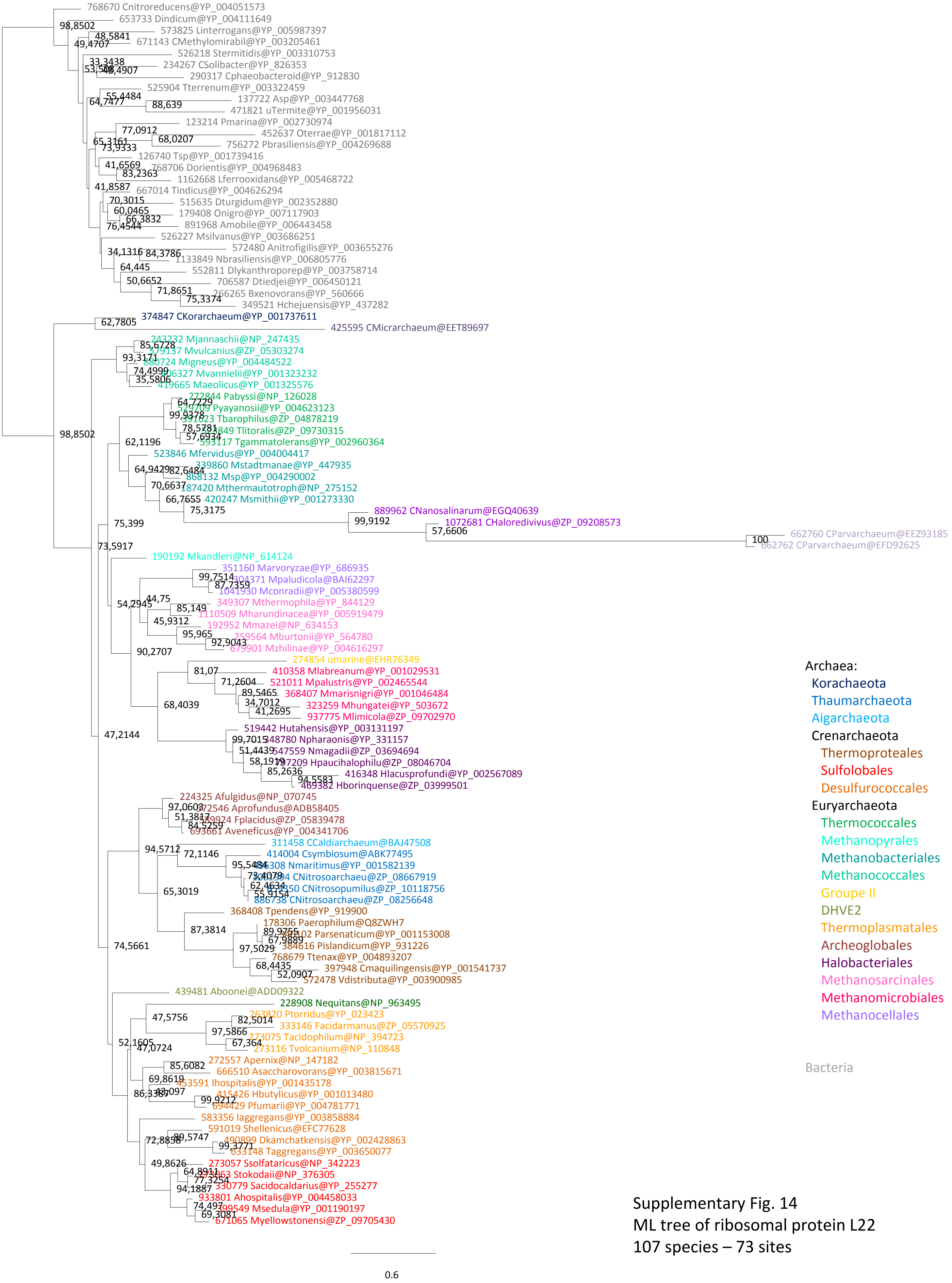


Supplementary Fig. 14
ML tree of ribosomal protein L22
107 species – 73 sites

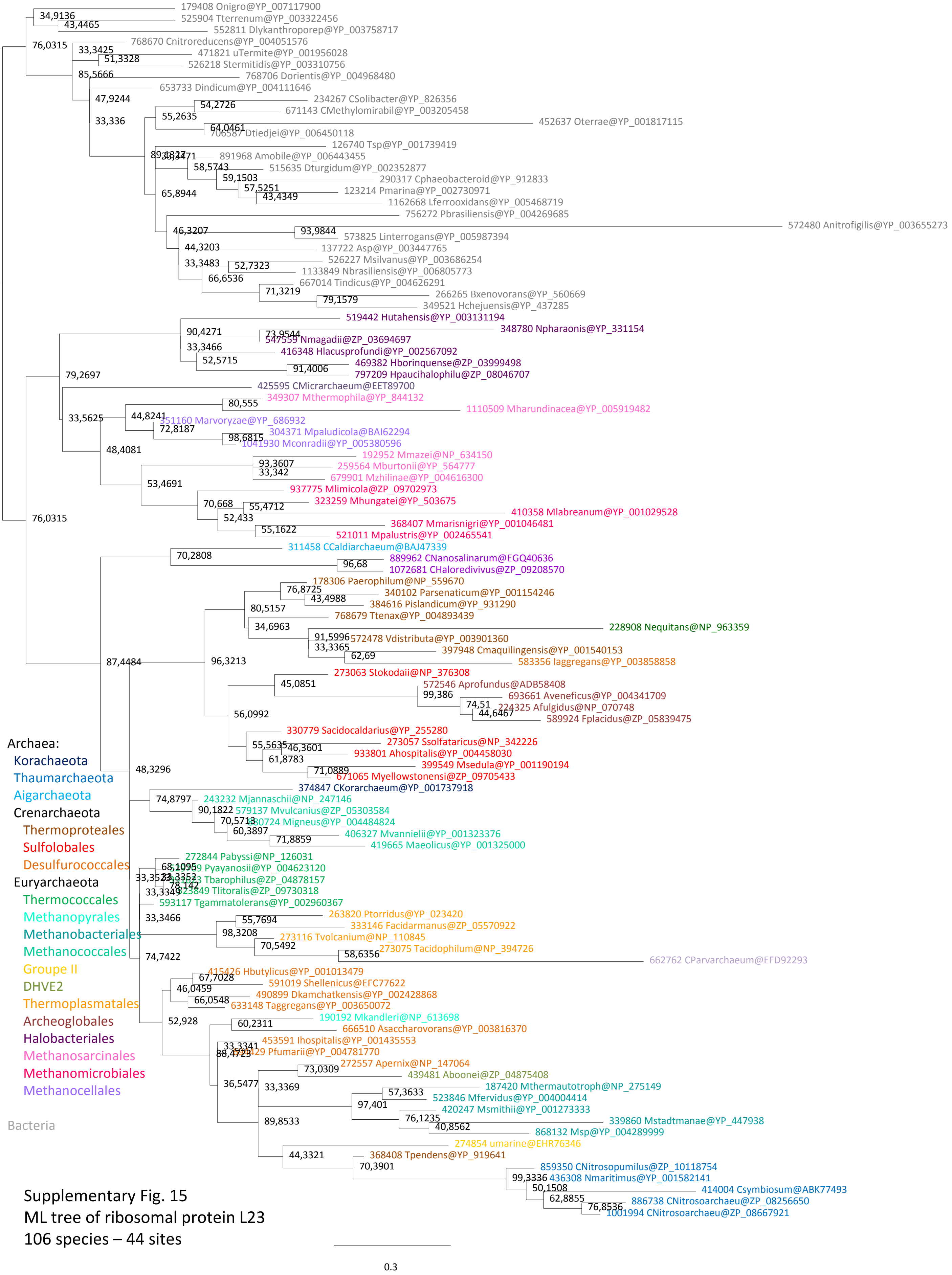


Supplementary Fig. 15
ML tree of ribosomal protein L23
106 species – 44 sites

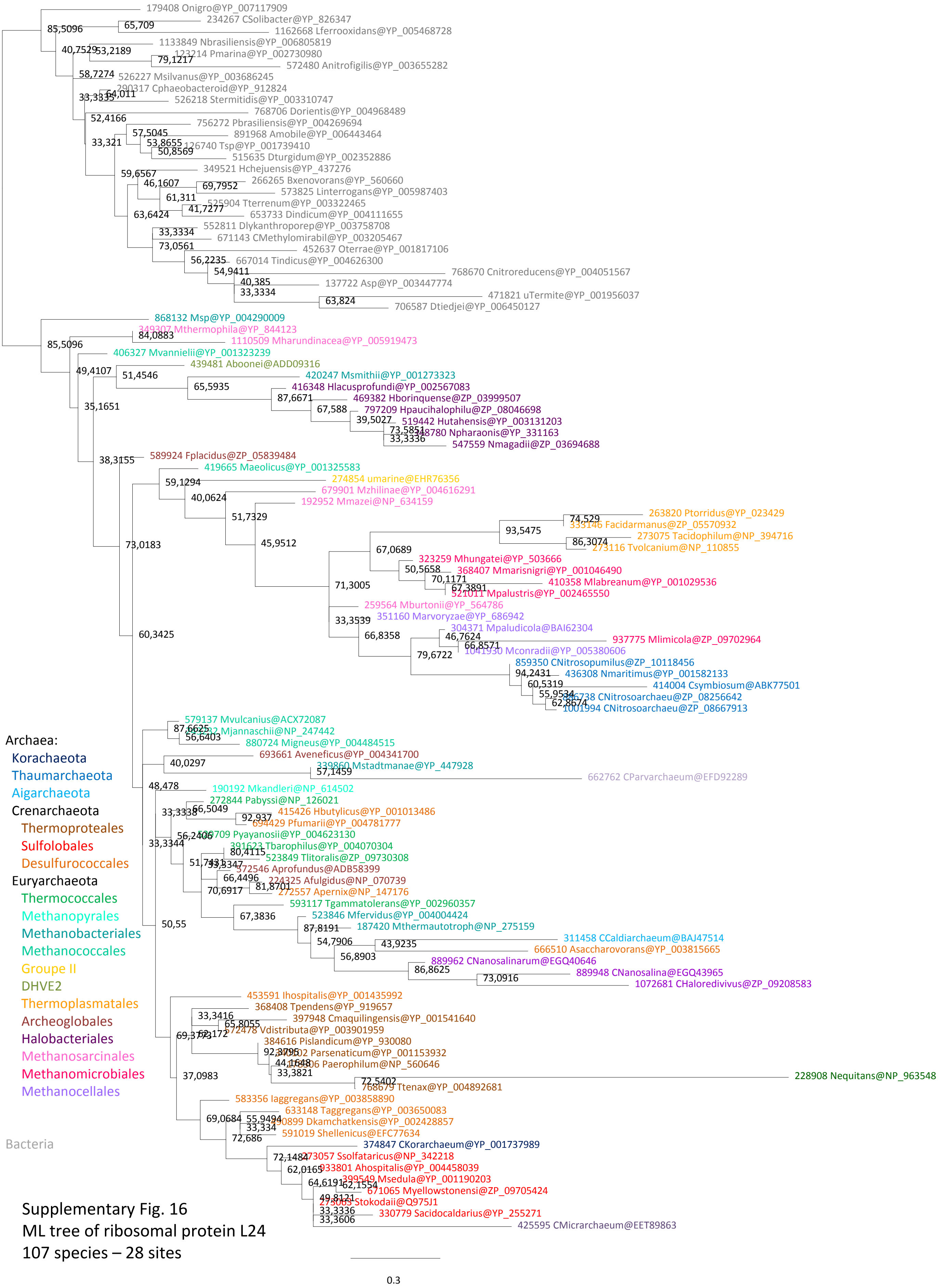


Supplementary Fig. 16
ML tree of ribosomal protein L24
107 species – 28 sites

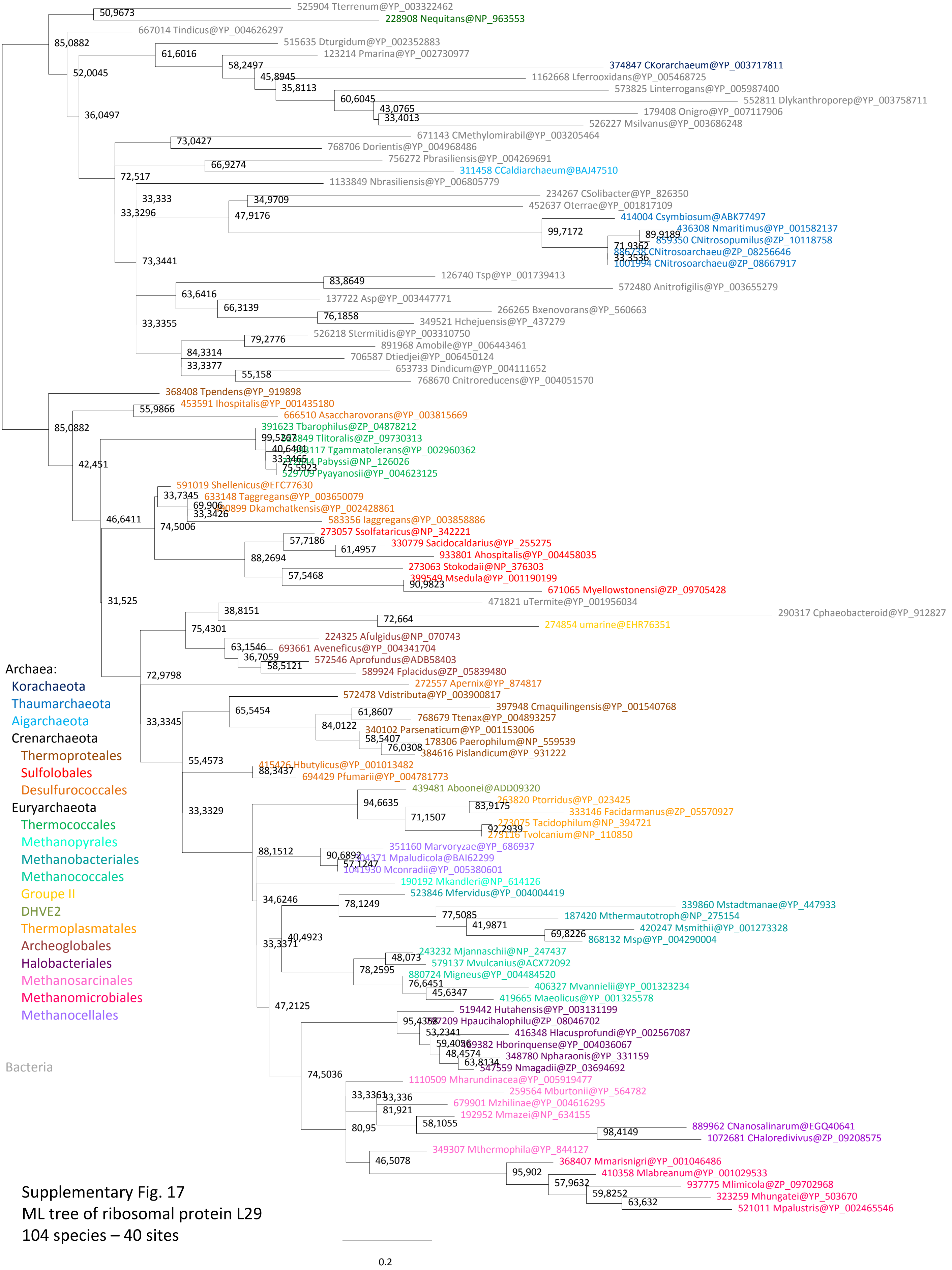


Supplementary Fig. 17
ML tree of ribosomal protein L29
104 species – 40 sites

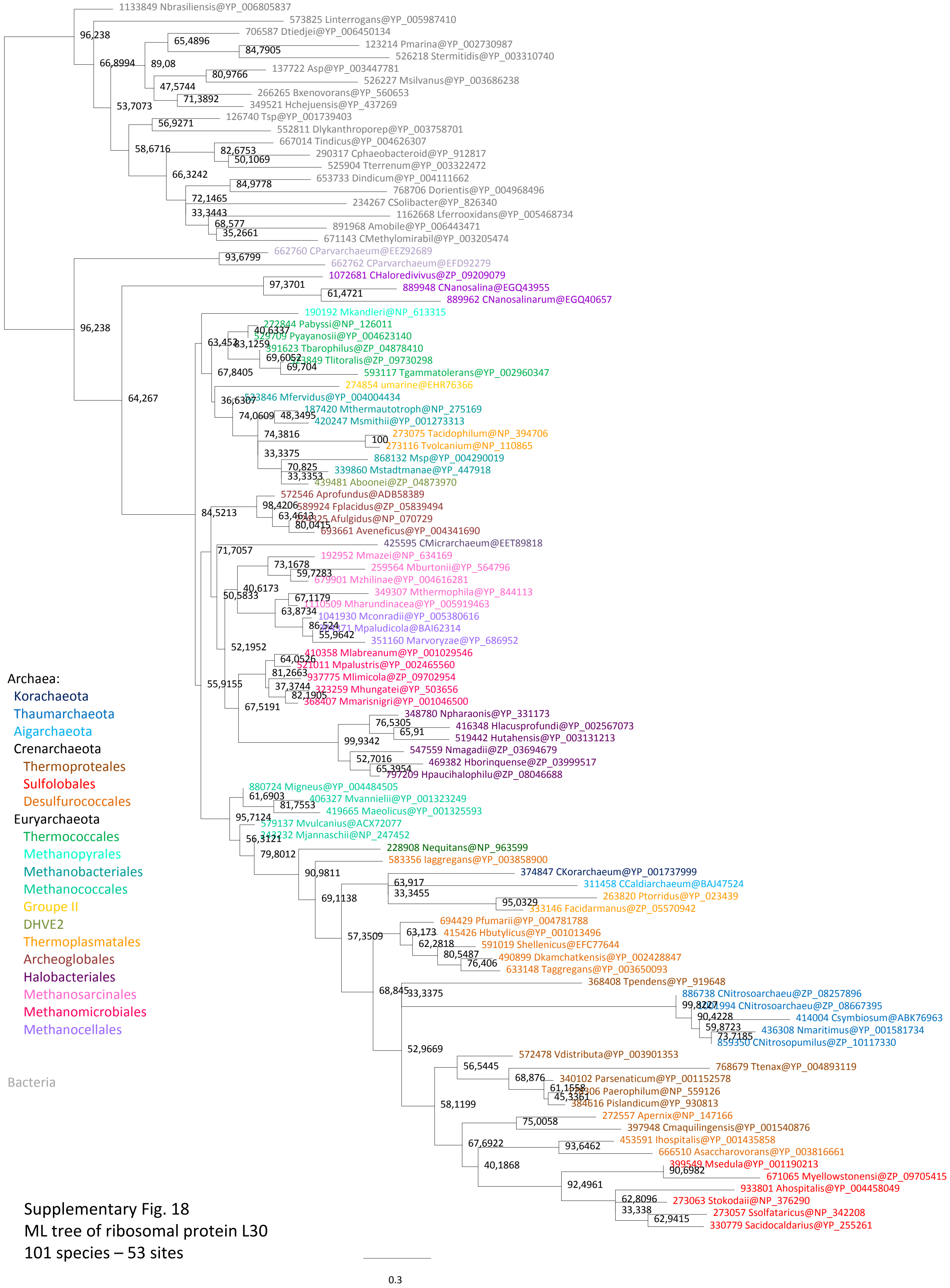


Supplementary Fig. 18
ML tree of ribosomal protein L30
101 species – 53 sites

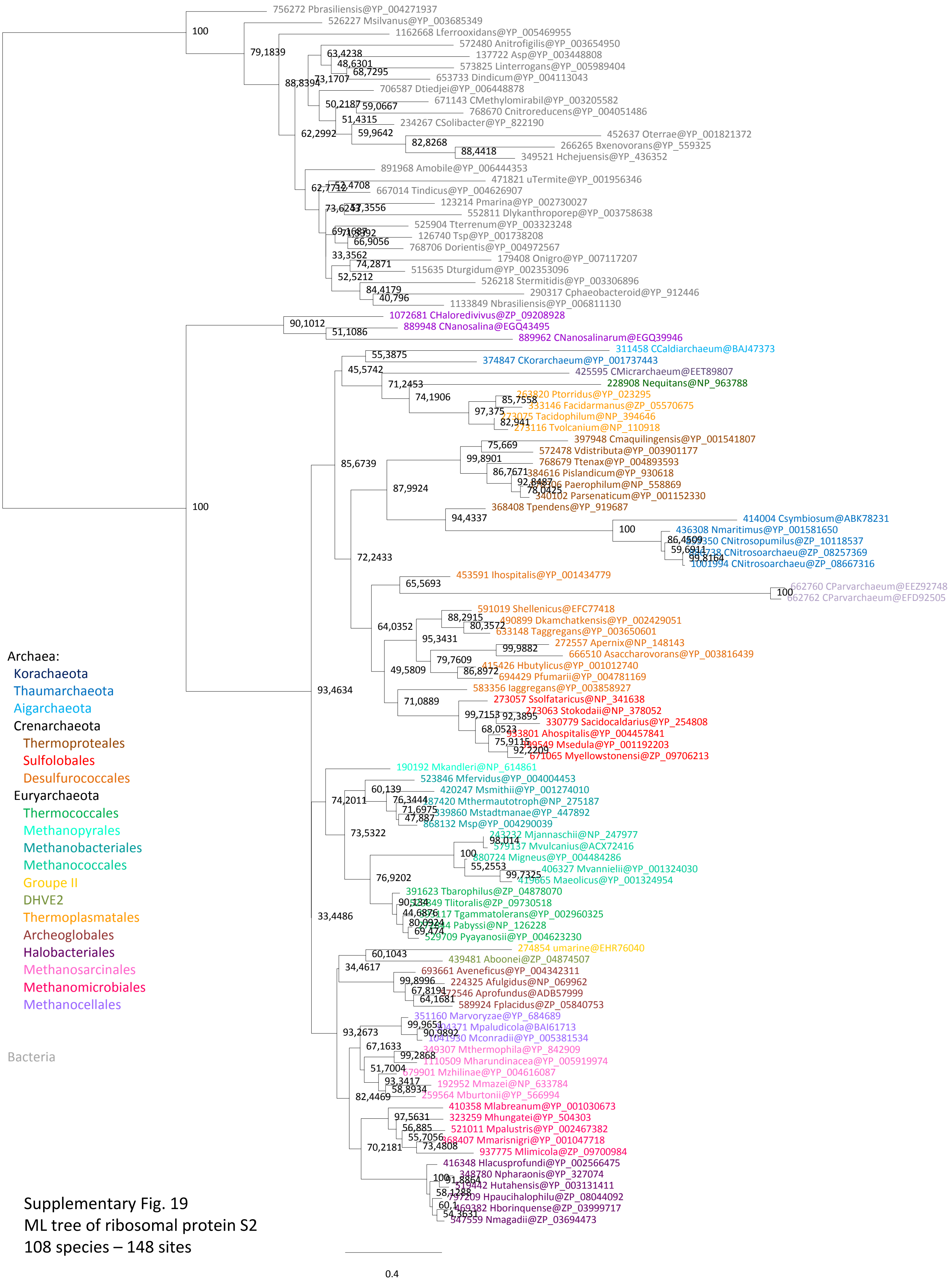


Supplementary Fig. 19
ML tree of ribosomal protein S2
108 species – 148 sites

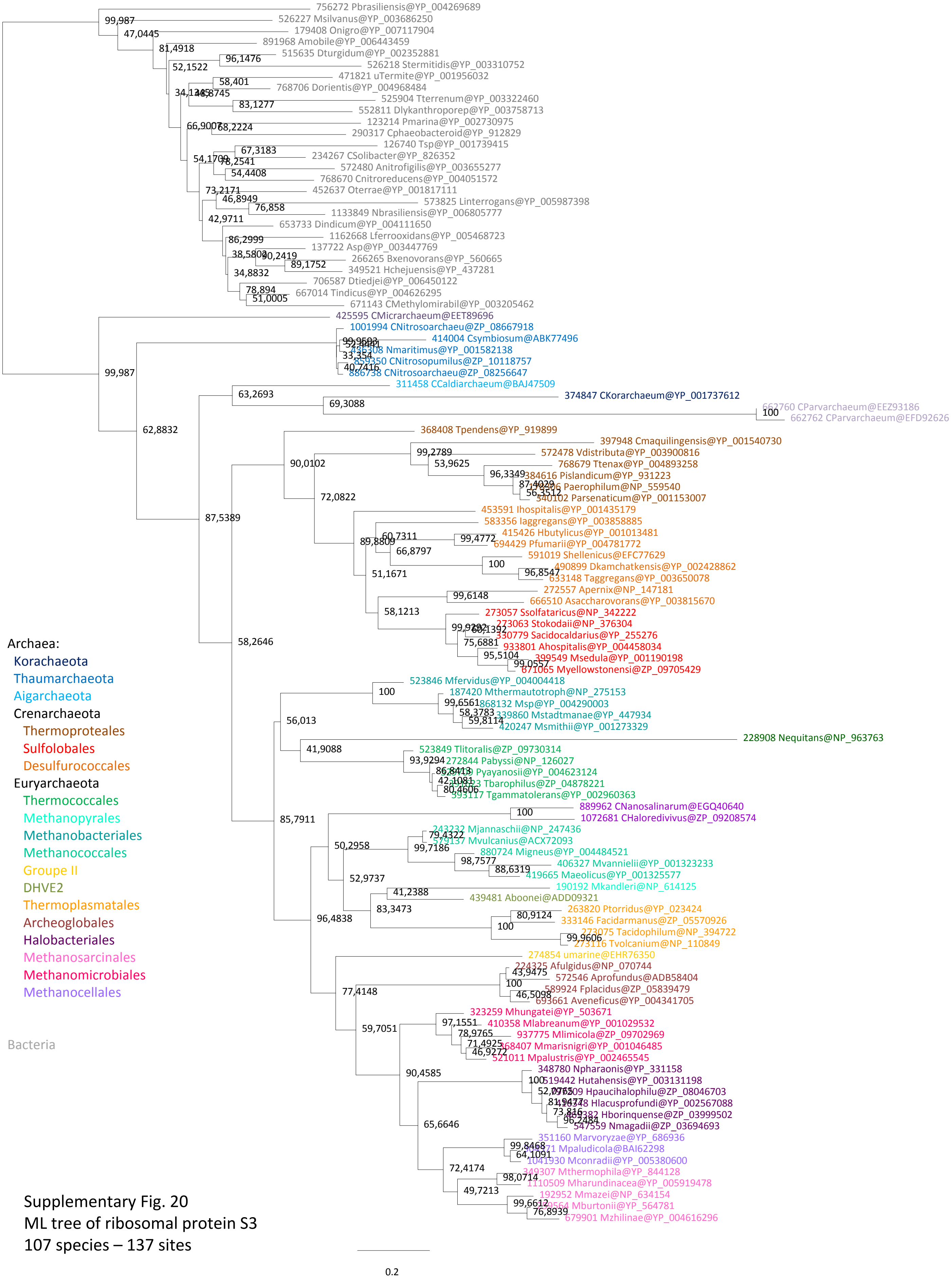


Supplementary Fig. 20
ML tree of ribosomal protein S3
107 species – 137 sites

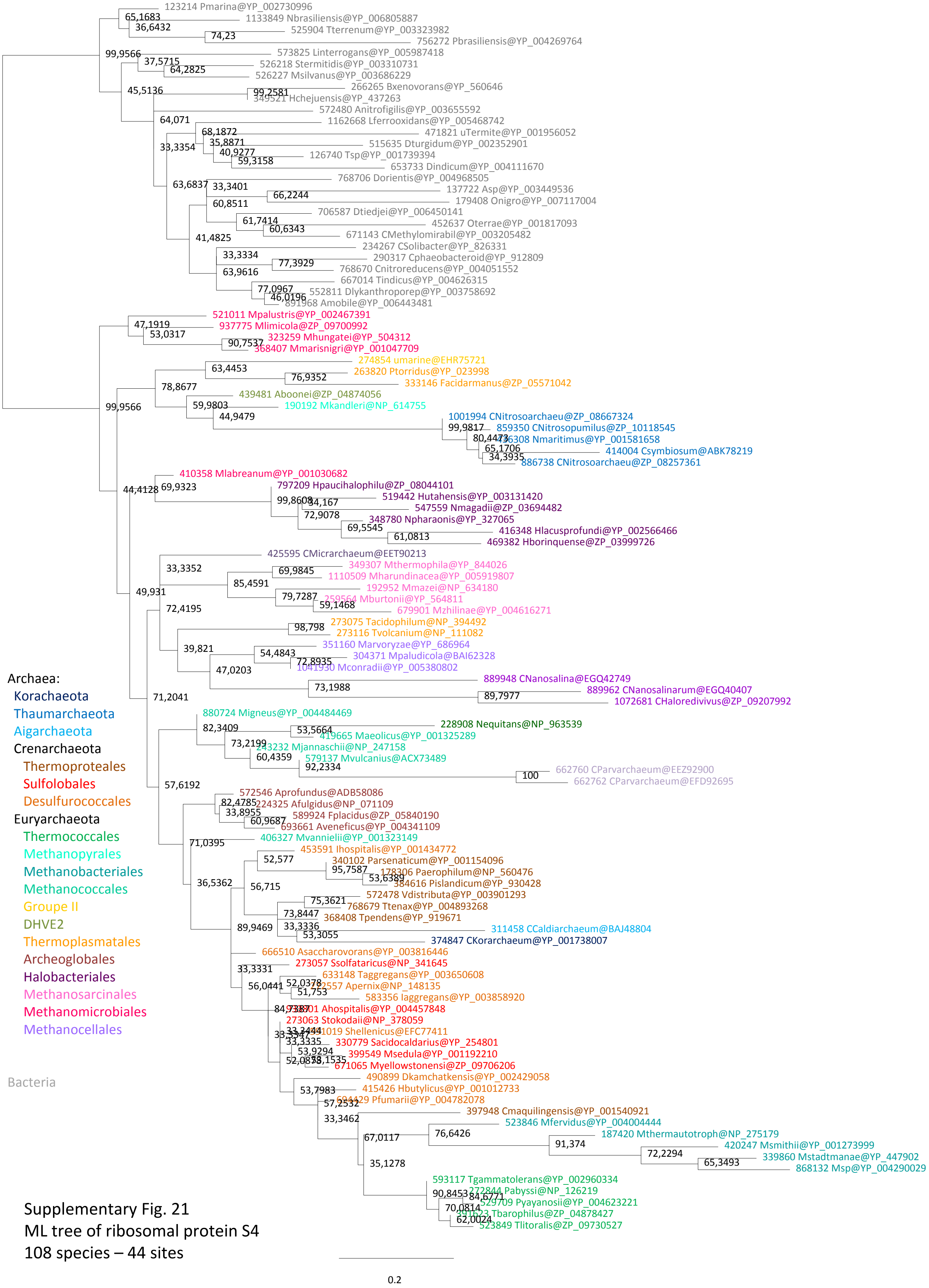


Supplementary Fig. 21
ML tree of ribosomal protein S4
108 species – 44 sites

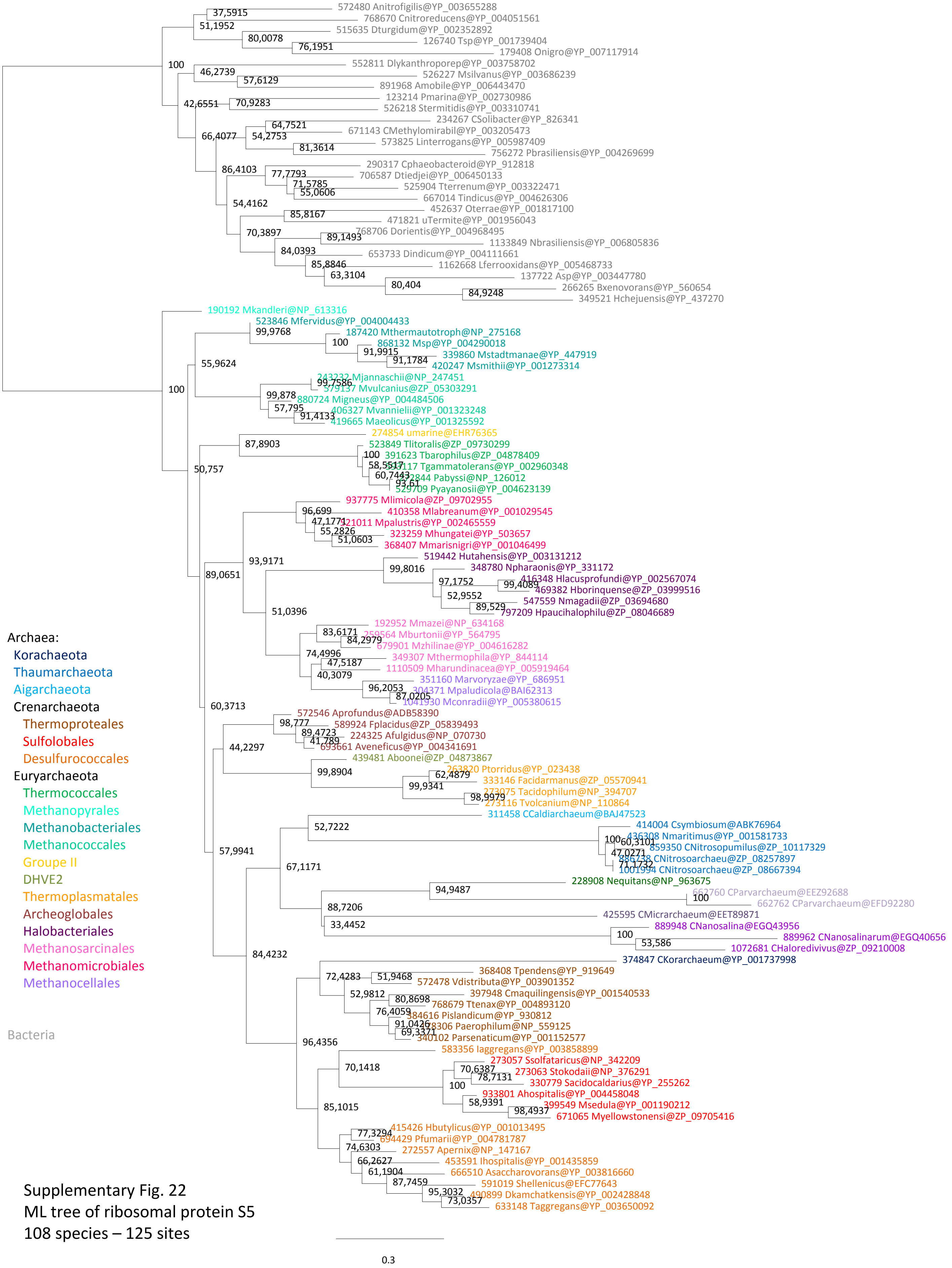


Supplementary Fig. 22
ML tree of ribosomal protein S5
108 species – 125 sites

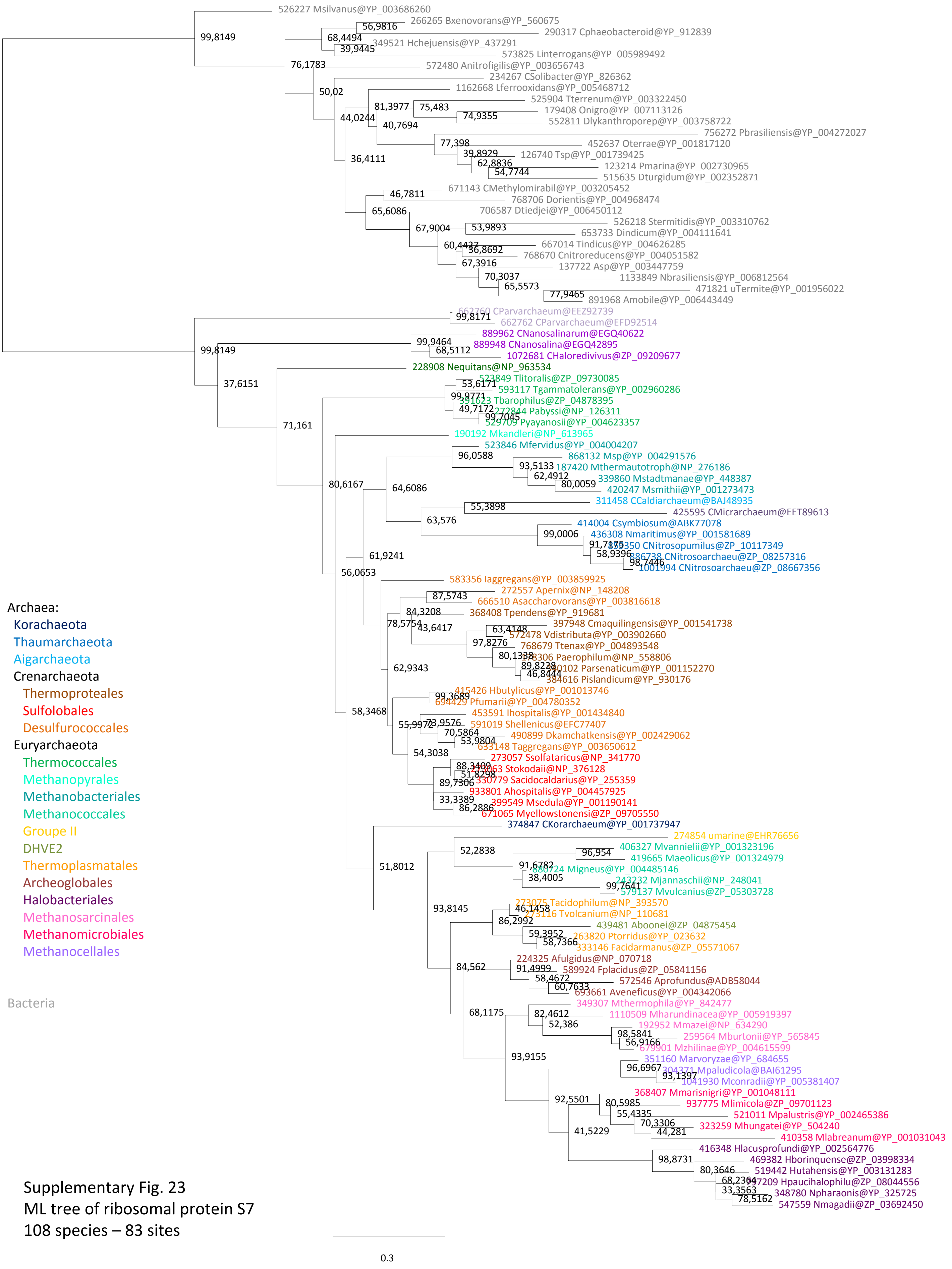


Supplementary Fig. 23
ML tree of ribosomal protein S7
108 species – 83 sites

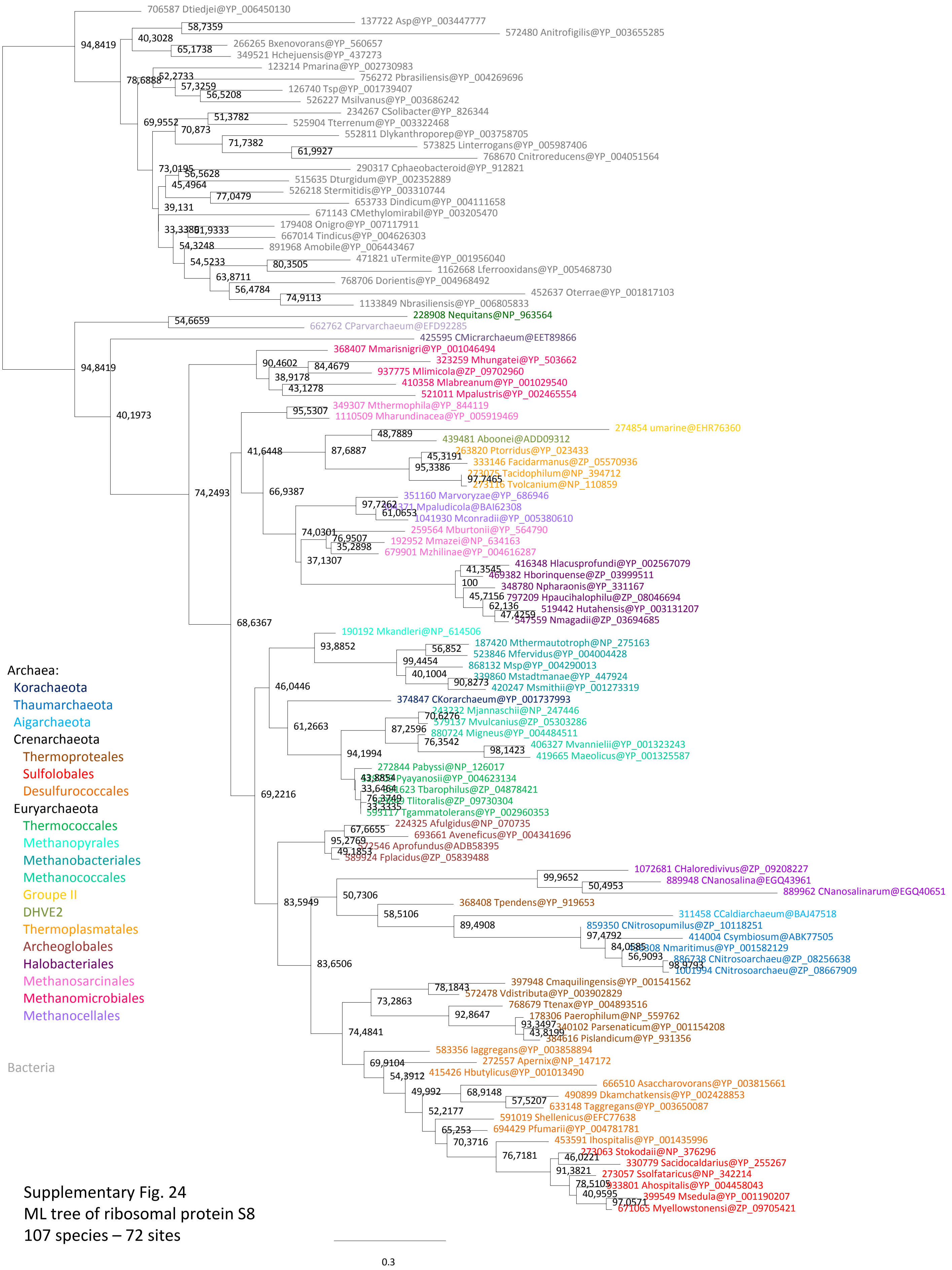


Supplementary Fig. 24
ML tree of ribosomal protein S8
107 species – 72 sites

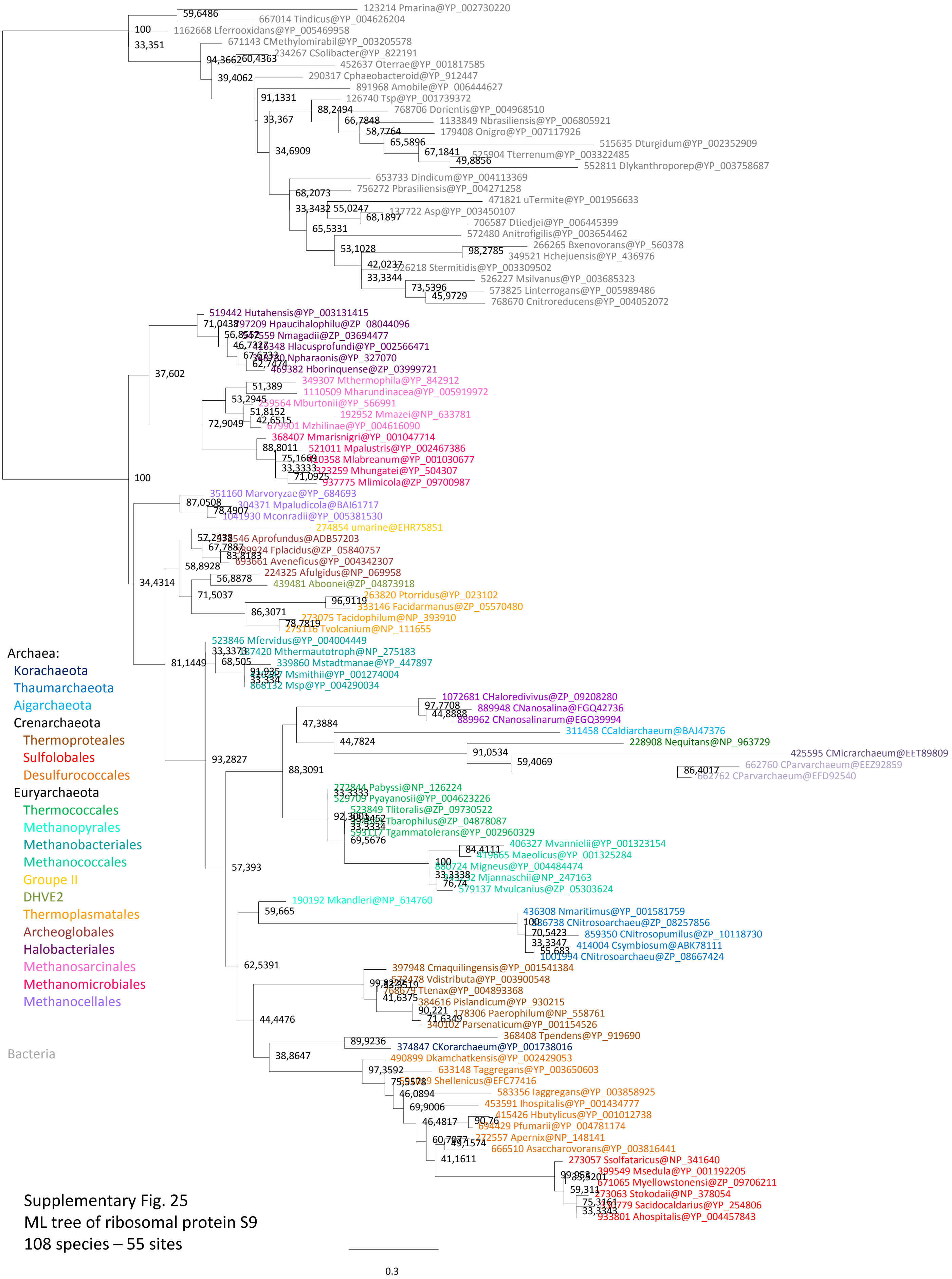


Supplementary Fig. 25
ML tree of ribosomal protein S9
108 species – 55 sites

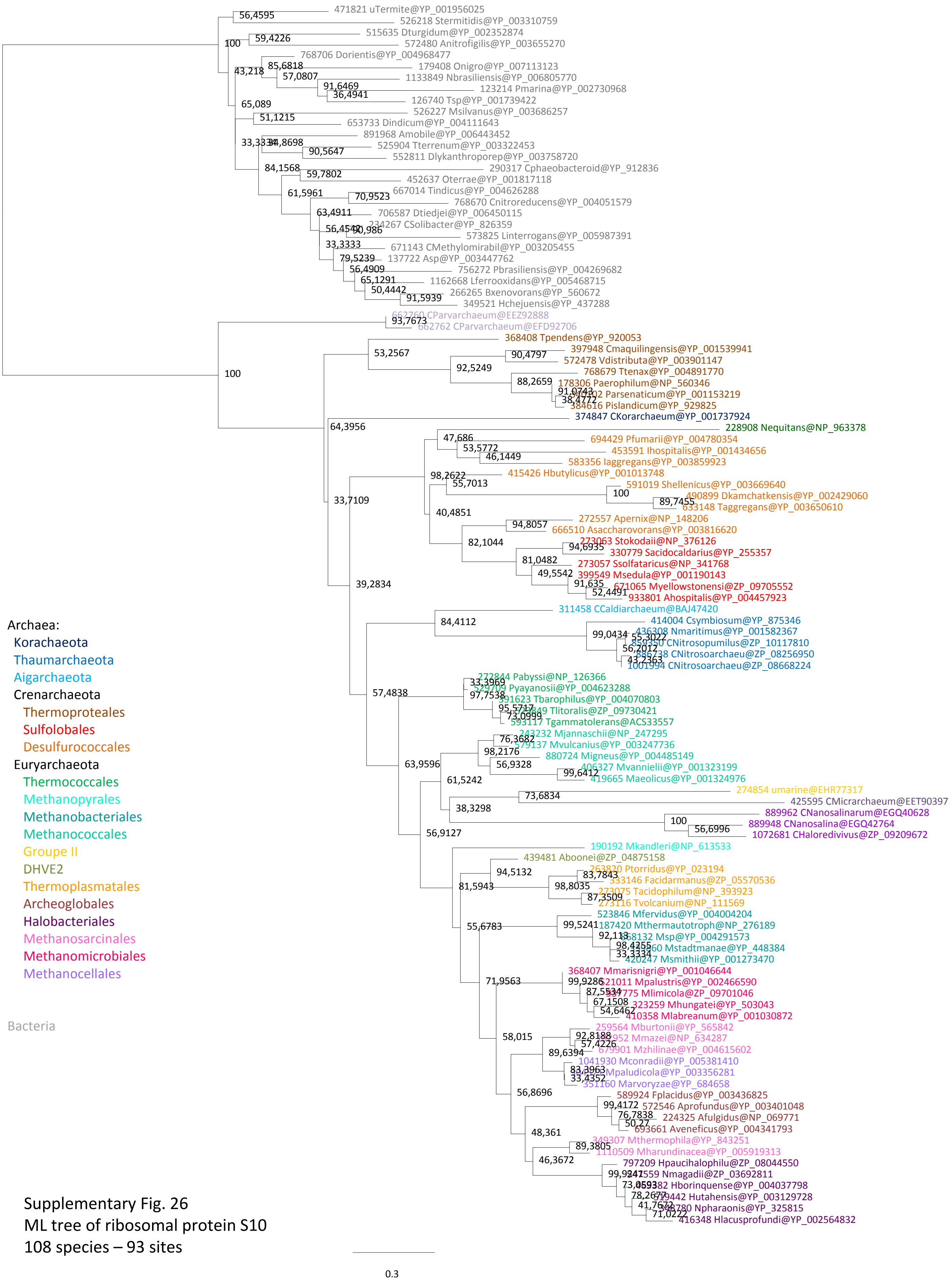

Supplementary Fig. 26
ML tree of ribosomal protein S10
108 species – 93 sites

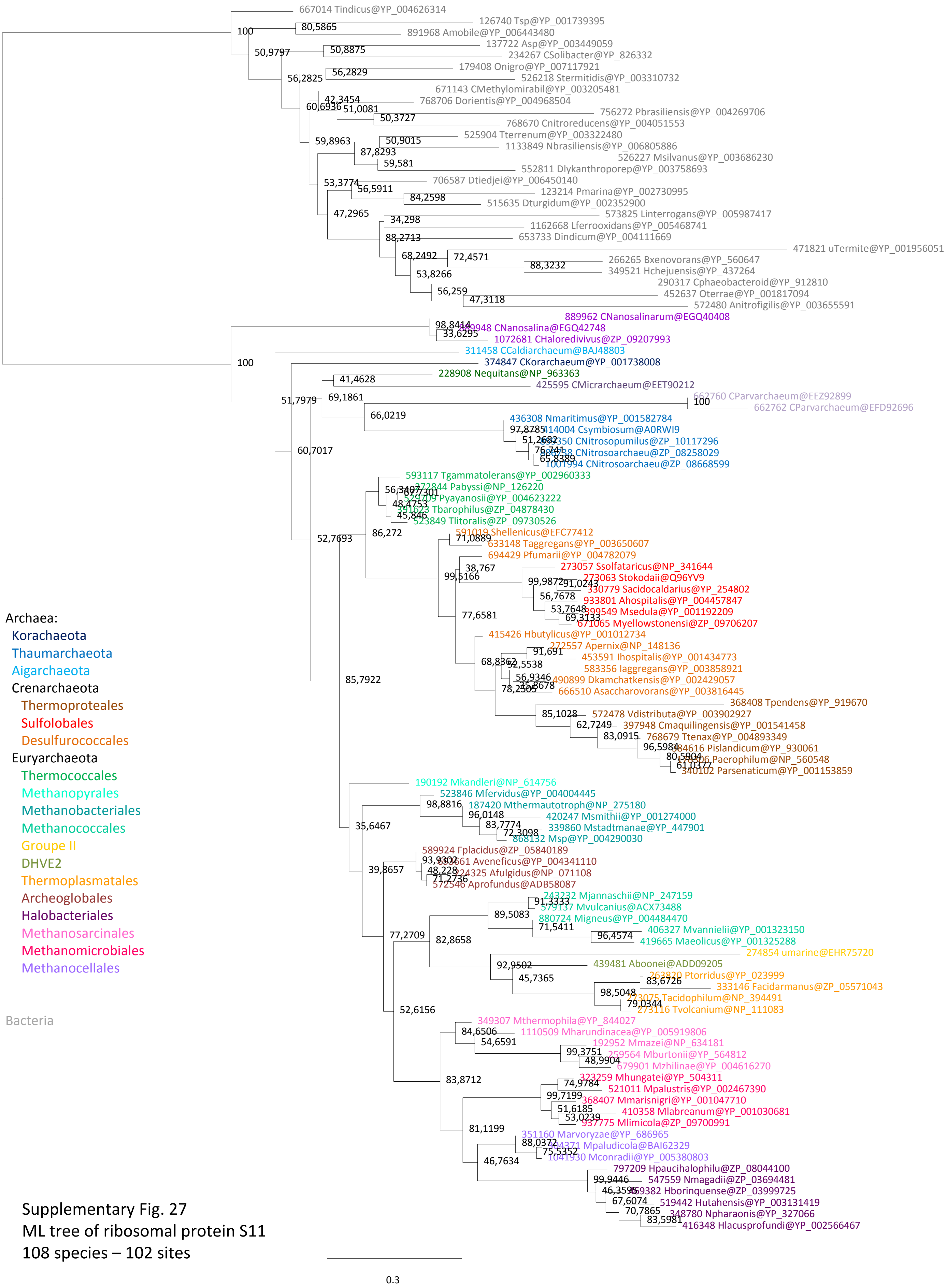


Supplementary Fig. 27
ML tree of ribosomal protein S11
108 species – 102 sites

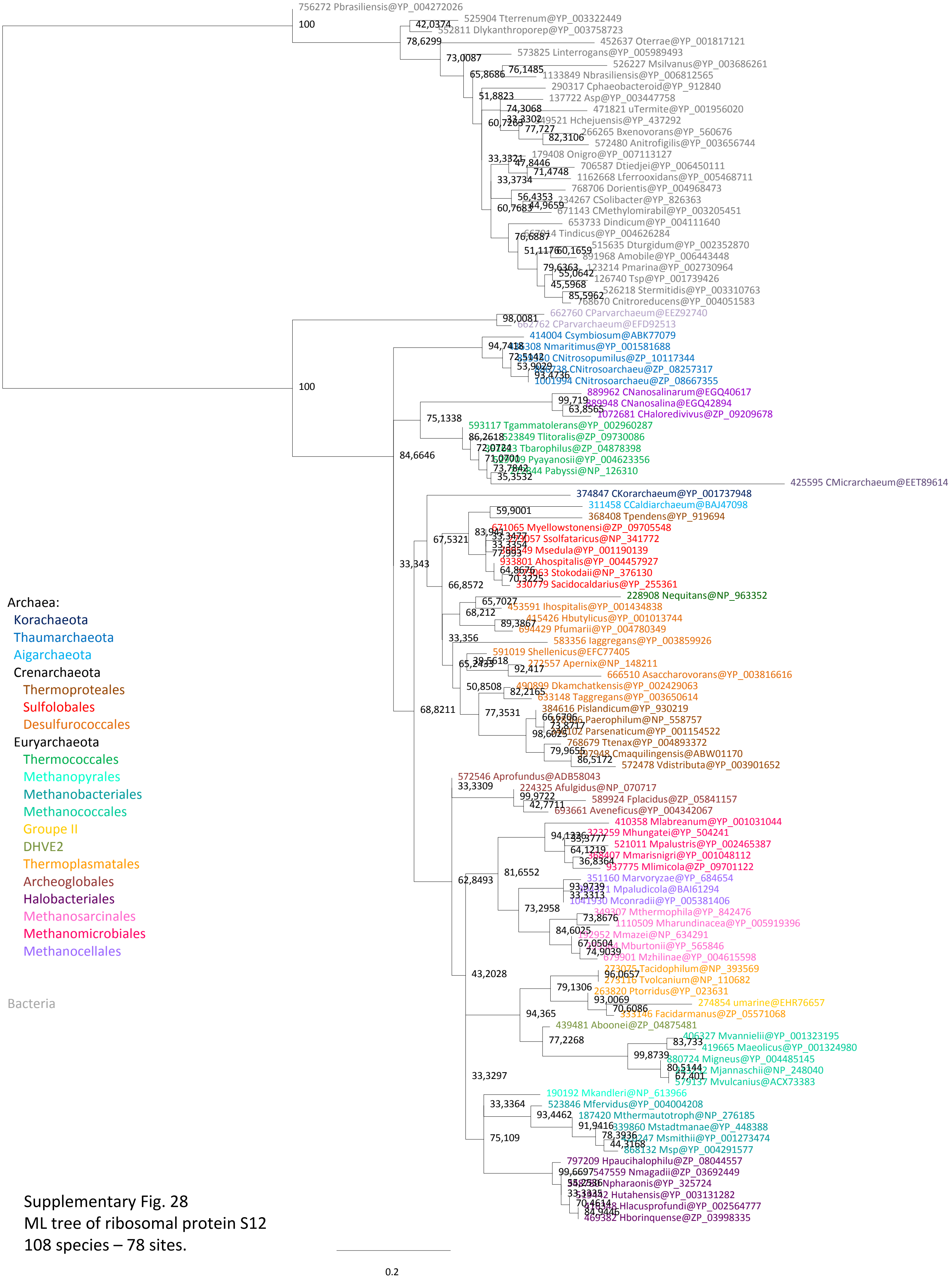


Supplementary Fig. 28
ML tree of ribosomal protein S12
108 species – 78 sites.

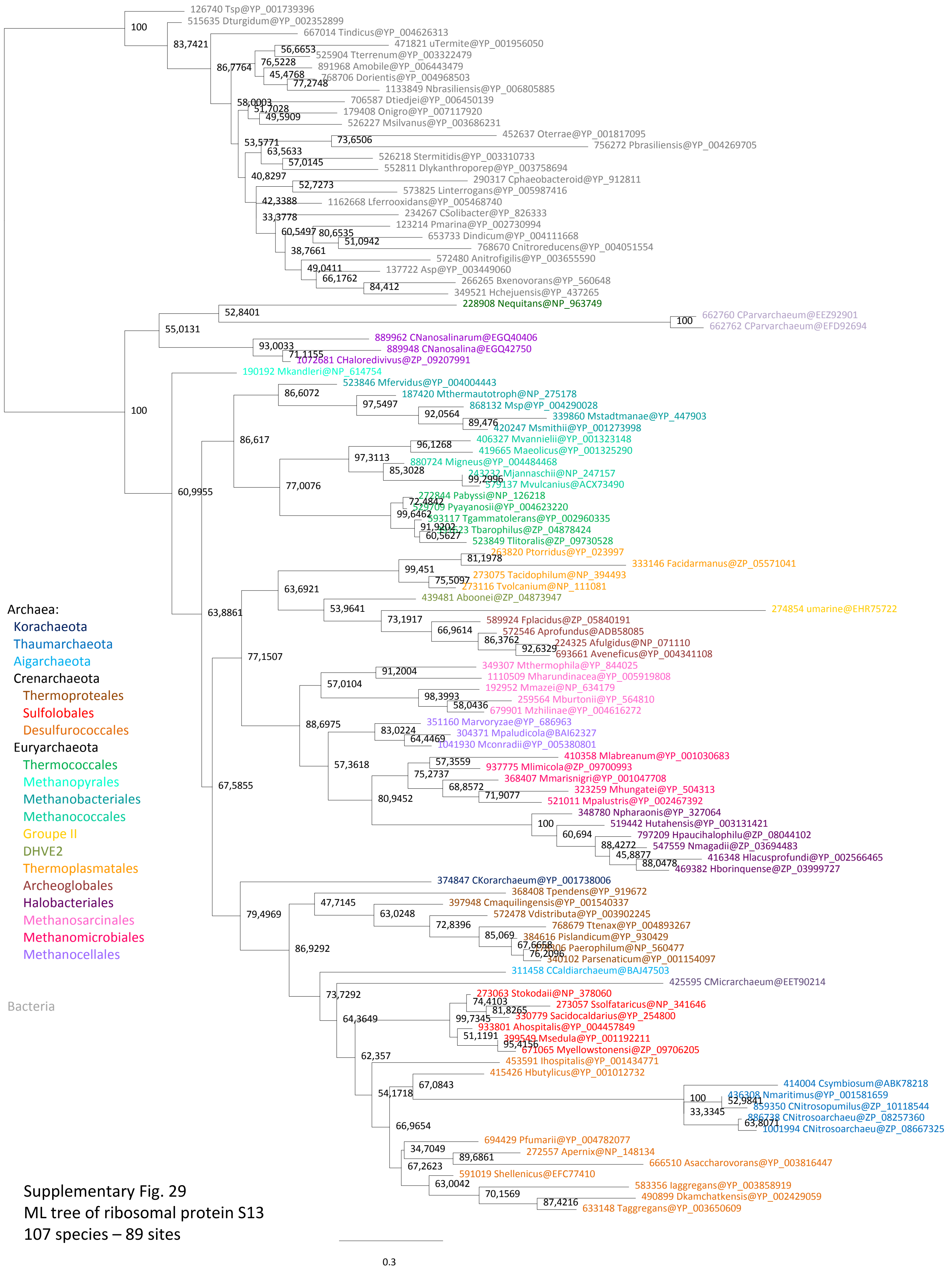


Supplementary Fig. 29
ML tree of ribosomal protein S13
107 species – 89 sites

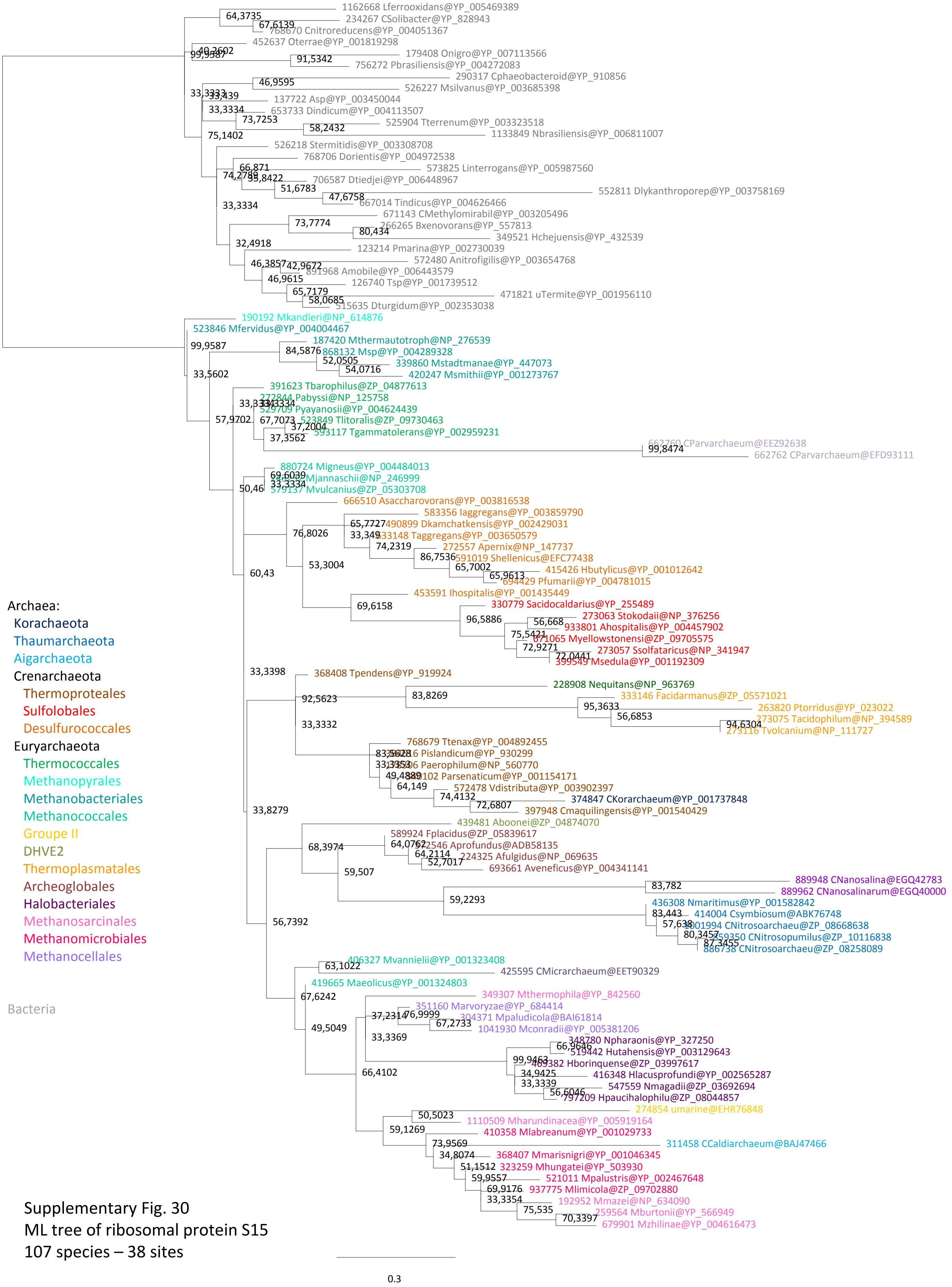


Supplementary Fig. 30
ML tree of ribosomal protein S15
107 species – 38 sites

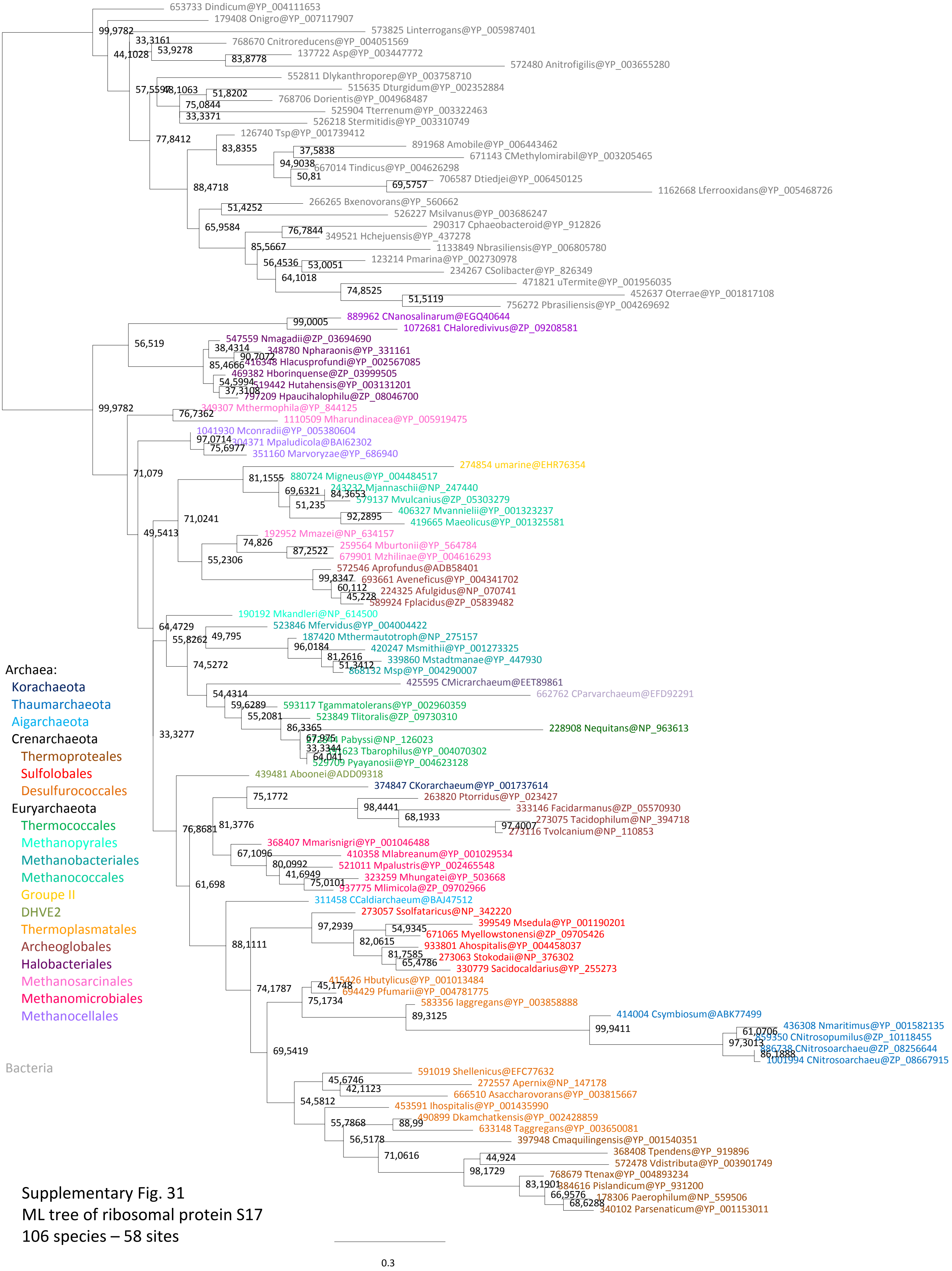


Supplementary Fig. 31
ML tree of ribosomal protein S17
106 species – 58 sites

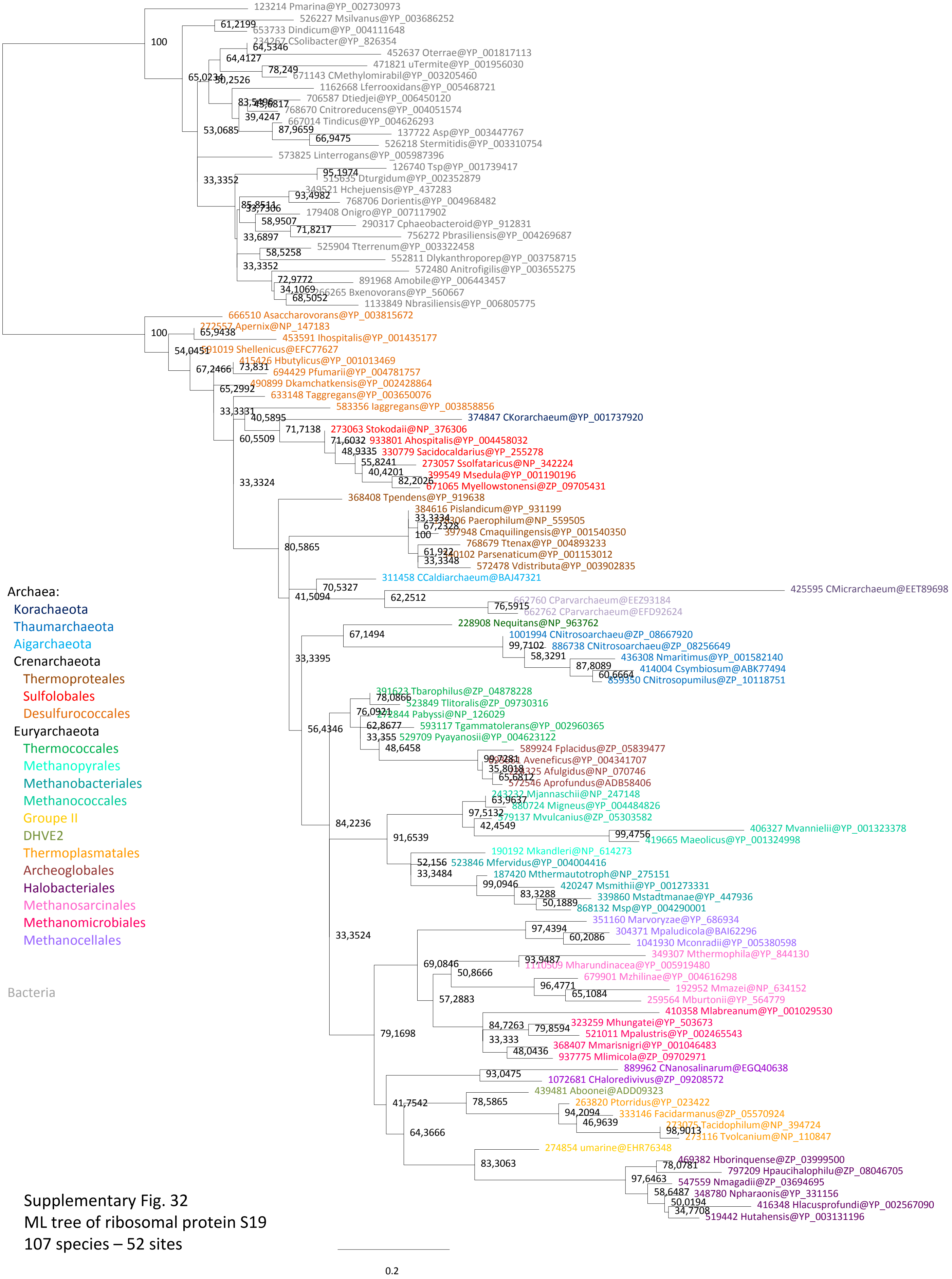


Supplementary Fig. 32
ML tree of ribosomal protein S19
107 species – 52 sites

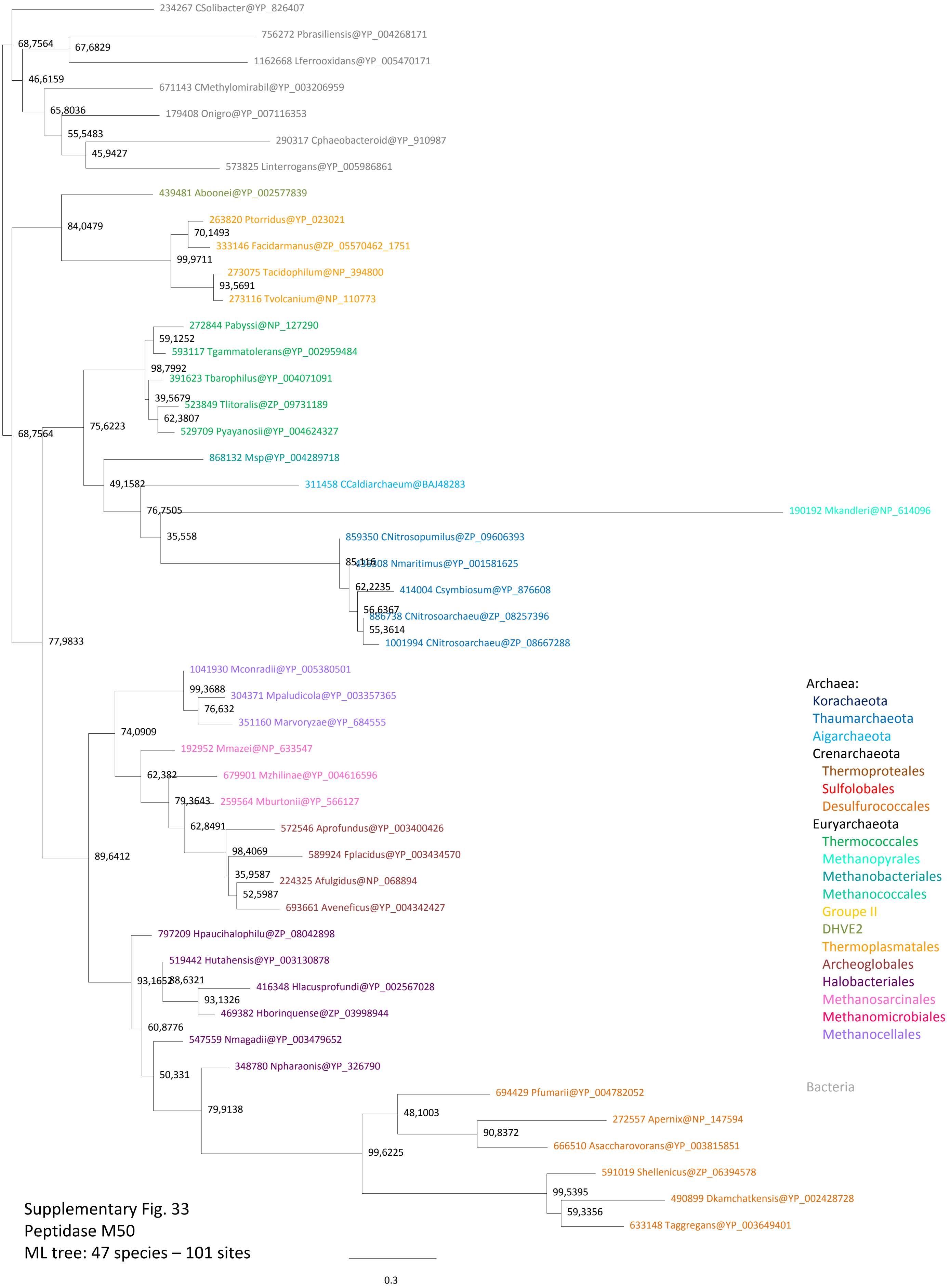


Supplementary Fig. 33
Peptidase M50
ML tree: 47 species – 101 sites

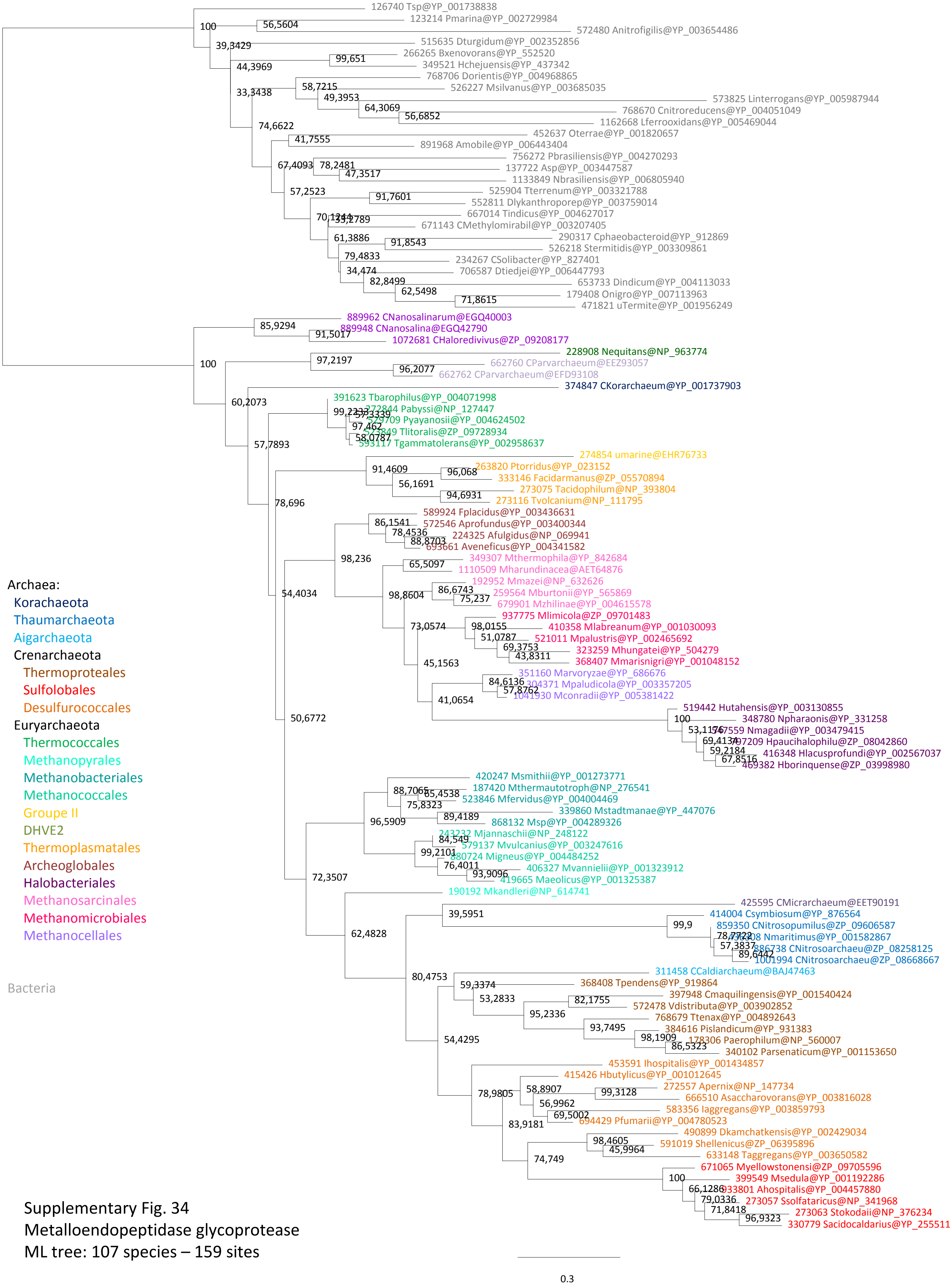


Supplementary Fig. 34
Metalloendopeptidase glycoprotease
ML tree: 107 species – 159 sites

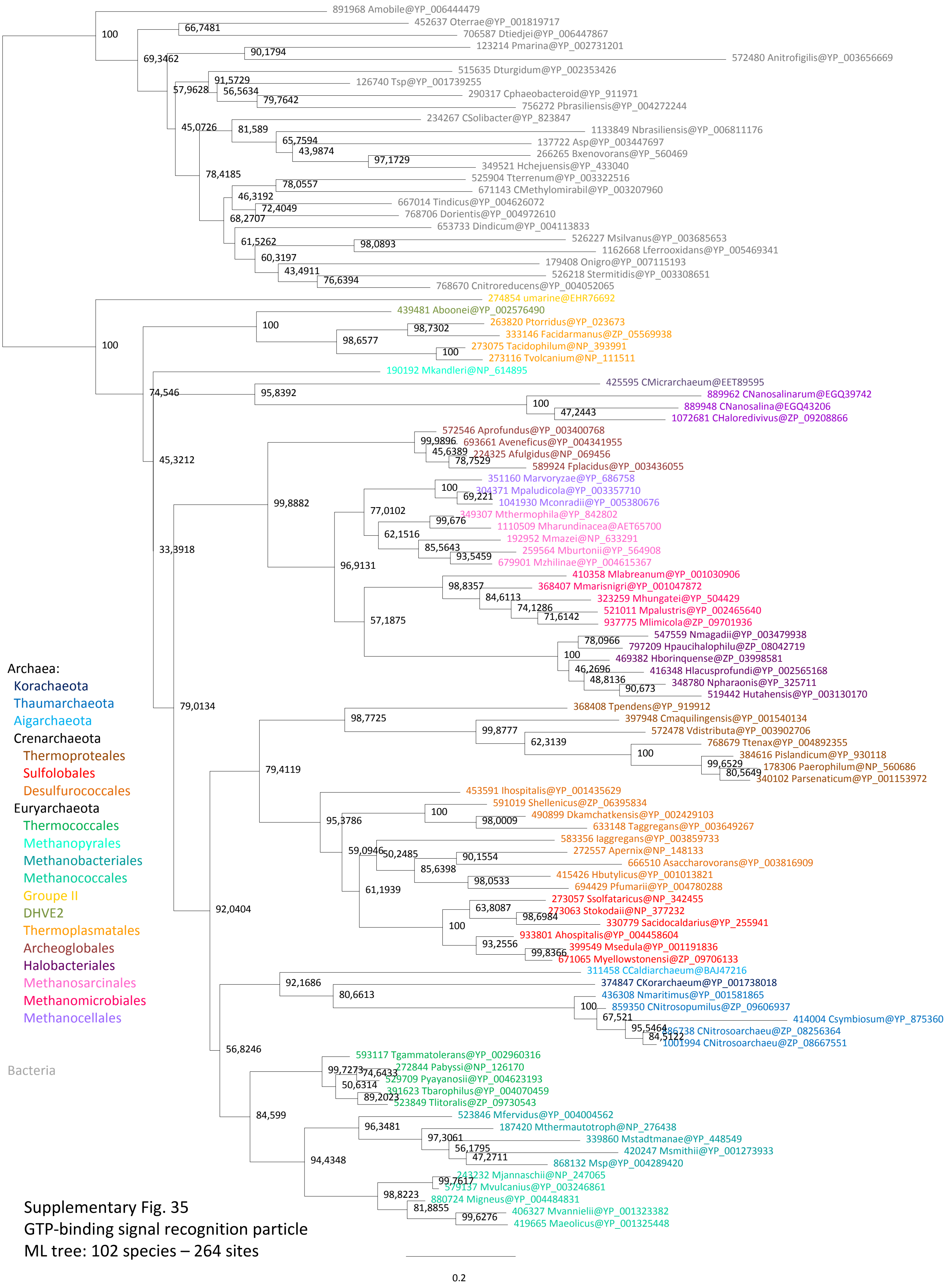


Supplementary Fig. 35
GTP-binding signal recognition particle
ML tree: 102 species – 264 sites

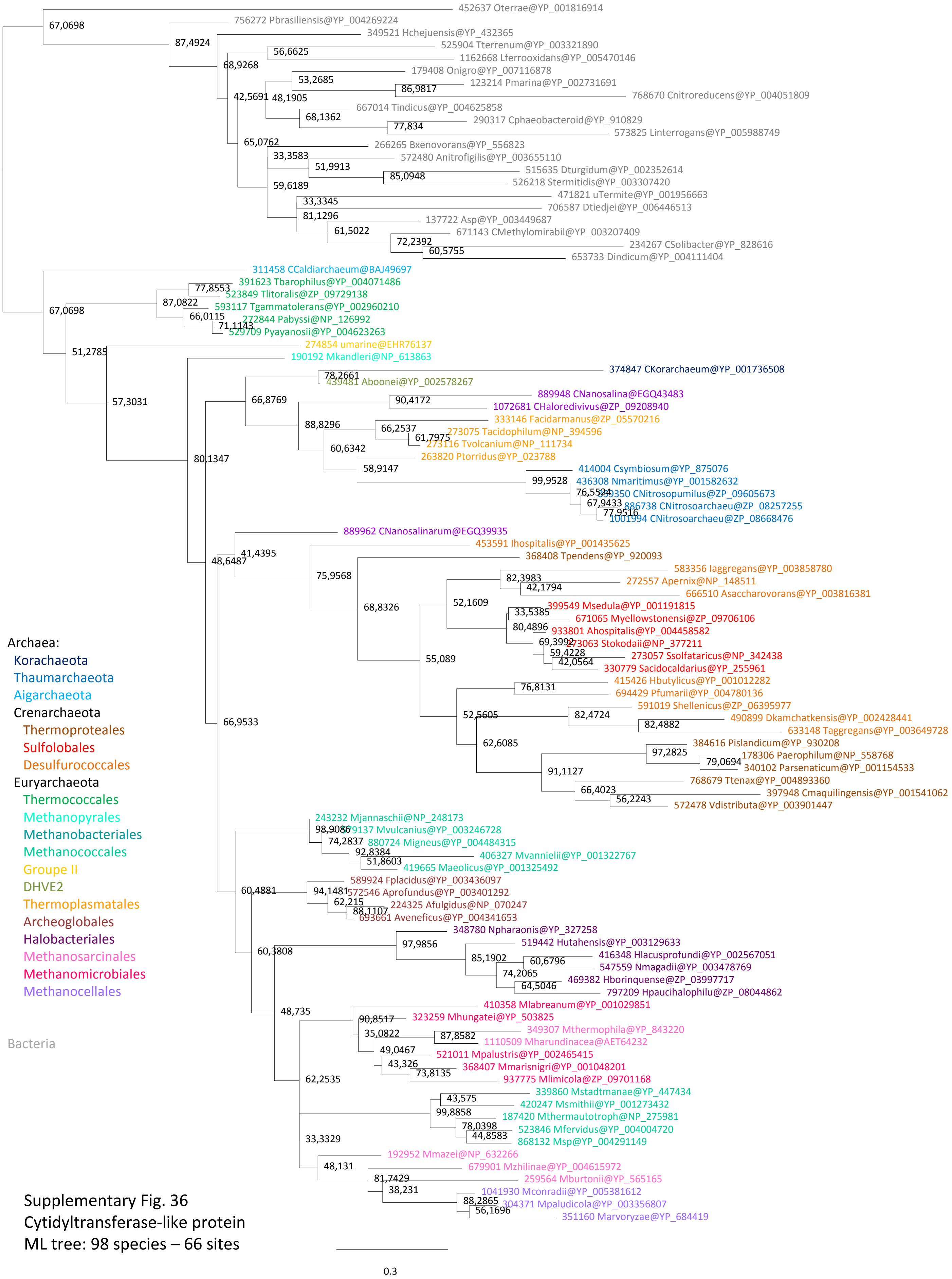


Supplementary Fig. 36
Cytidyltransferase-like protein
ML tree: 98 species – 66 sites

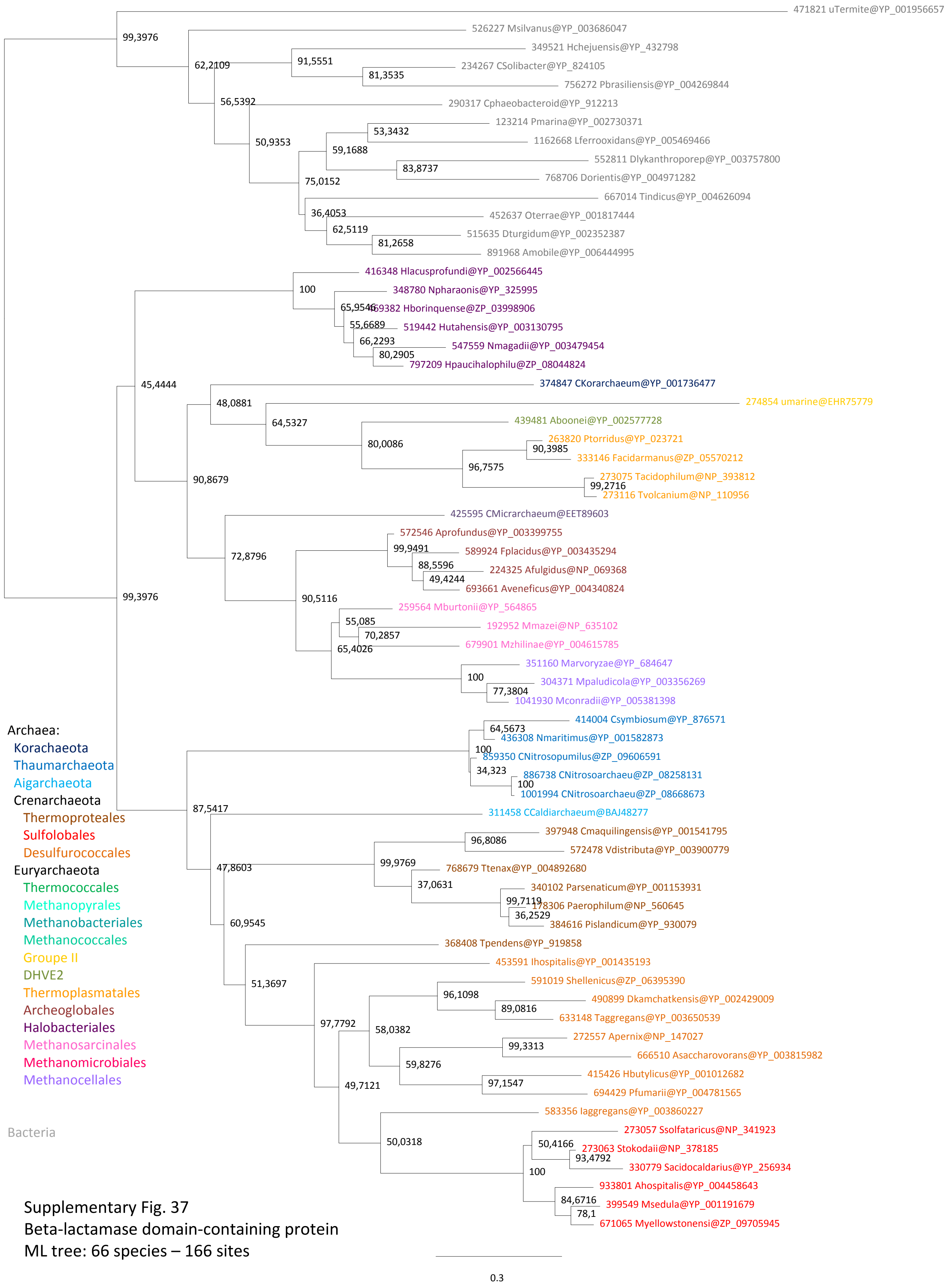


Supplementary Fig. 37
Beta-lactamase domain-containing protein
ML tree: 66 species – 166 sites

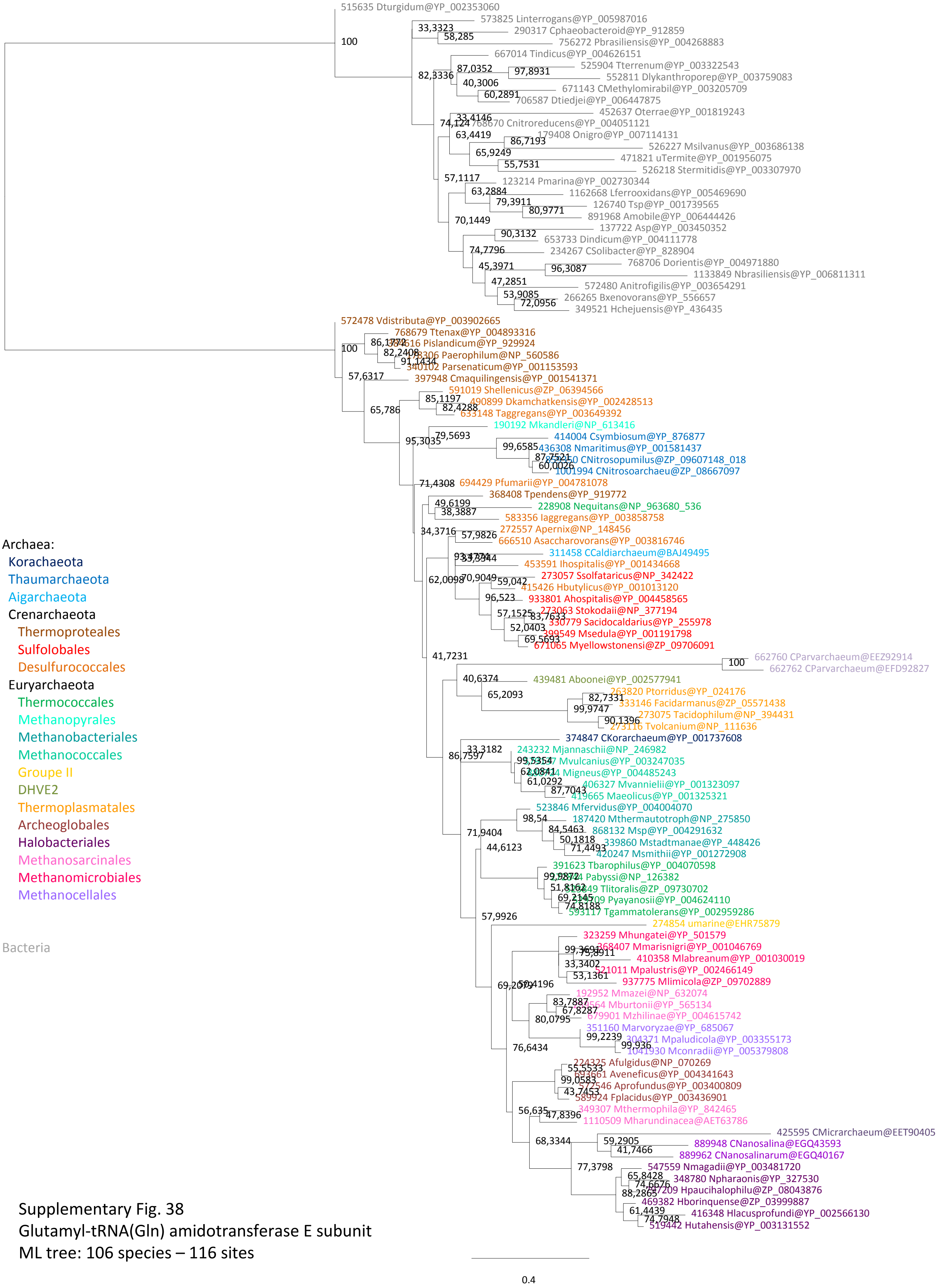


Supplementary Fig. 38

Glutamyl-tRNA(Gln) amidotransferase E subunit

ML tree: 106 species – 116 sites

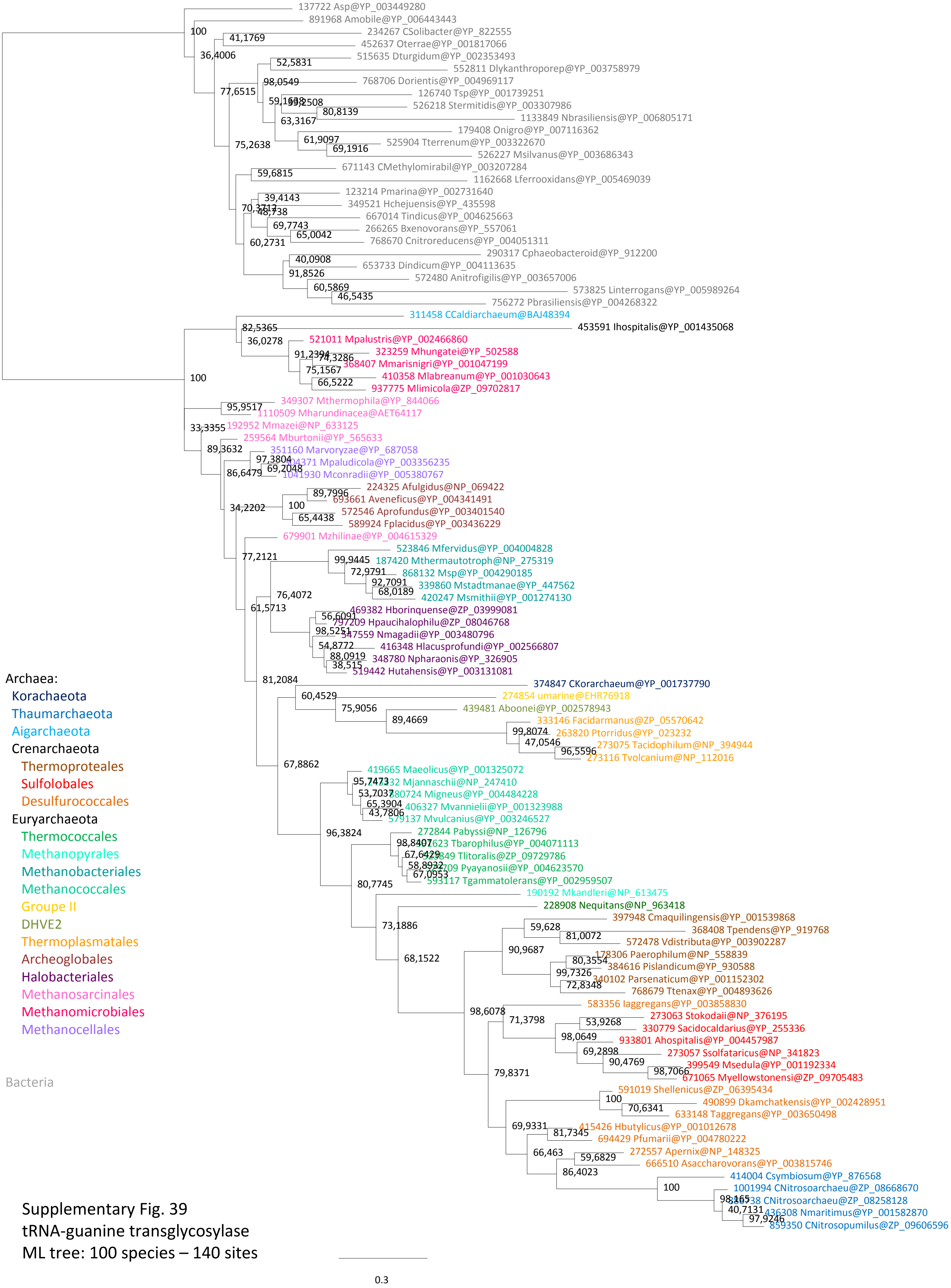


Supplementary Fig. 39
tRNA-guanine transglycosylase
ML tree: 100 species – 140 sites

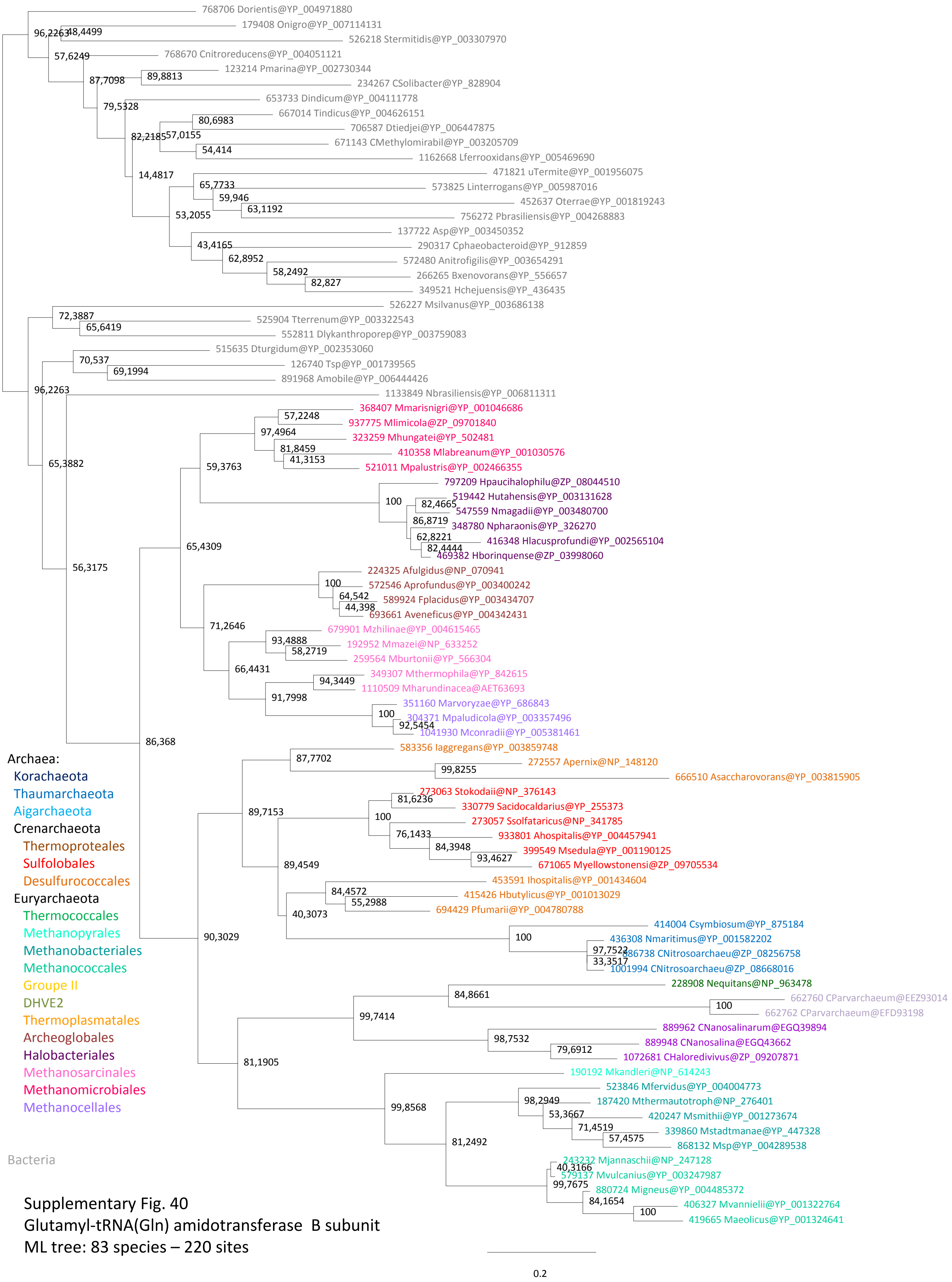


Supplementary Fig. 40

Glutamyl-tRNA(Gln) amidotransferase B subunit

ML tree: 83 species – 220 sites

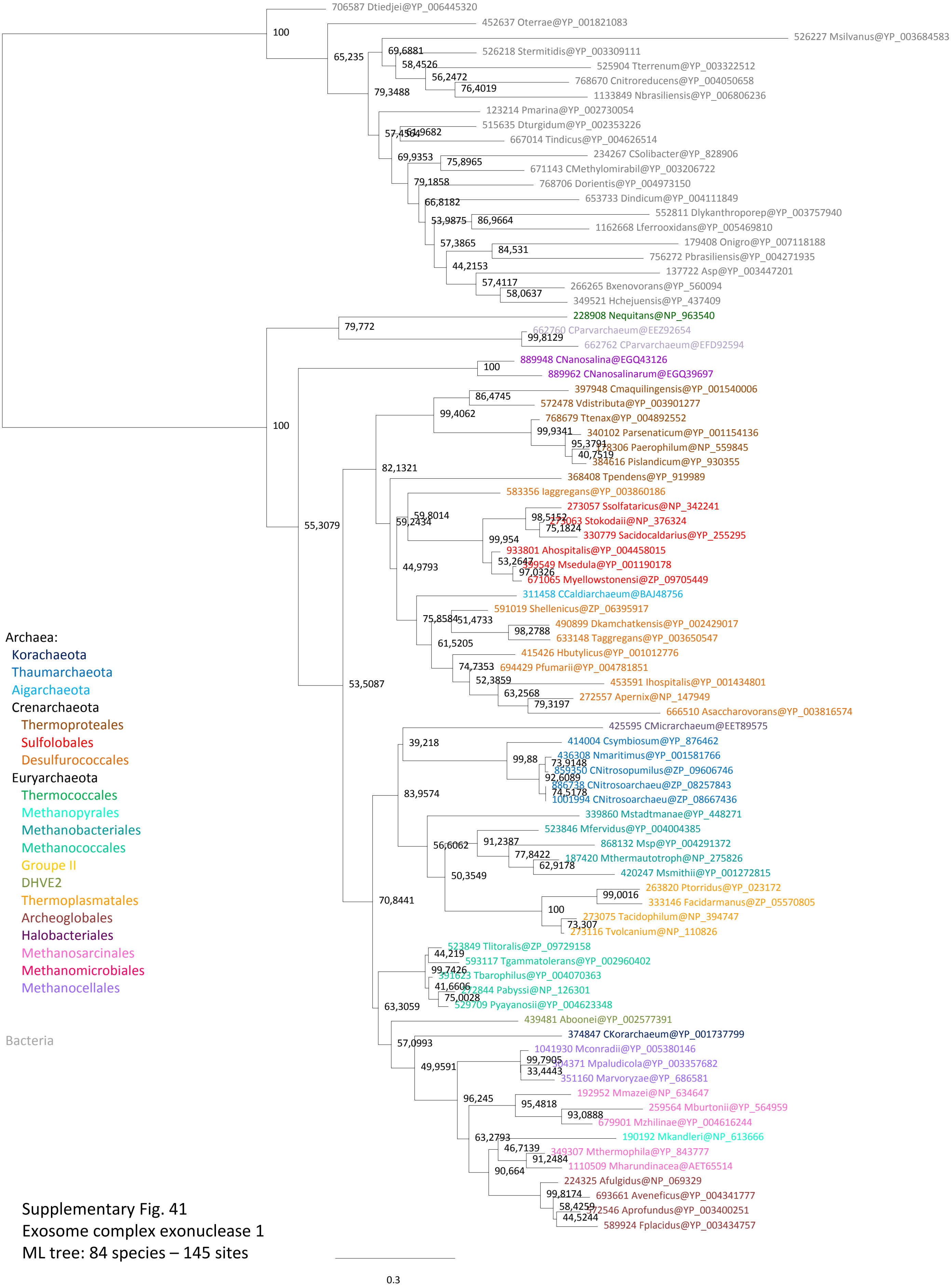


Supplementary Fig. 41

Exosome complex exonuclease 1

ML tree: 84 species – 145 sites

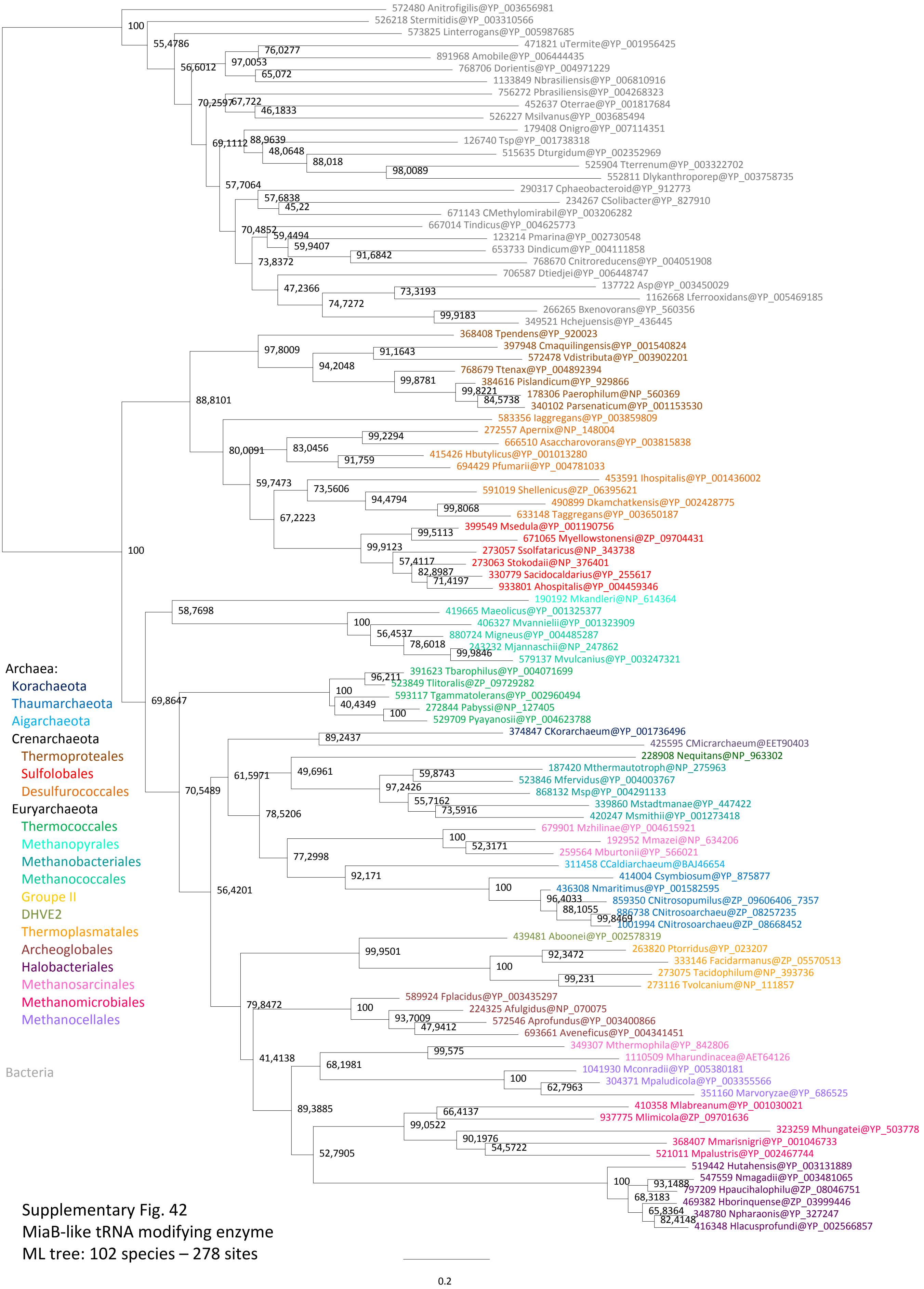


Supplementary Fig. 42
MiaB-like tRNA modifying enzyme
ML tree: 102 species – 278 sites

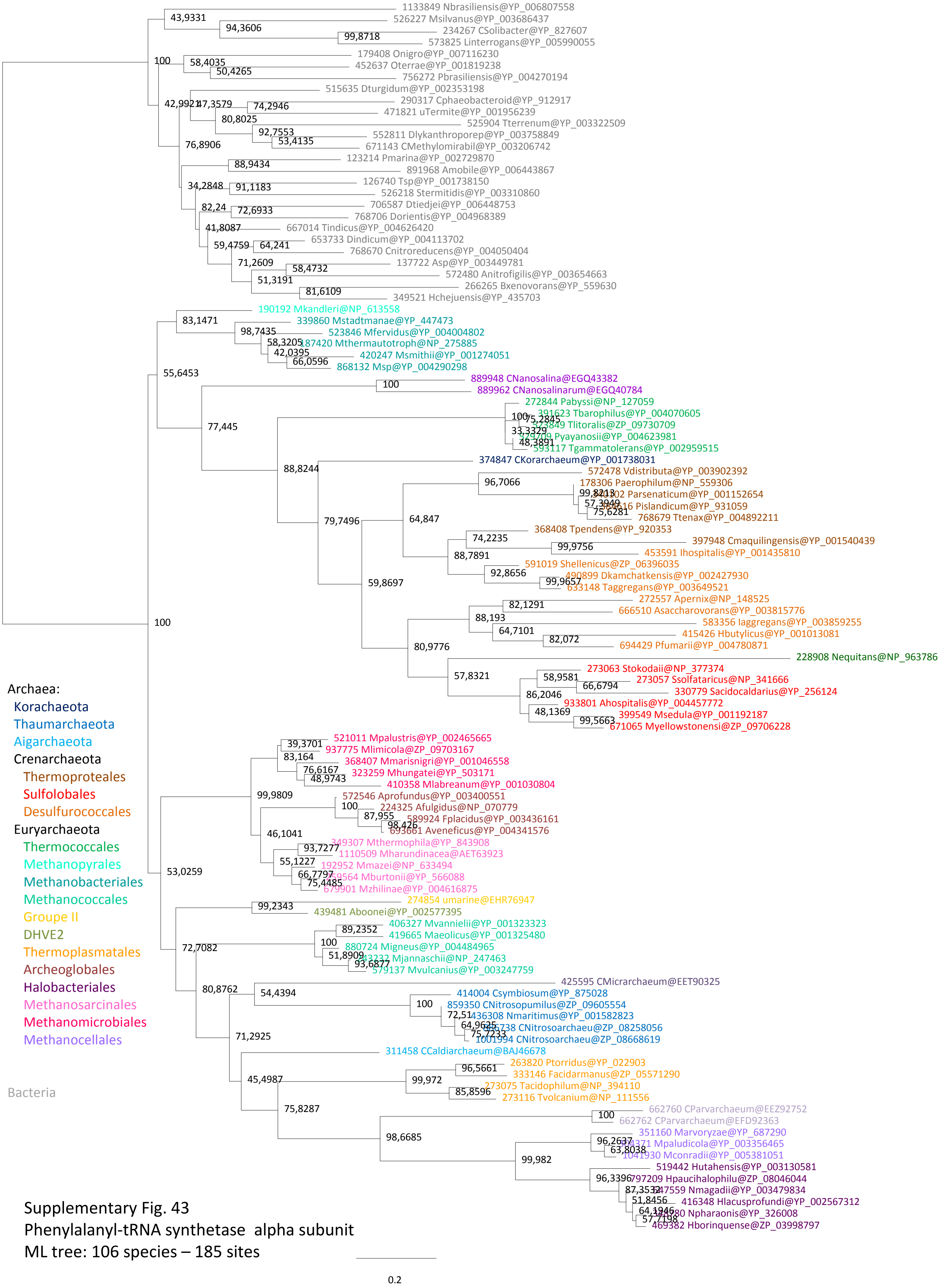


Supplementary Fig. 43
Phenylalanyl-tRNA synthetase alpha subunit
ML tree: 106 species – 185 sites

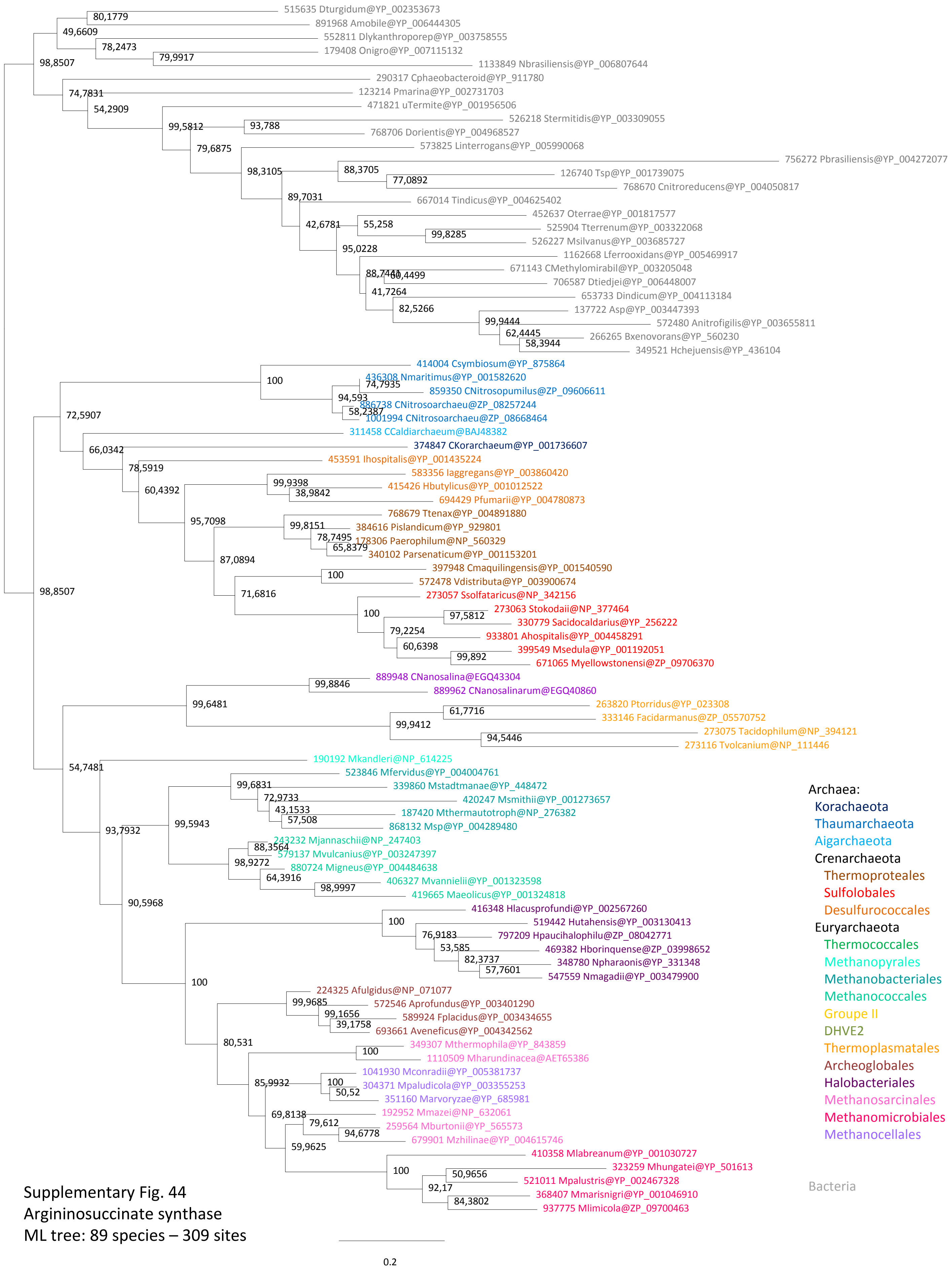


Supplementary Fig. 44
Argininosuccinate synthase
ML tree: 89 species – 309 sites

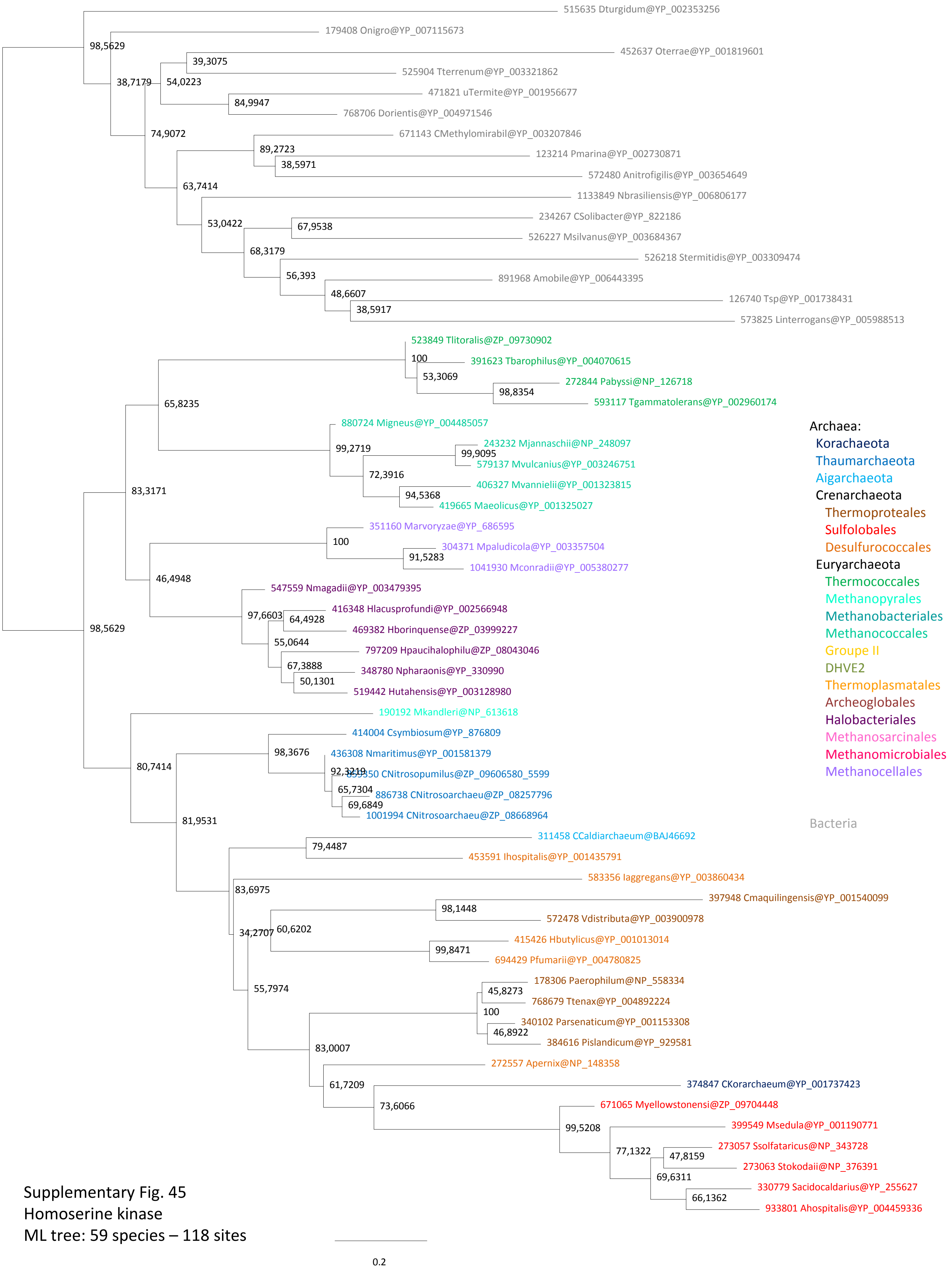


Supplementary Fig. 45
Homoserine kinase
ML tree: 59 species – 118 sites

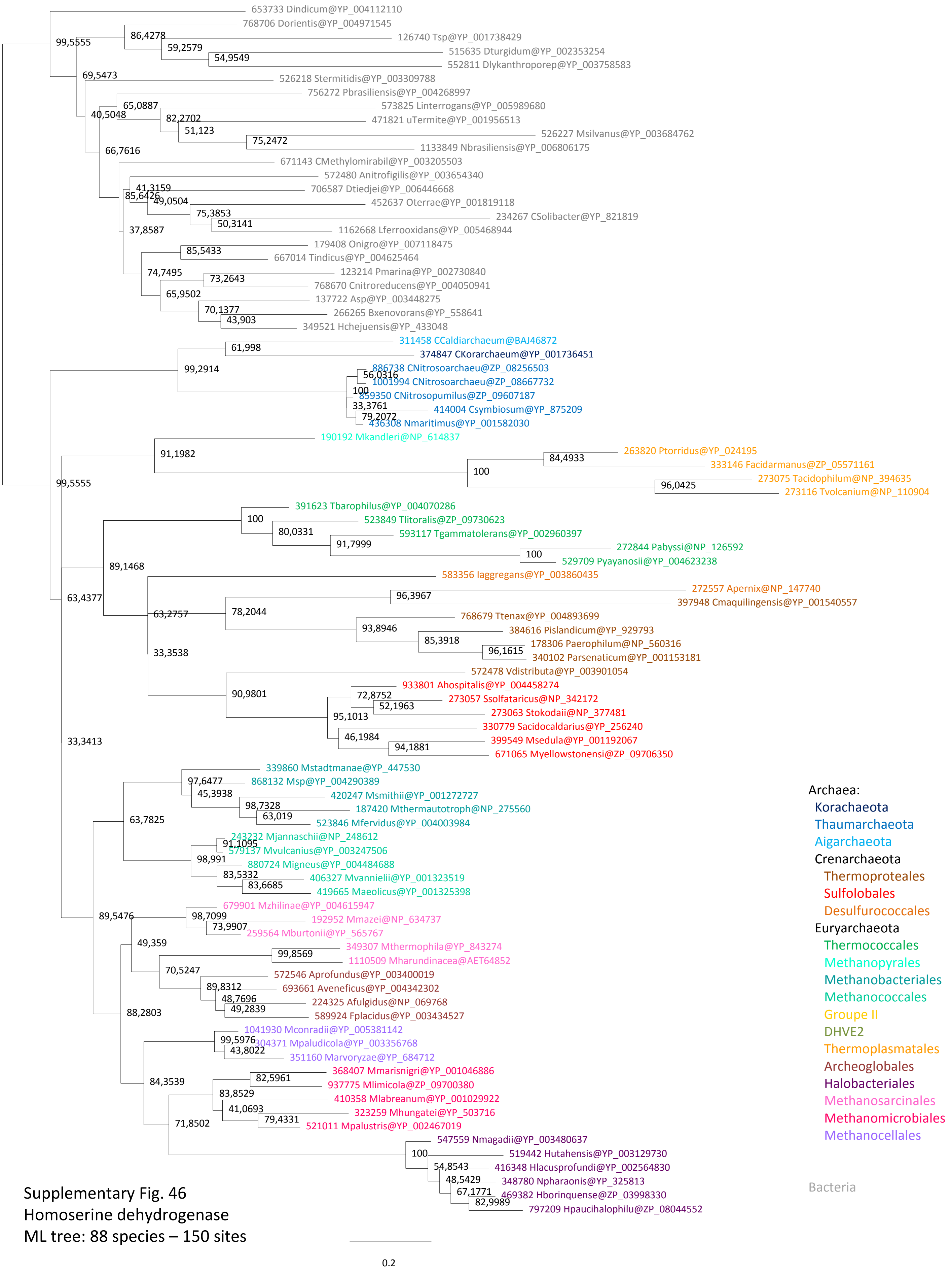


Supplementary Fig. 46
Homoserine dehydrogenase
ML tree: 88 species – 150 sites

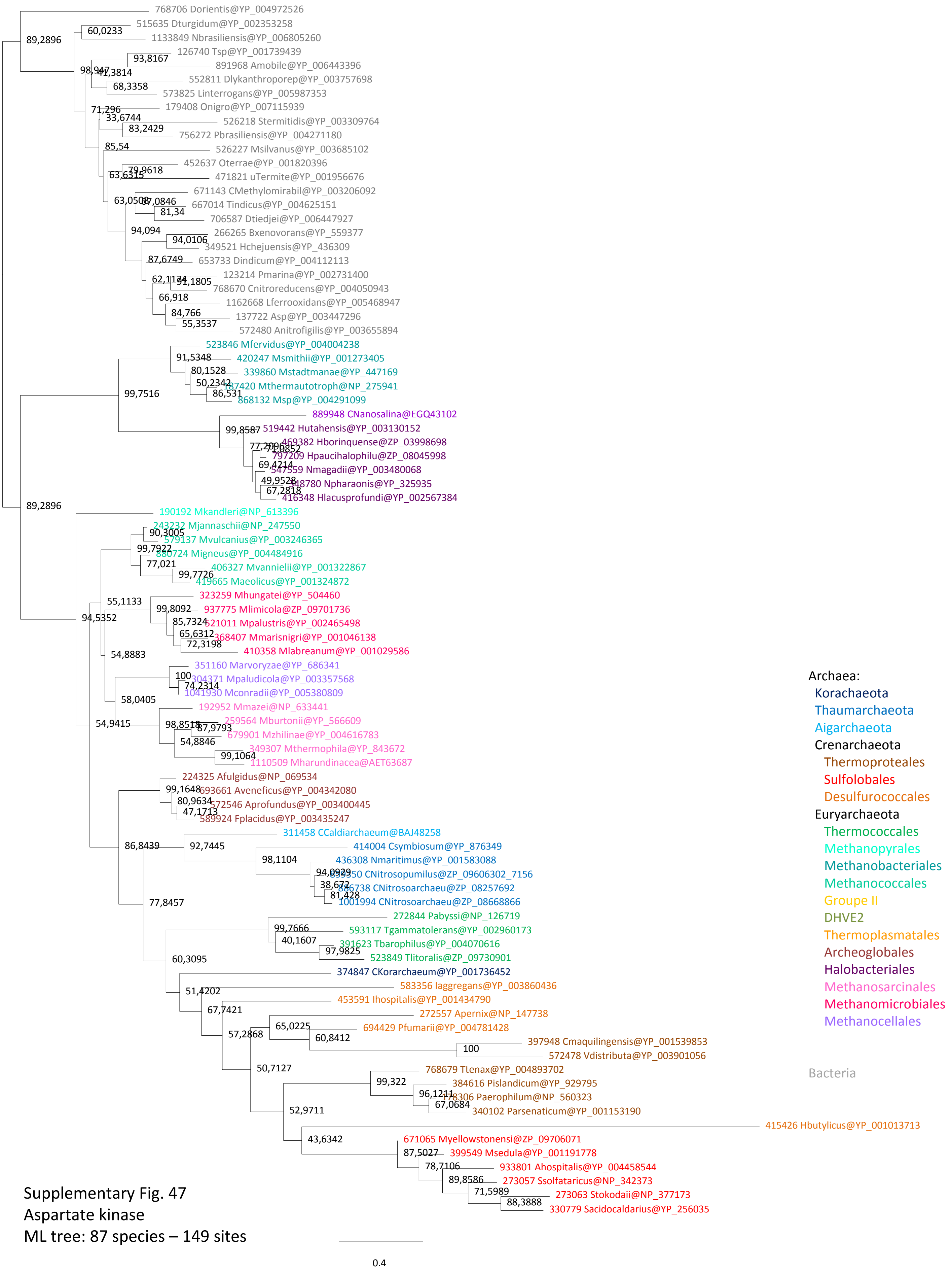


Supplementary Fig. 47
Aspartate kinase
ML tree: 87 species – 149 sites

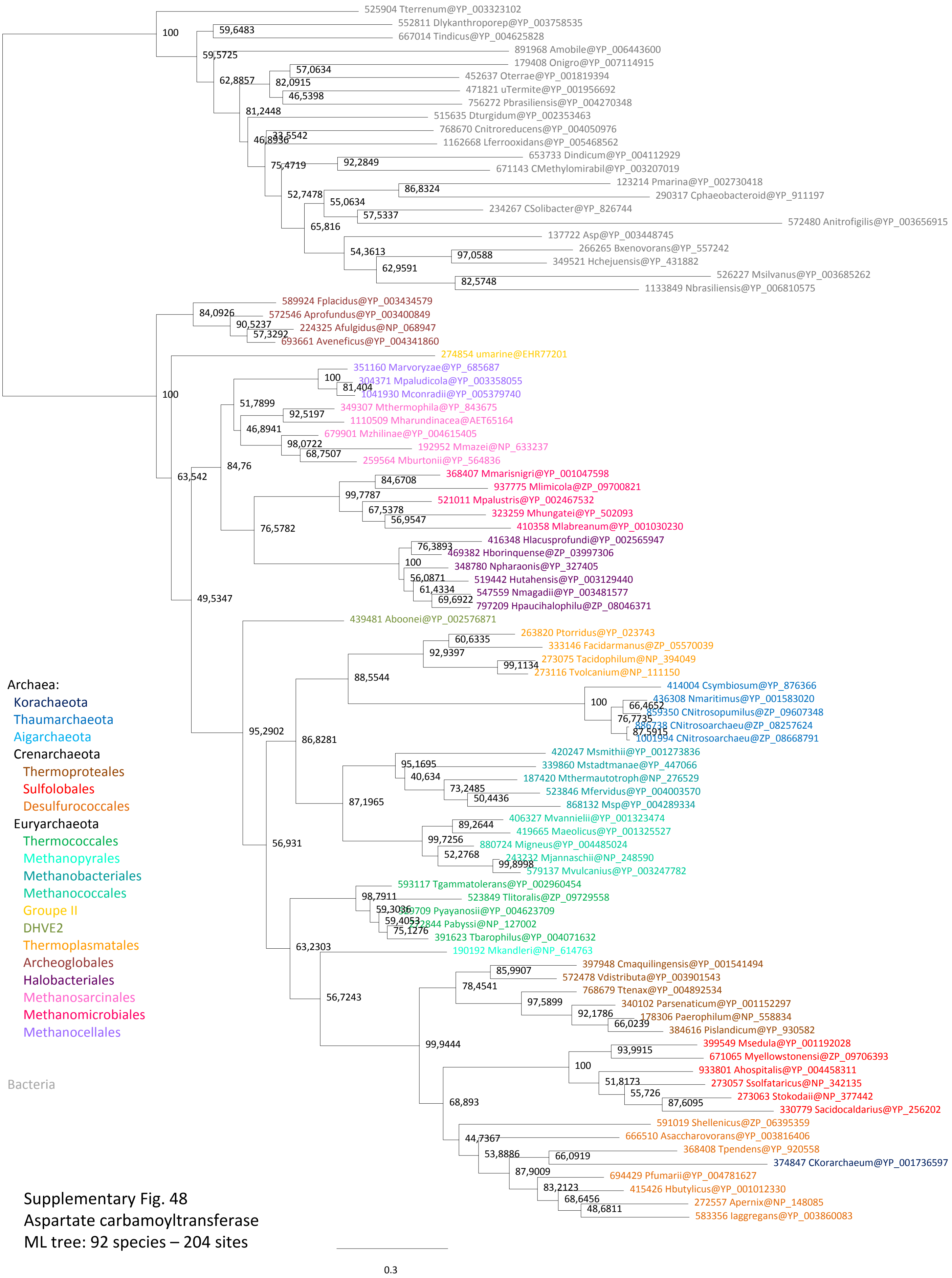


Supplementary Fig. 48
Aspartate carbamoyltransferase
ML tree: 92 species – 204 sites

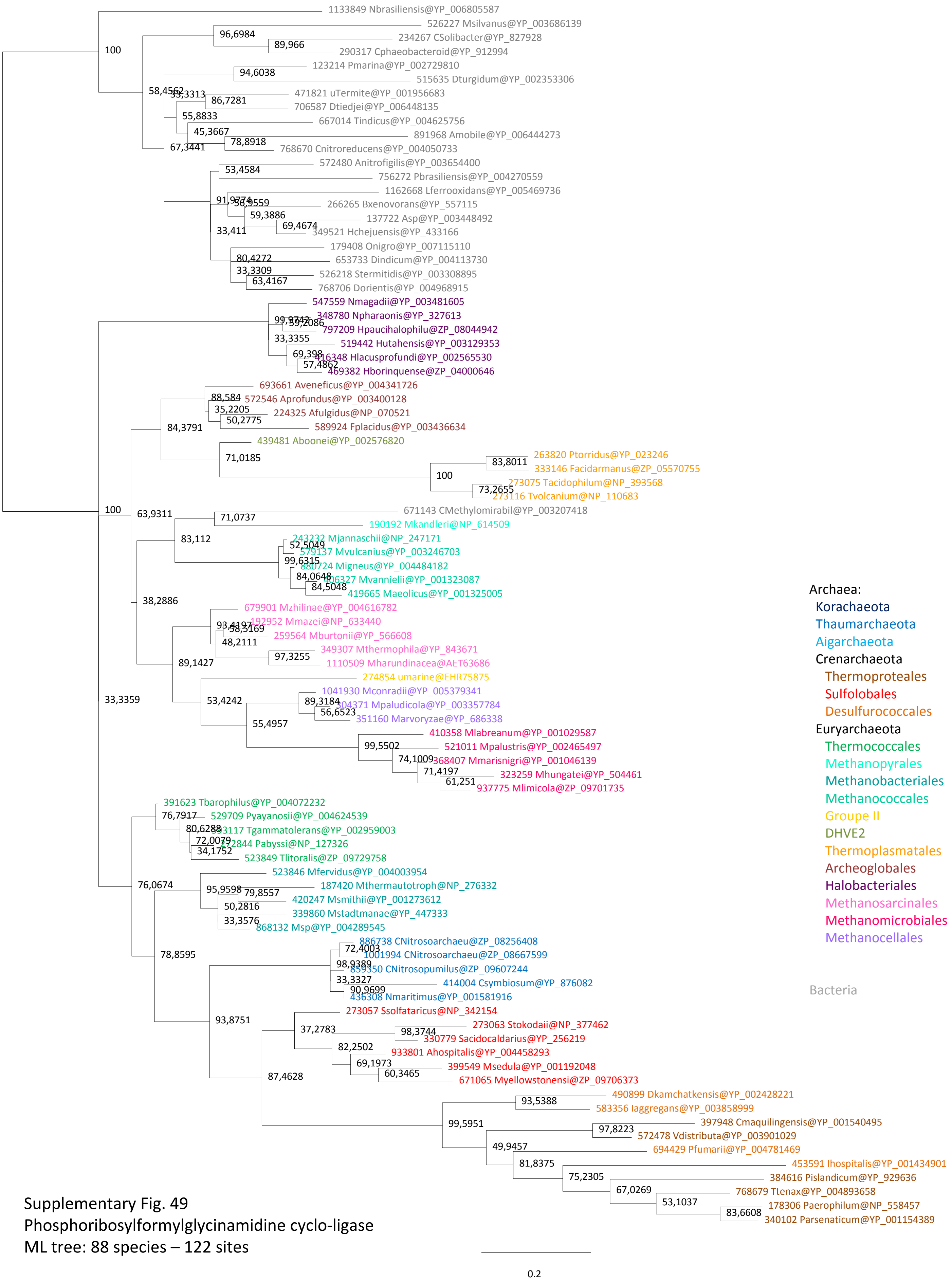


Supplementary Fig. 49
Phosphoribosylformylglycinamidine cyclo-ligase
ML tree: 88 species – 122 sites

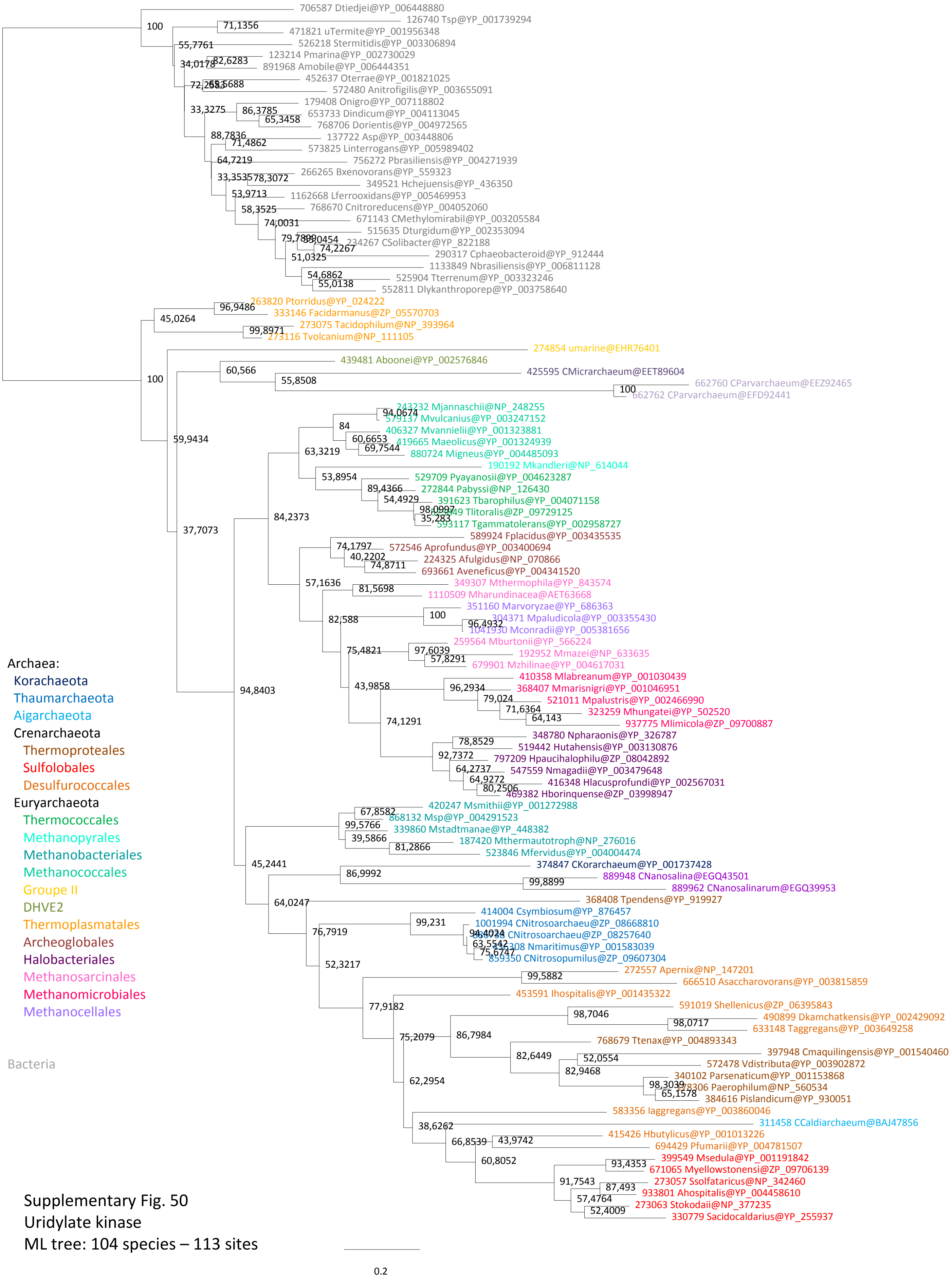


Supplementary Fig. 50
Uridylate kinase
ML tree: 104 species – 113 sites

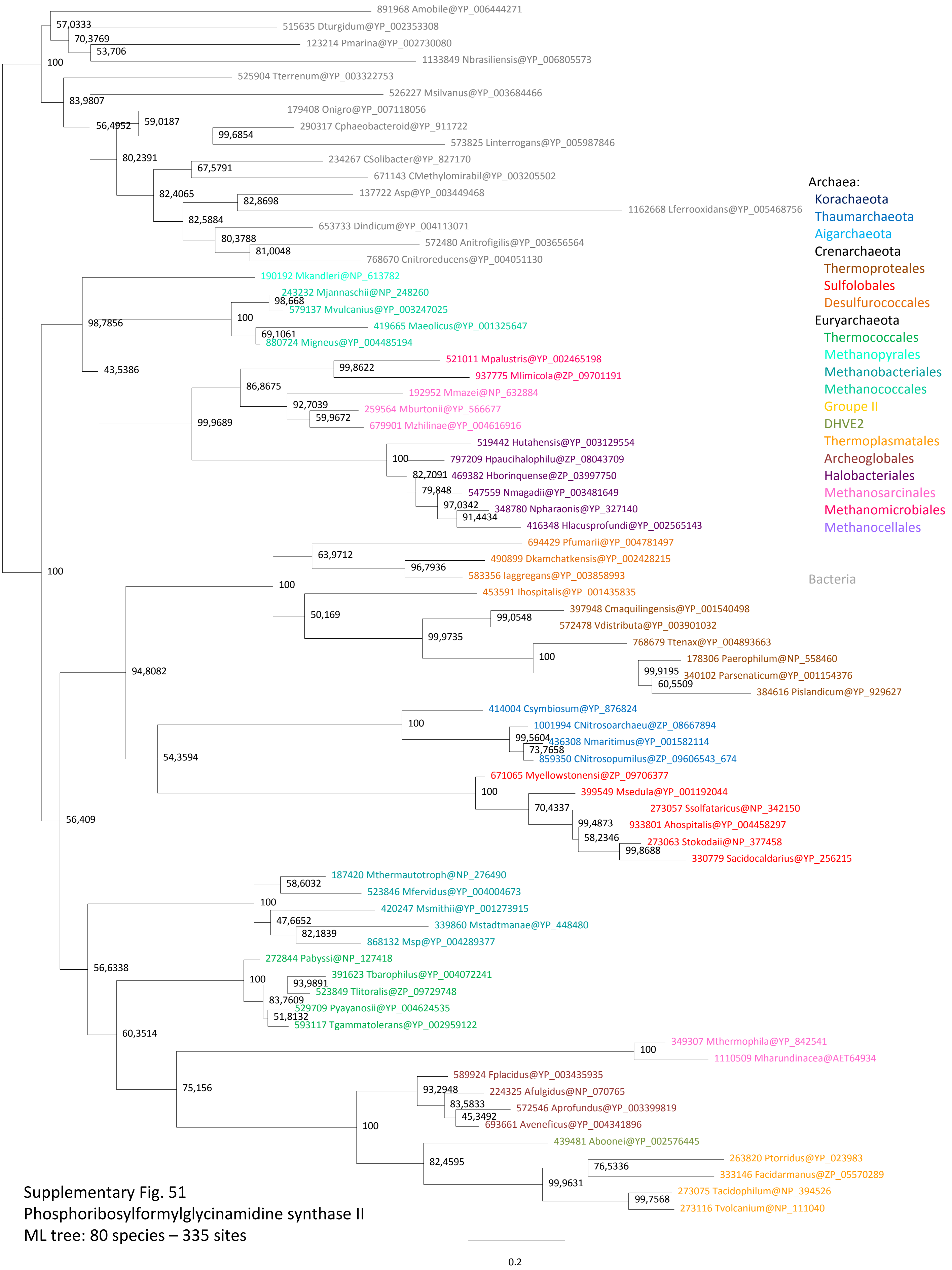


Supplementary Fig. 51
Phosphoribosylformylglycinamidine synthase II
ML tree: 80 species – 335 sites

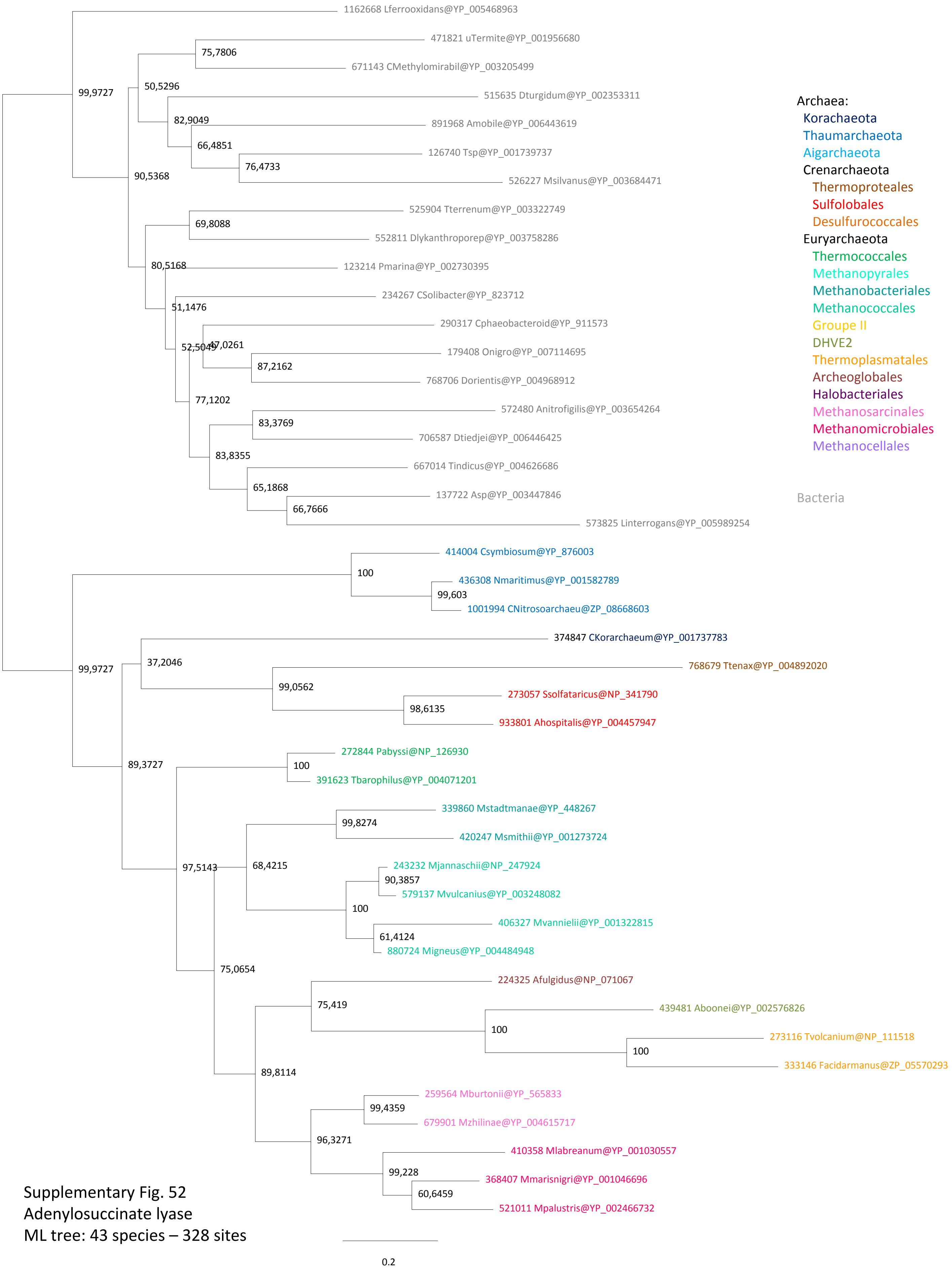


Supplementary Fig. 52
Adenylosuccinate lyase
ML tree: 43 species – 328 sites

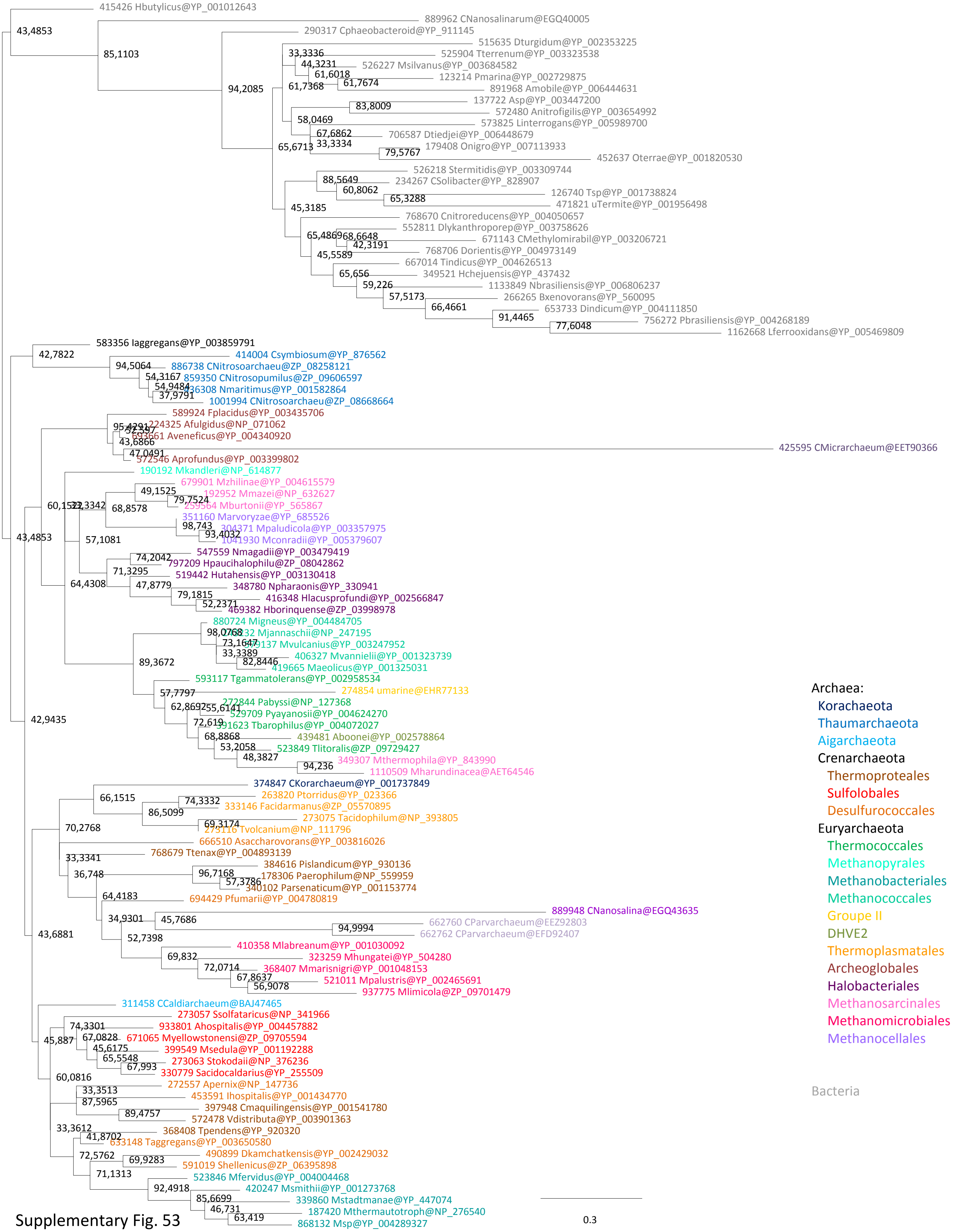


Supplementary Fig. 53

RdgB/HAM1 purine NTP pyrophosphatase

ML tree: 106 species – 70 sites

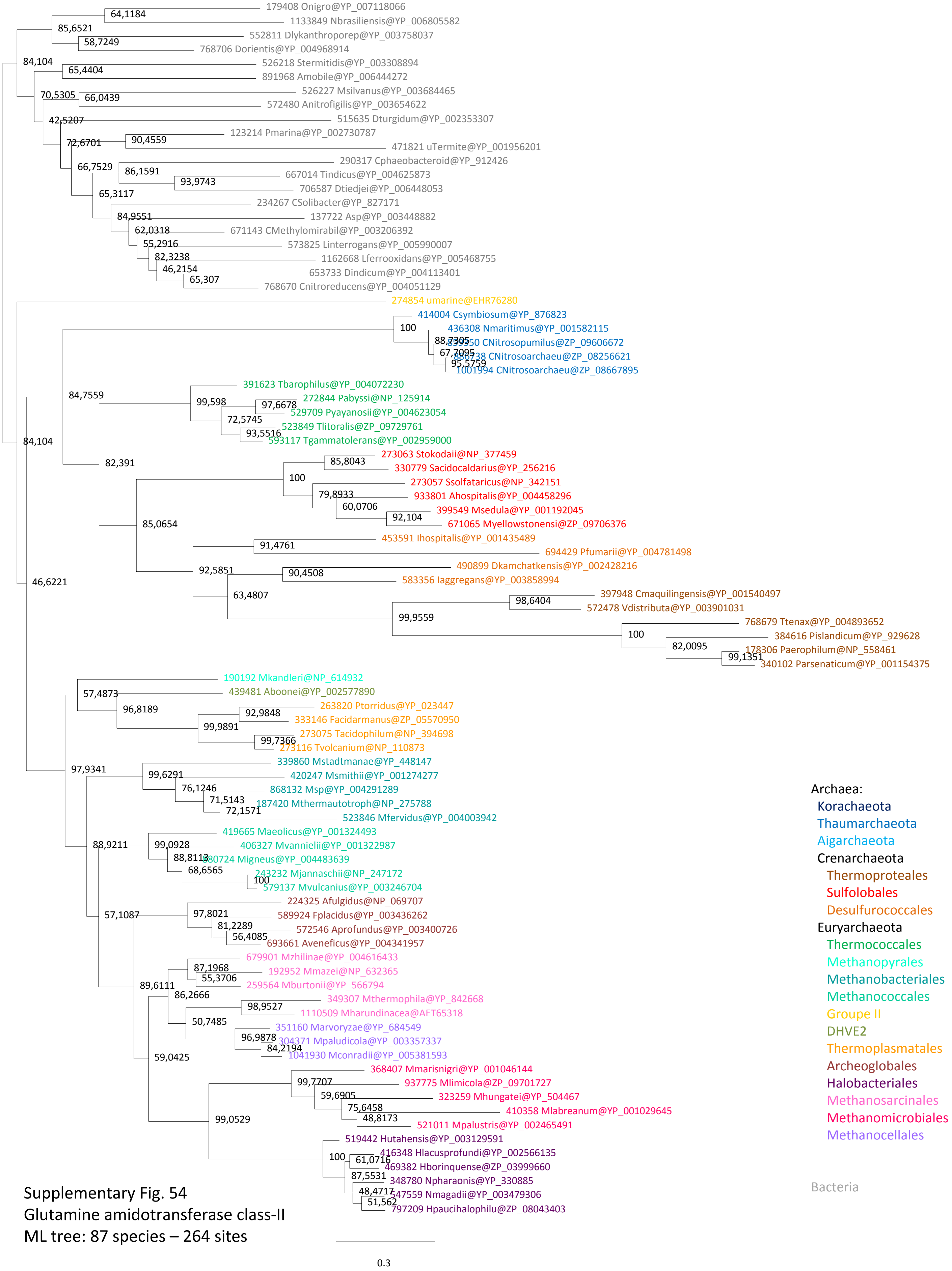


Supplementary Fig. 54
Glutamine amidotransferase class-II
ML tree: 87 species – 264 sites

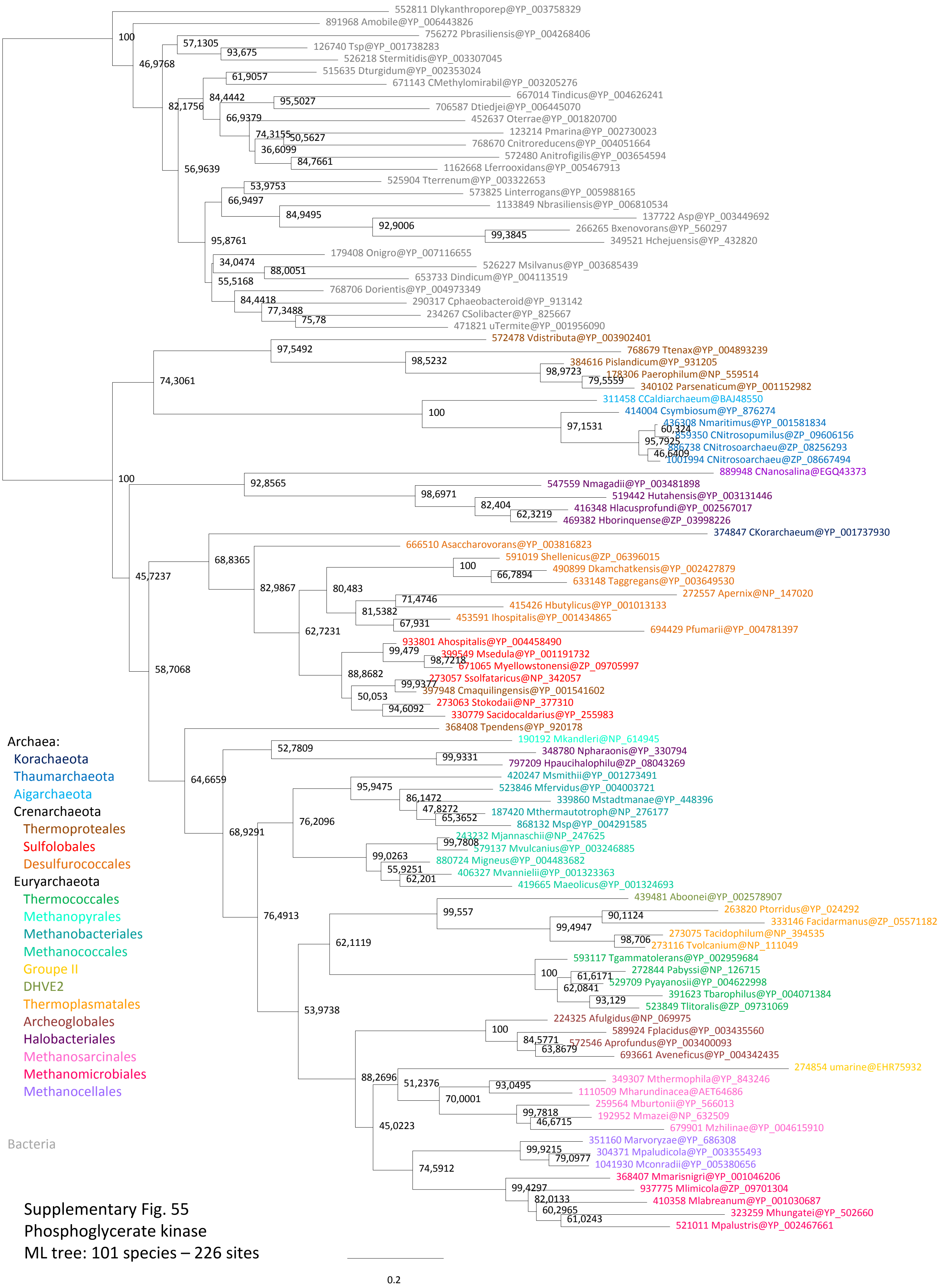


Supplementary Fig. 55
Phosphoglycerate kinase
ML tree: 101 species – 226 sites

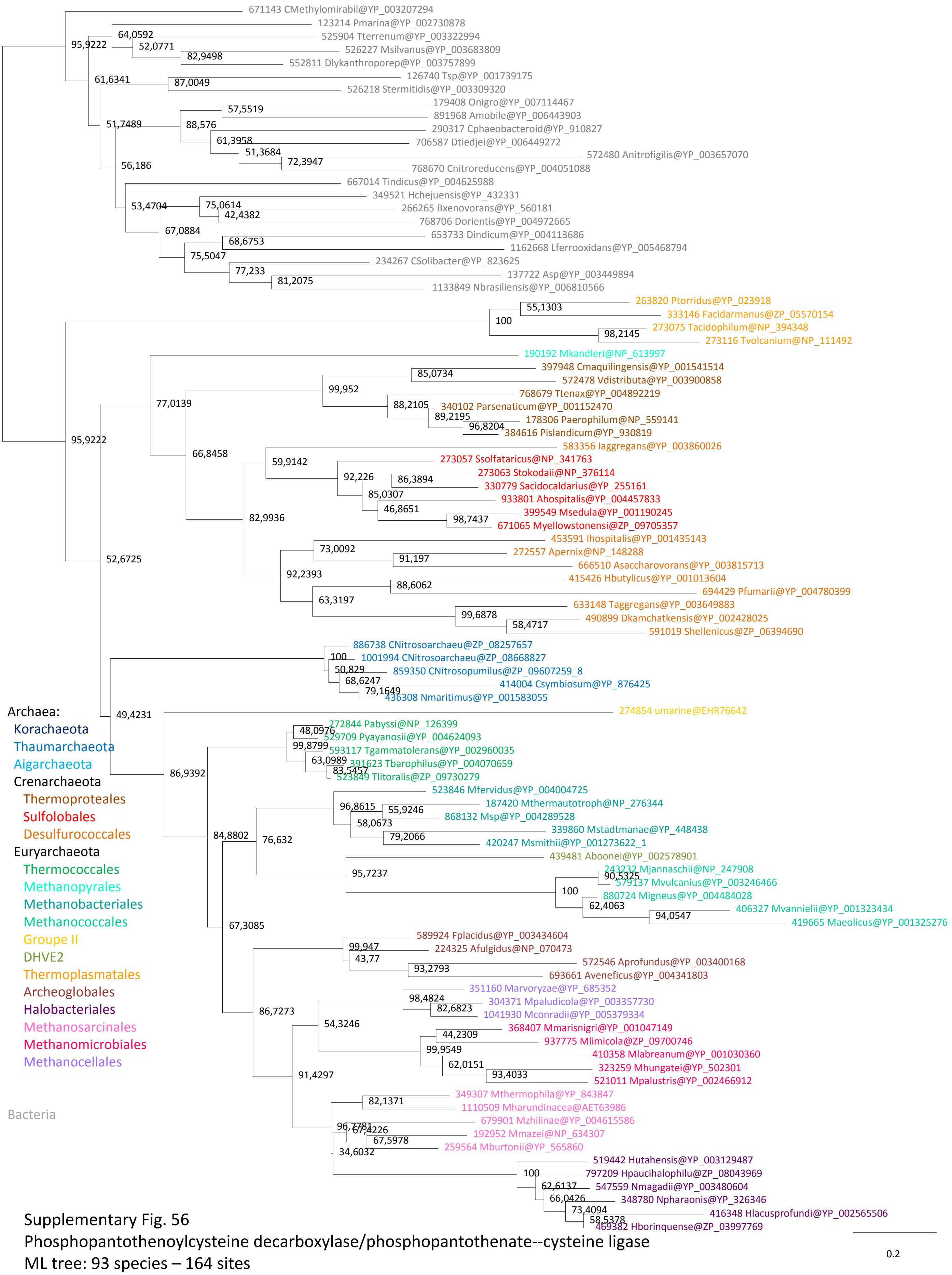


Supplementary Fig. 56
Phosphopantothenoylcysteine decarboxylase/phosphopantothenate--cysteine ligase
ML tree: 93 species – 164 sites

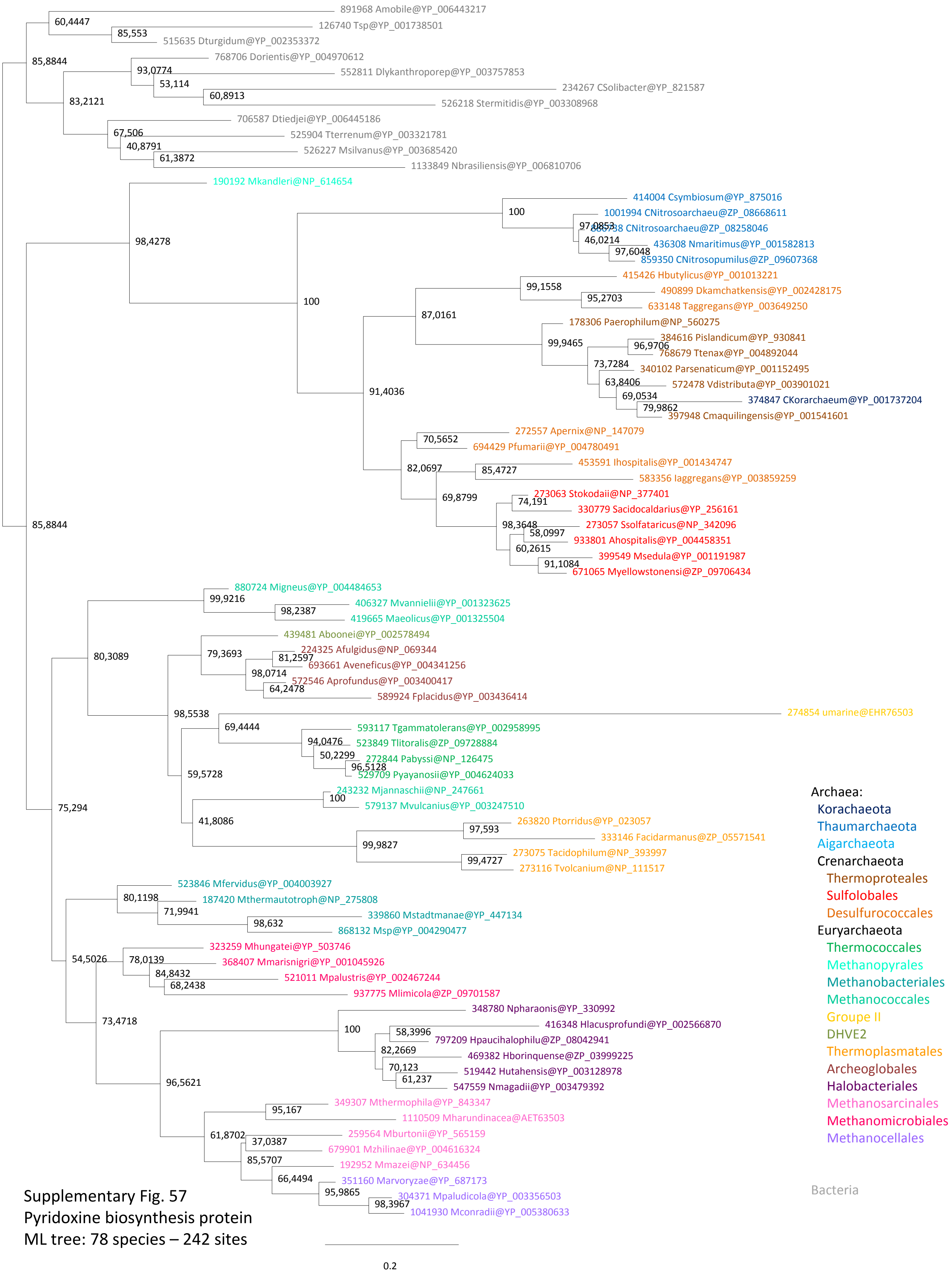


Supplementary Fig. 57
Pyridoxine biosynthesis protein
ML tree: 78 species – 242 sites

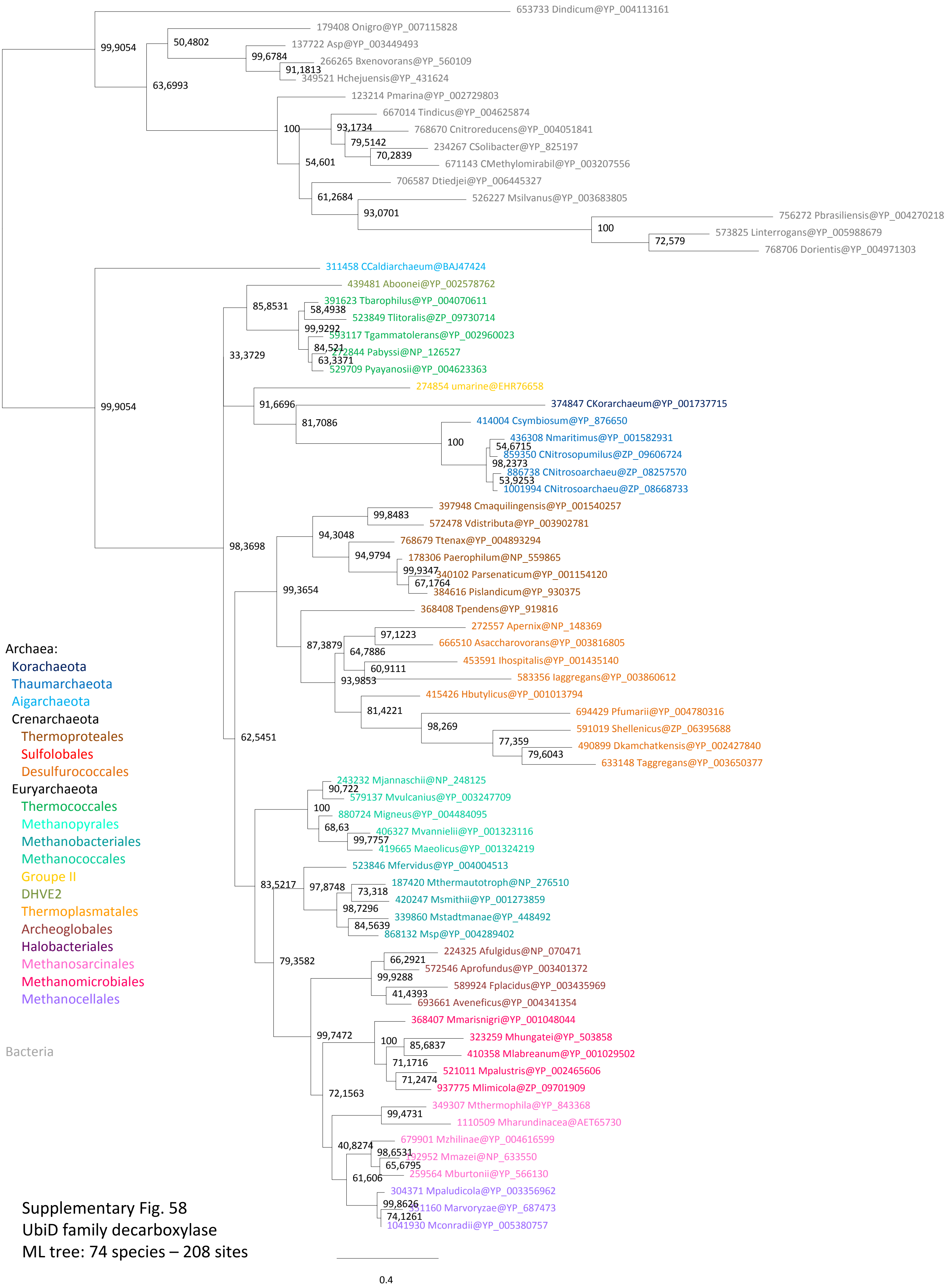


Supplementary Fig. 58
UbiD family decarboxylase
ML tree: 74 species – 208 sites

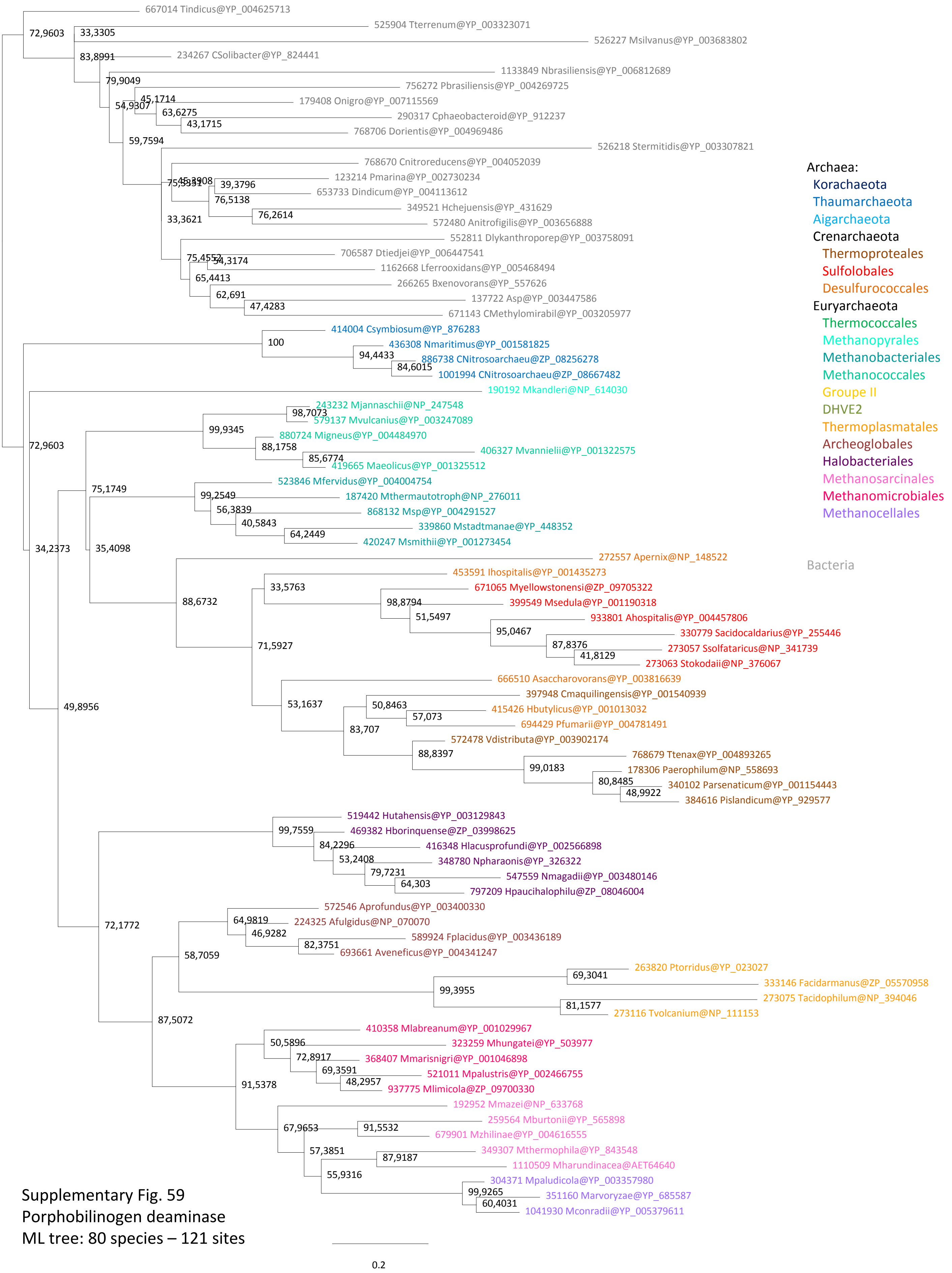


Supplementary Fig. 59
Porphobilinogen deaminase
ML tree: 80 species – 121 sites

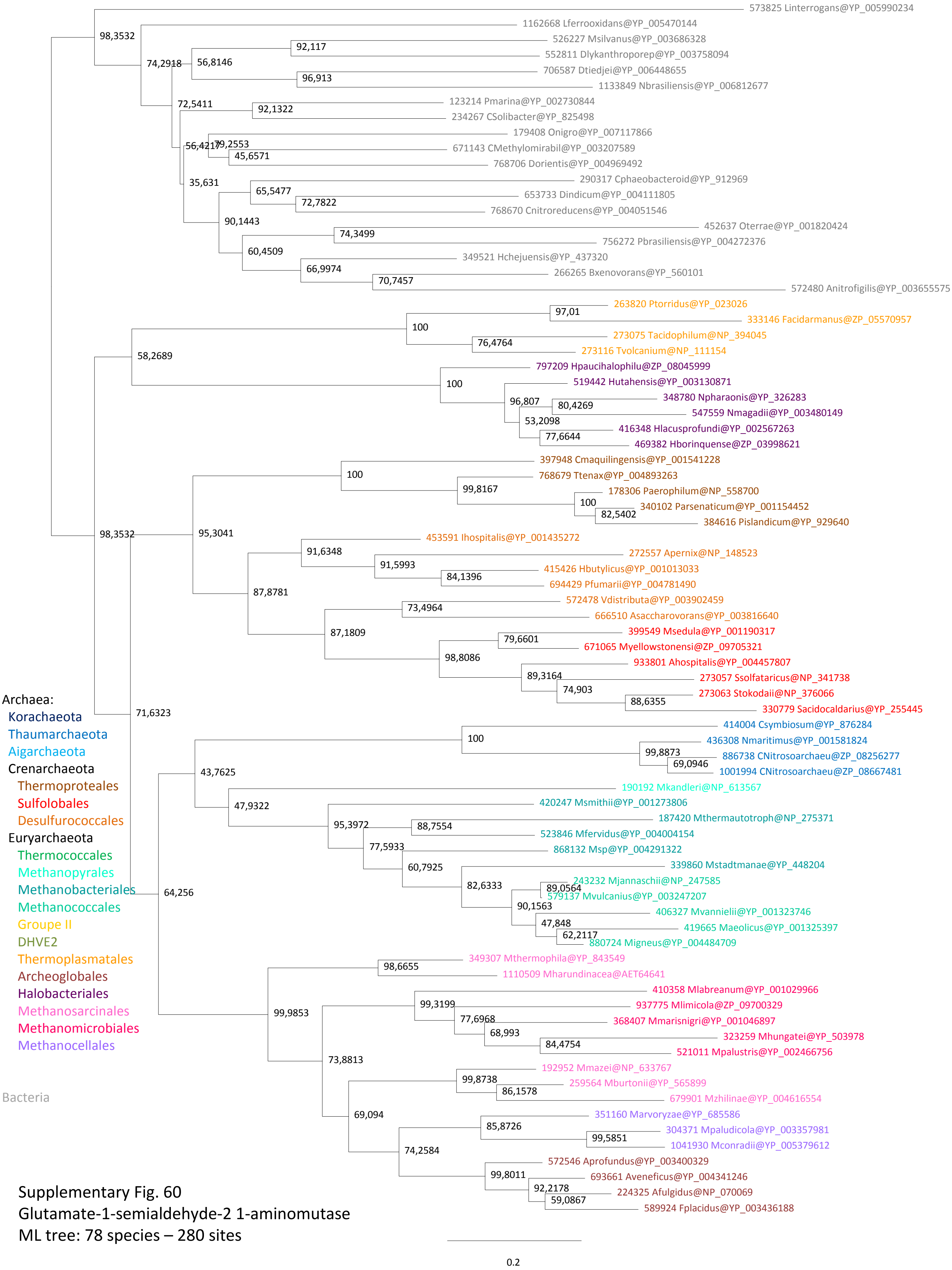


Supplementary Fig. 60
Glutamate-1-semialdehyde-2 1-aminomutase
ML tree: 78 species – 280 sites

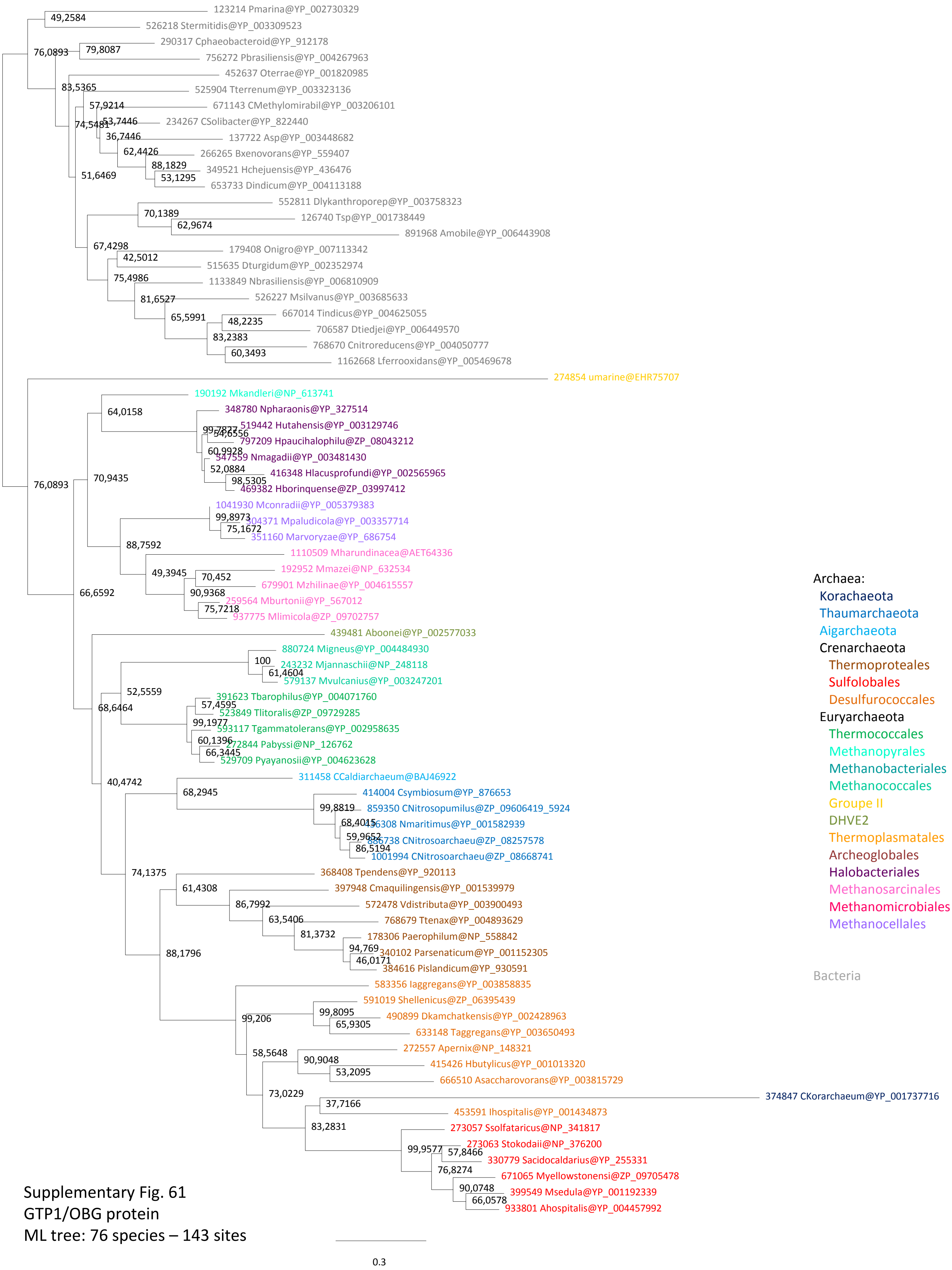


Supplementary Fig. 61
GTP1/OBG protein
ML tree: 76 species – 143 sites

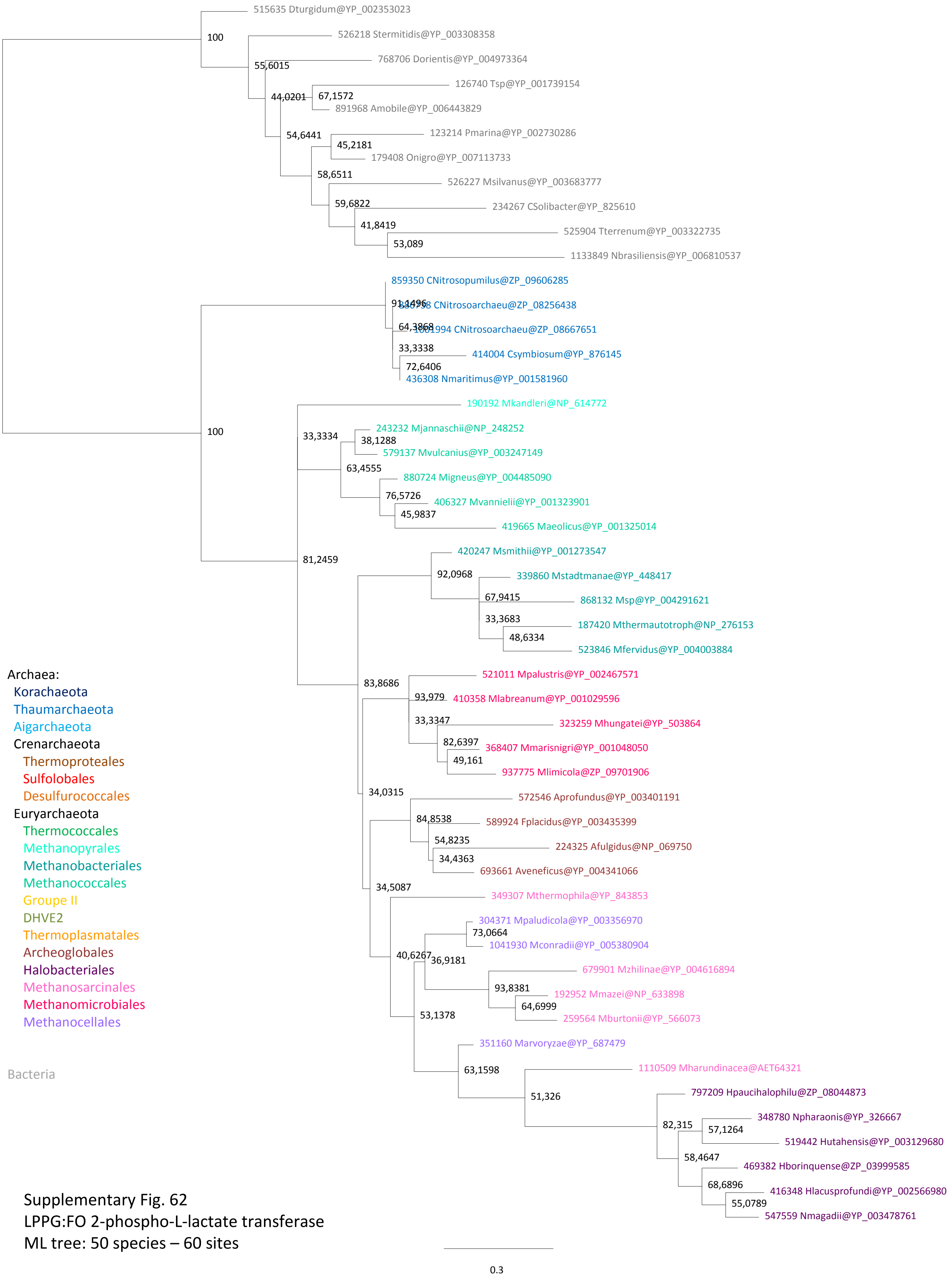


Supplementary Fig. 62
LPPG:FO 2-phospho-L-lactate transferase
ML tree: 50 species – 60 sites

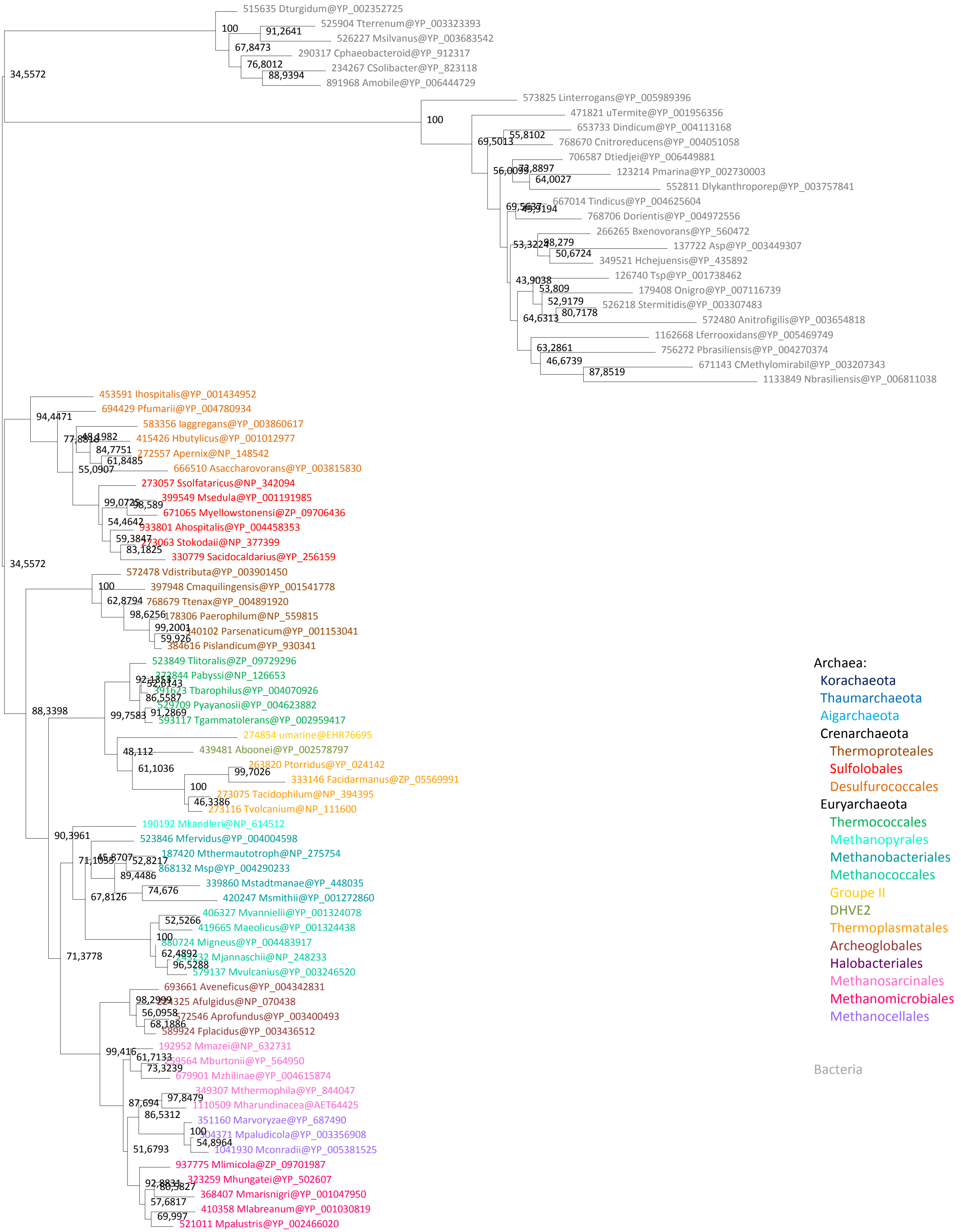


Supplementary Fig. 63
Prolyl-tRNA synthetase
ML tree: 83 species – 186 sites

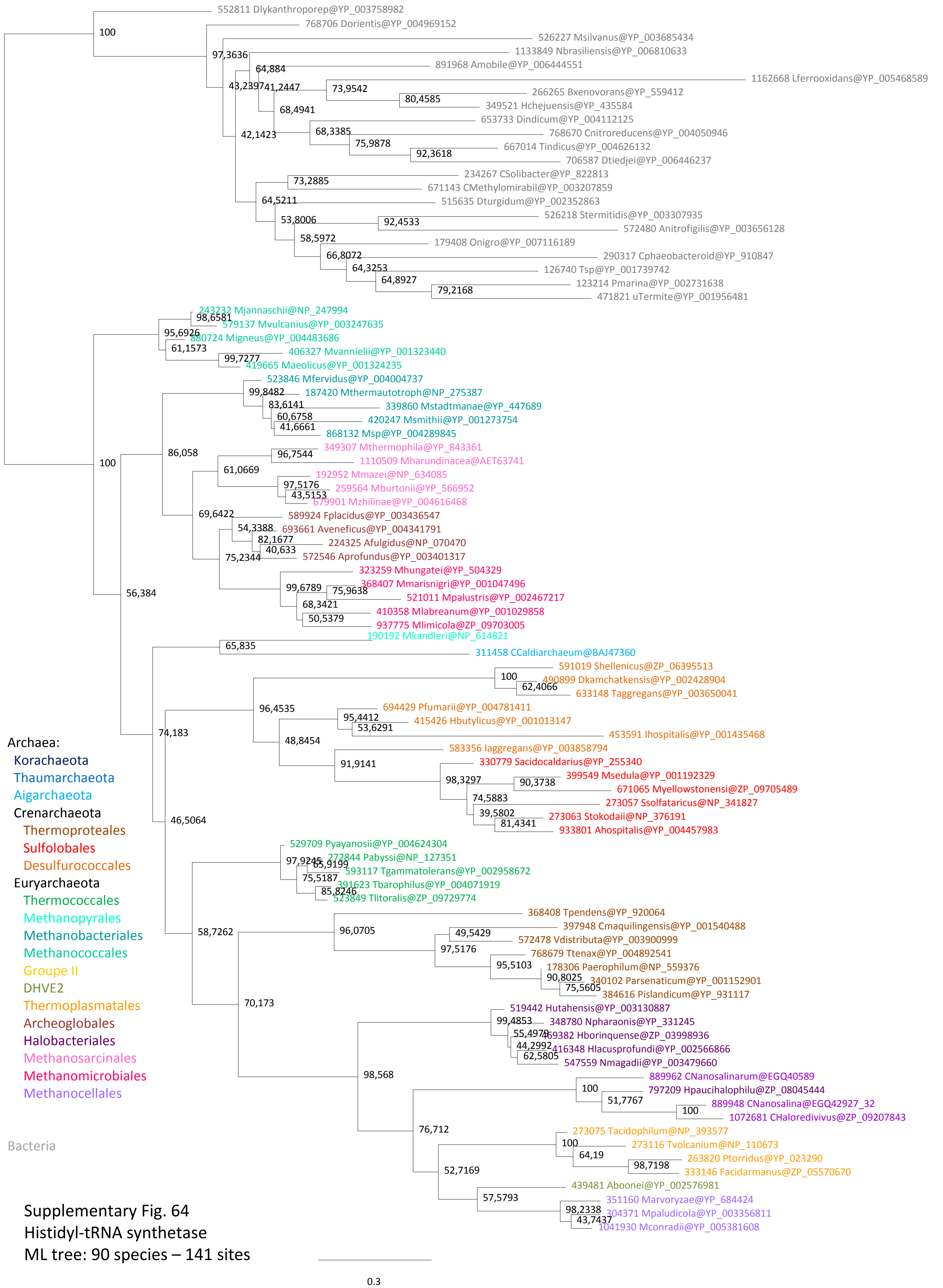


Supplementary Fig. 64
Histidyl-tRNA synthetase
ML tree: 90 species – 141 sites

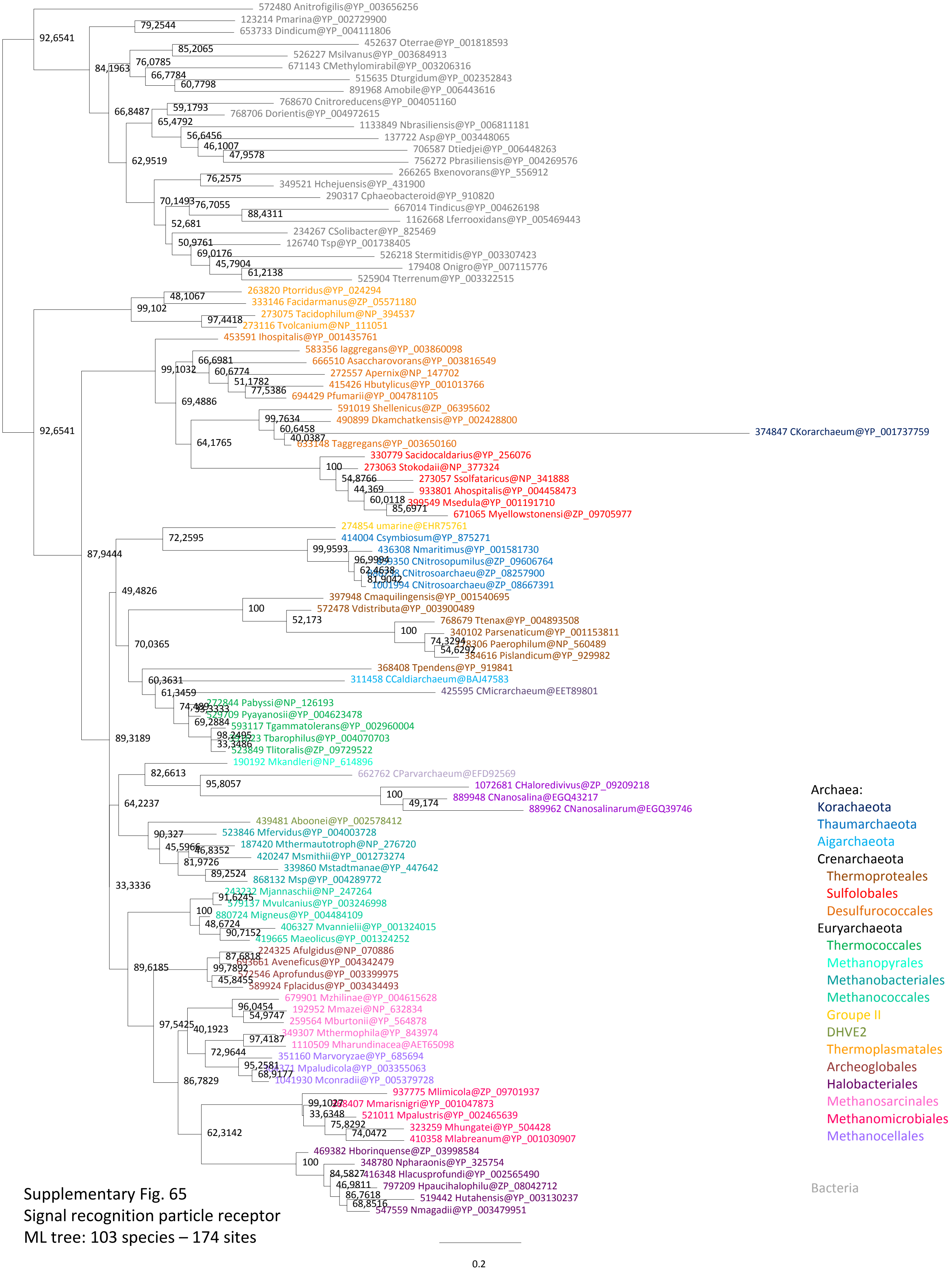


Supplementary Fig. 65
Signal recognition particle receptor
ML tree: 103 species – 174 sites

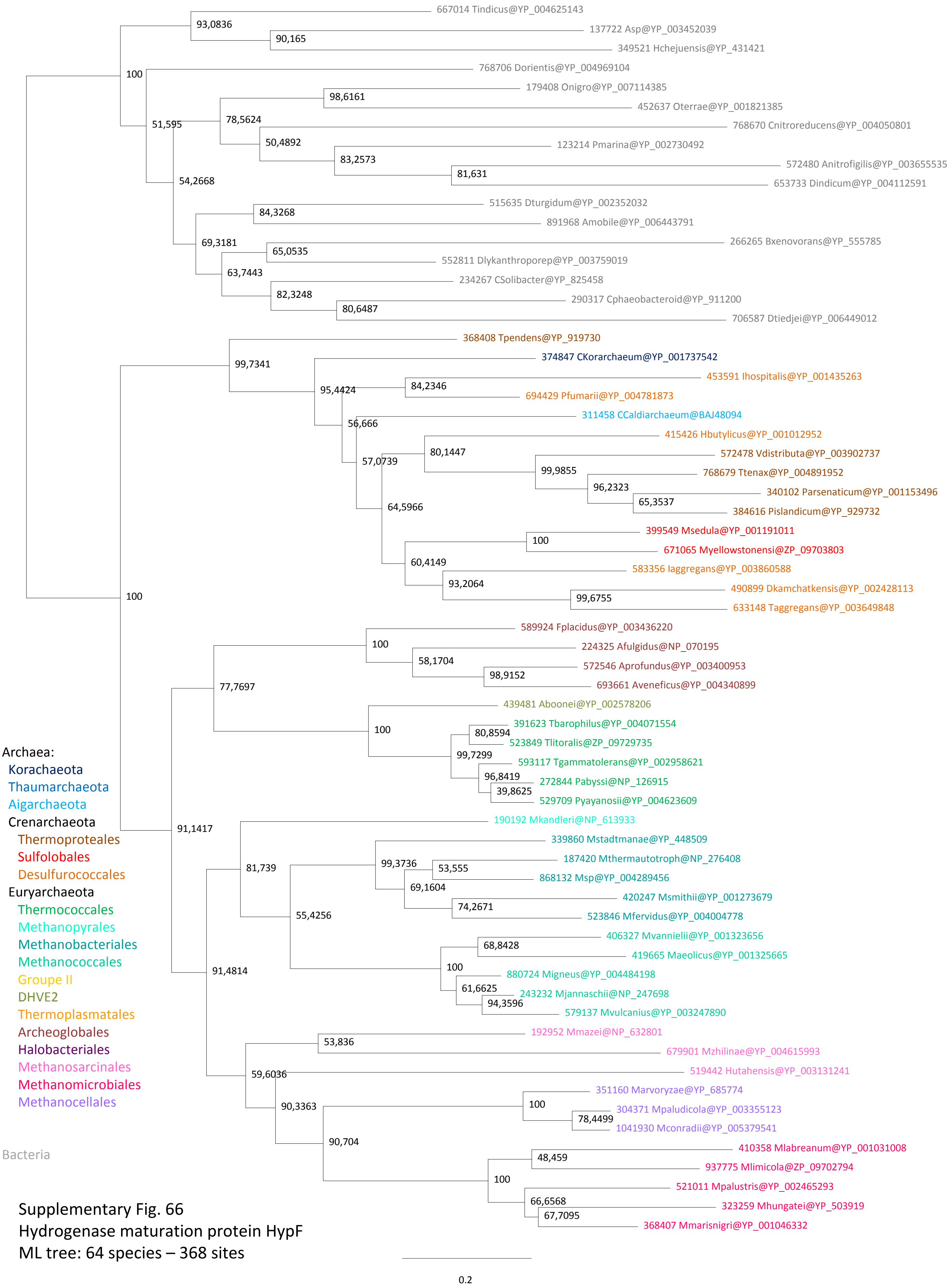


Supplementary Fig. 66
Hydrogenase maturation protein HypF
ML tree: 64 species – 368 sites

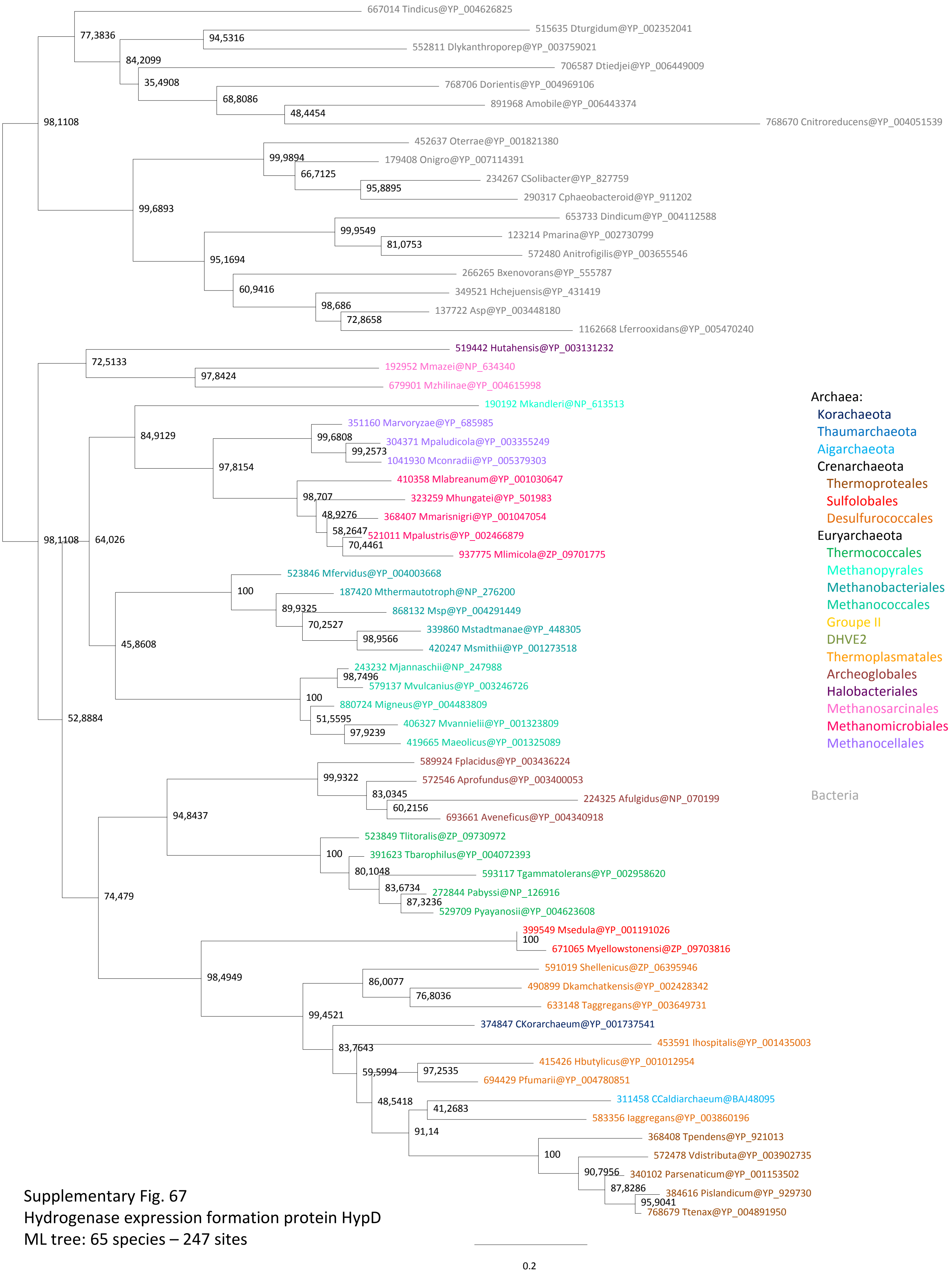


Supplementary Fig. 67
Hydrogenase expression formation protein HypD
ML tree: 65 species – 247 sites

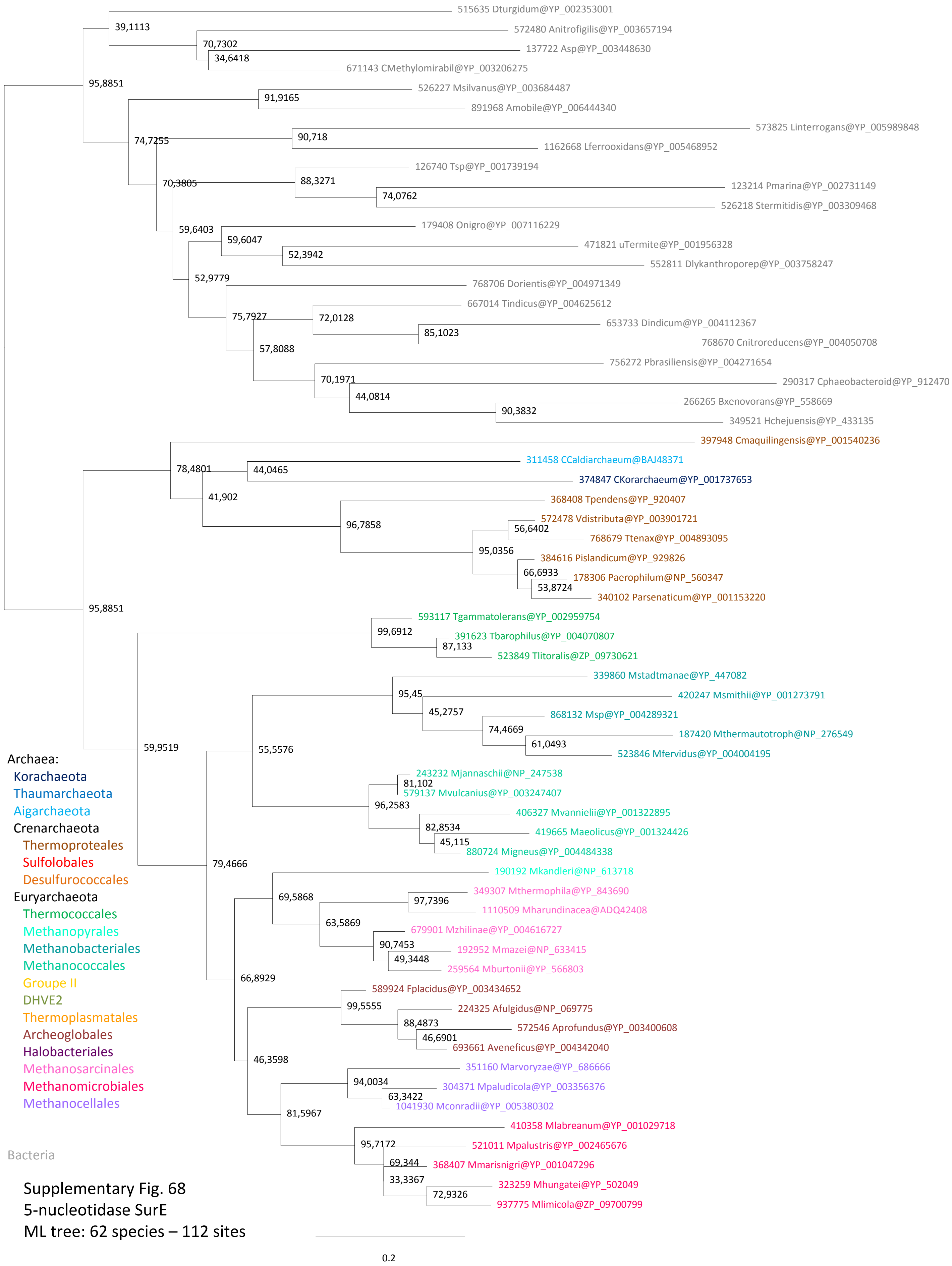


Supplementary Fig. 68
5-nucleotidase SurE
ML tree: 62 species – 112 sites

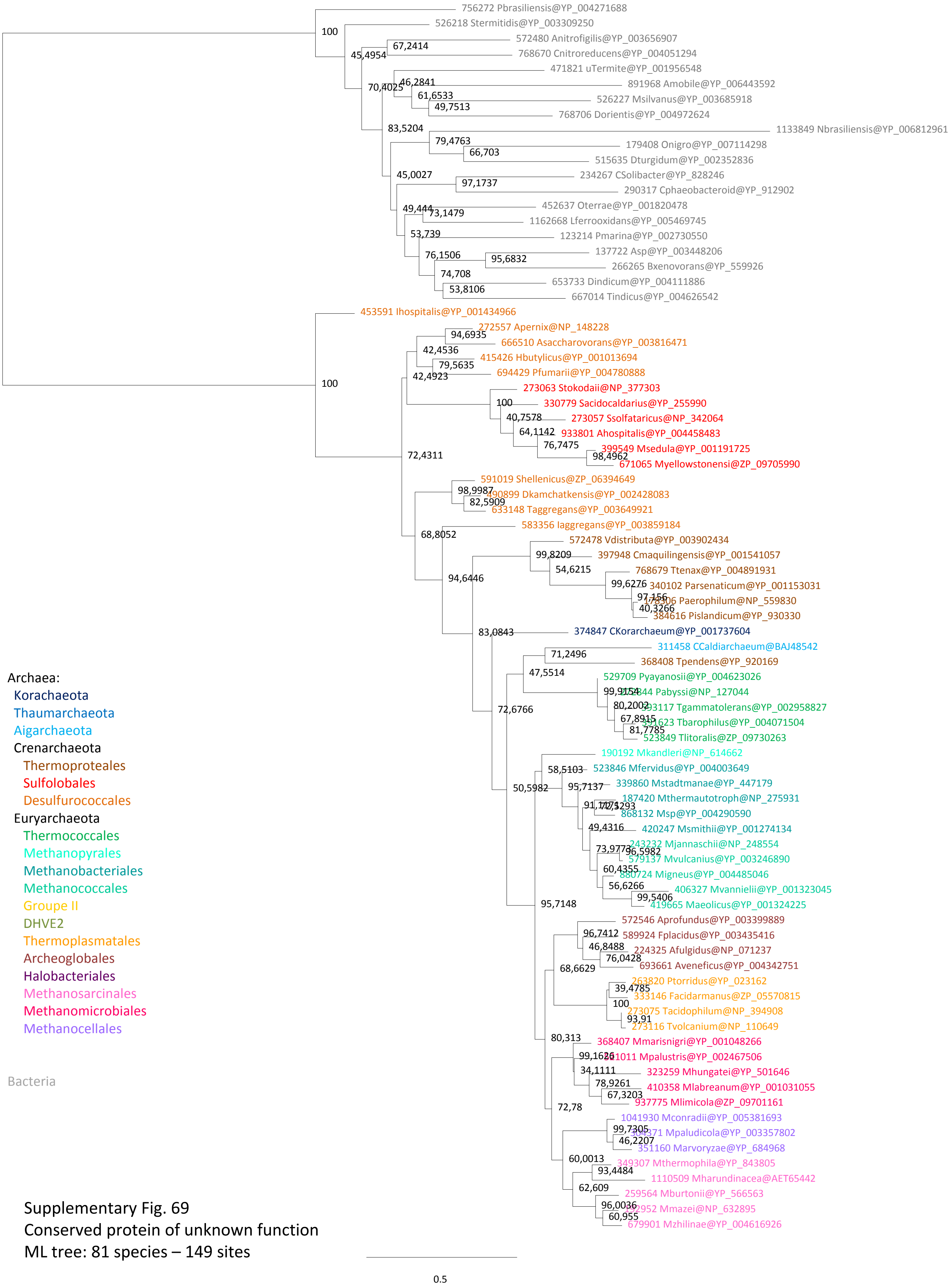


Supplementary Fig. 69
Conserved protein of unknown function
ML tree: 81 species – 149 sites

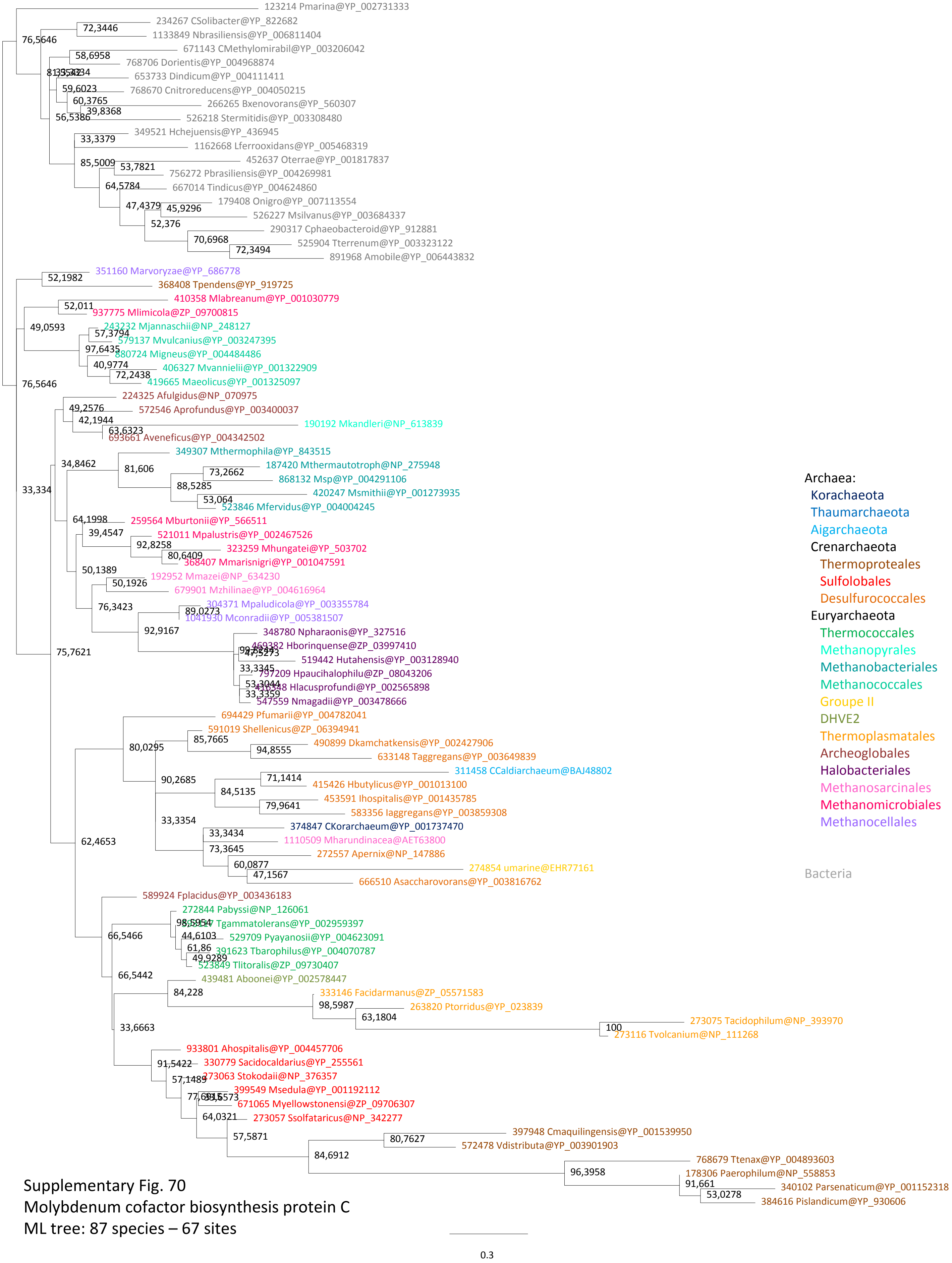


Supplementary Fig. 70
Molybdenum cofactor biosynthesis protein C
ML tree: 87 species – 67 sites

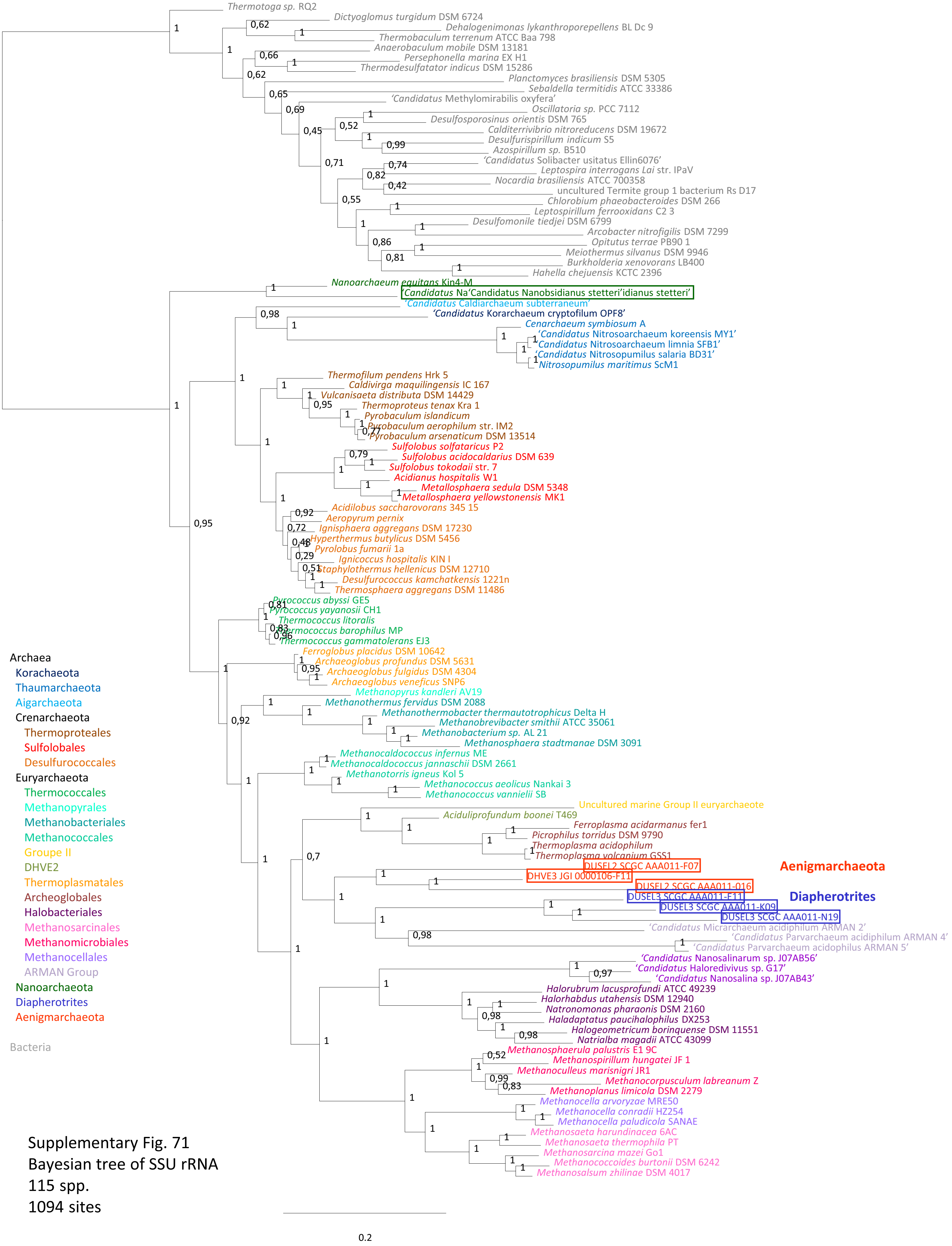


Supplementary Fig. 71
Bayesian tree of SSU rRNA
115 spp.
1094 sites

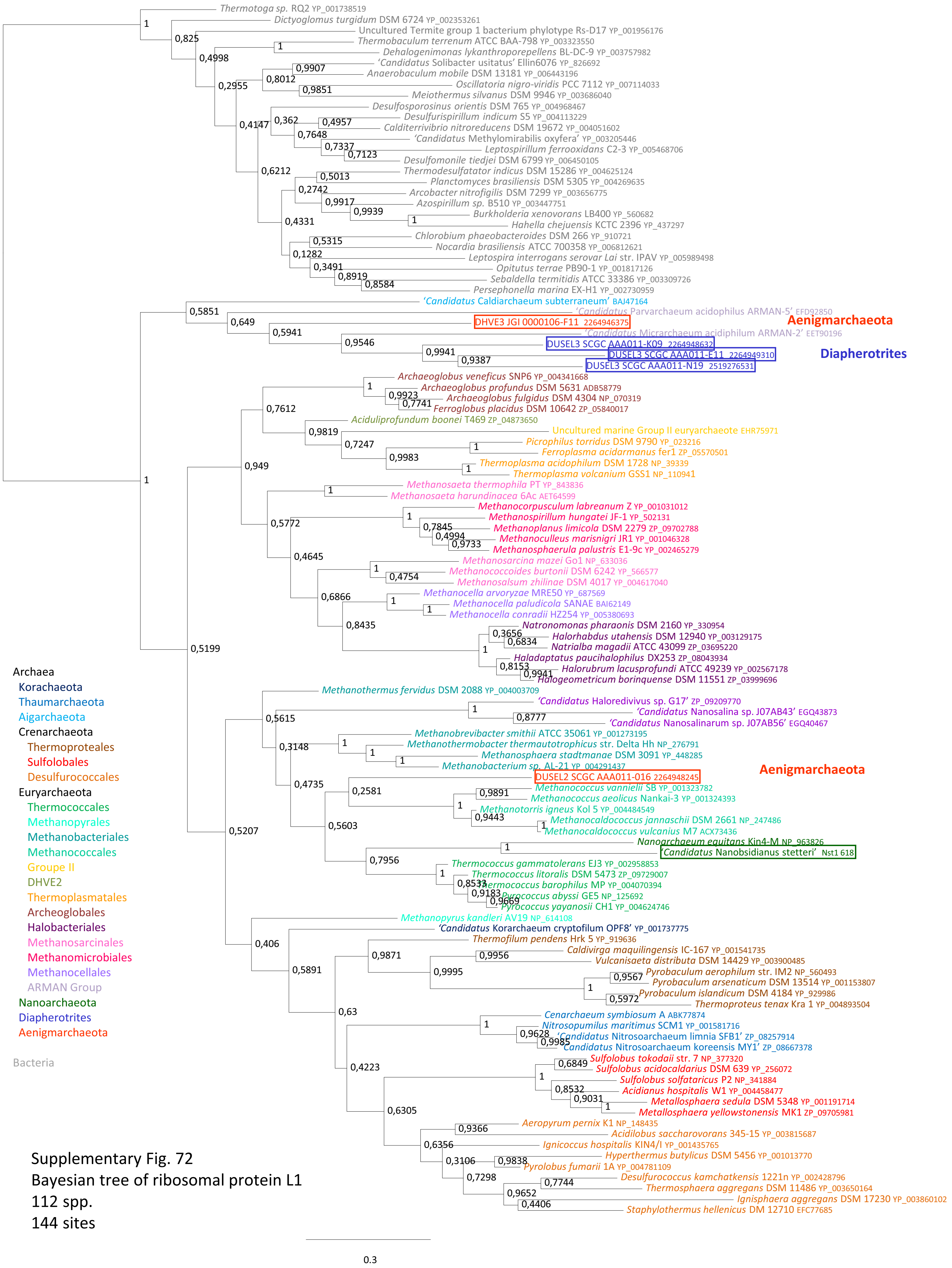


Supplementary Fig. 72
Bayesian tree of ribosomal protein L1
112 spp.
144 sites

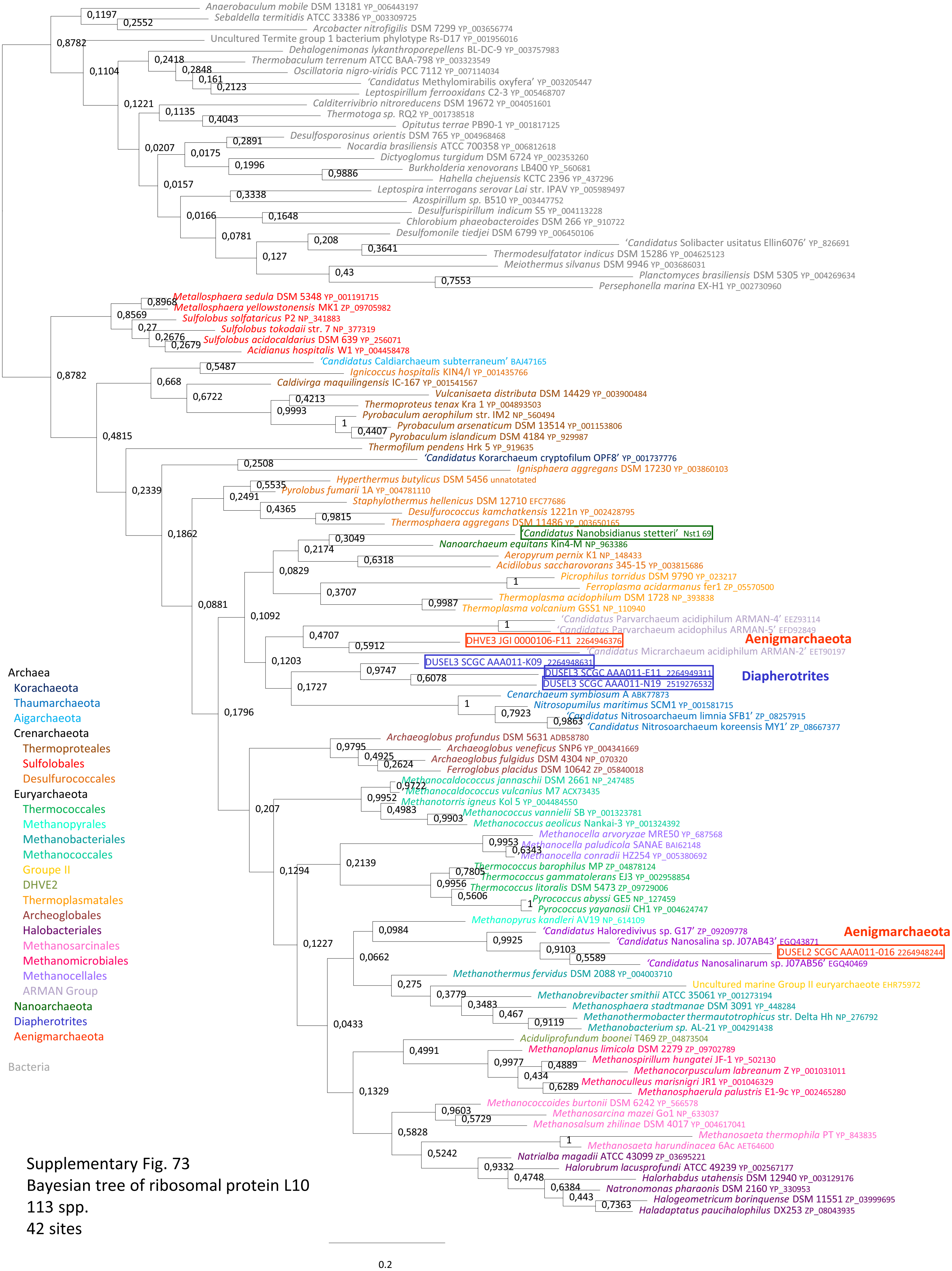


Supplementary Fig. 73
Bayesian tree of ribosomal protein L10
113 spp.
42 sites

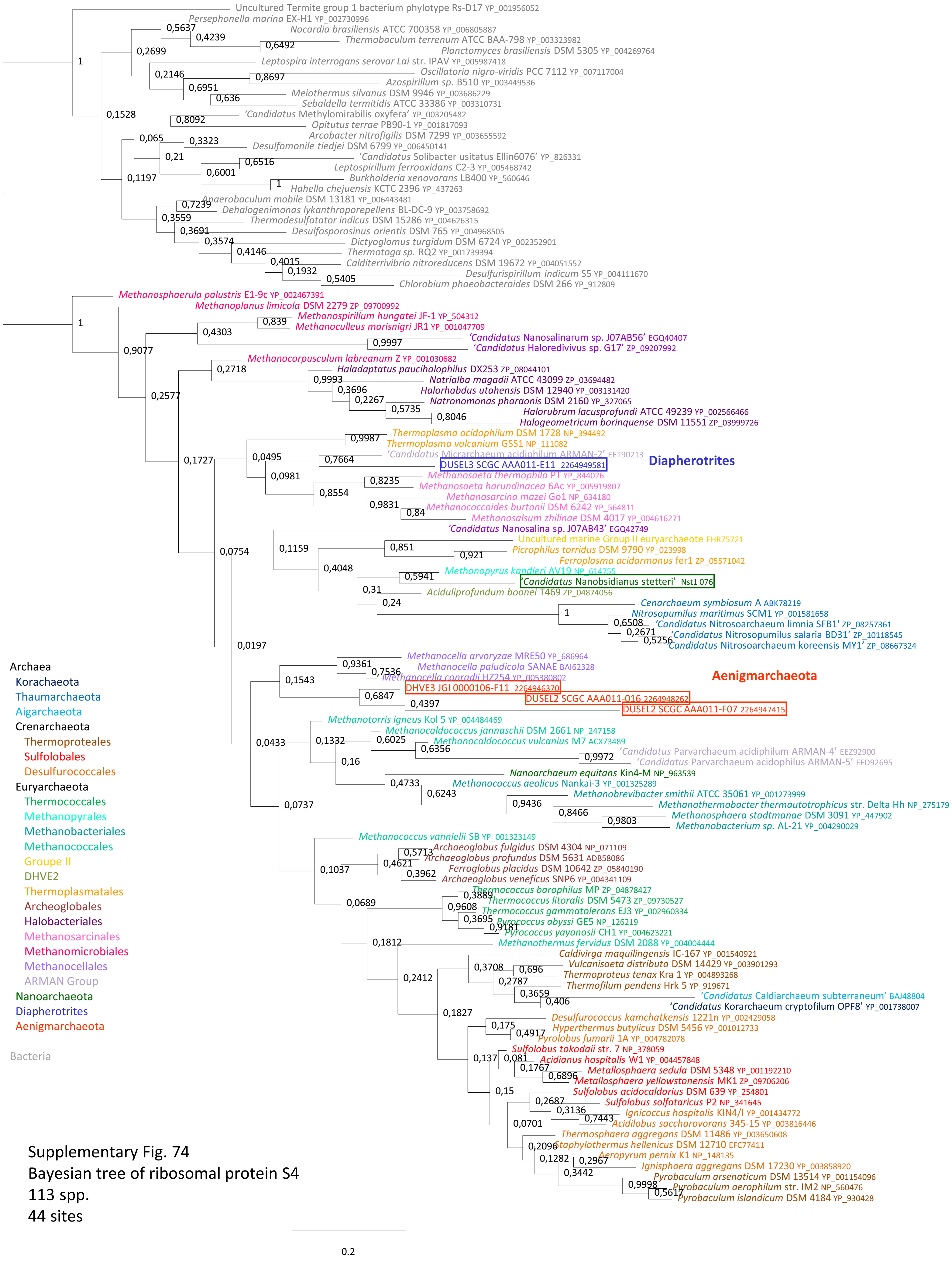


Supplementary Fig. 74
Bayesian tree of ribosomal protein S4
113 spp.
44 sites

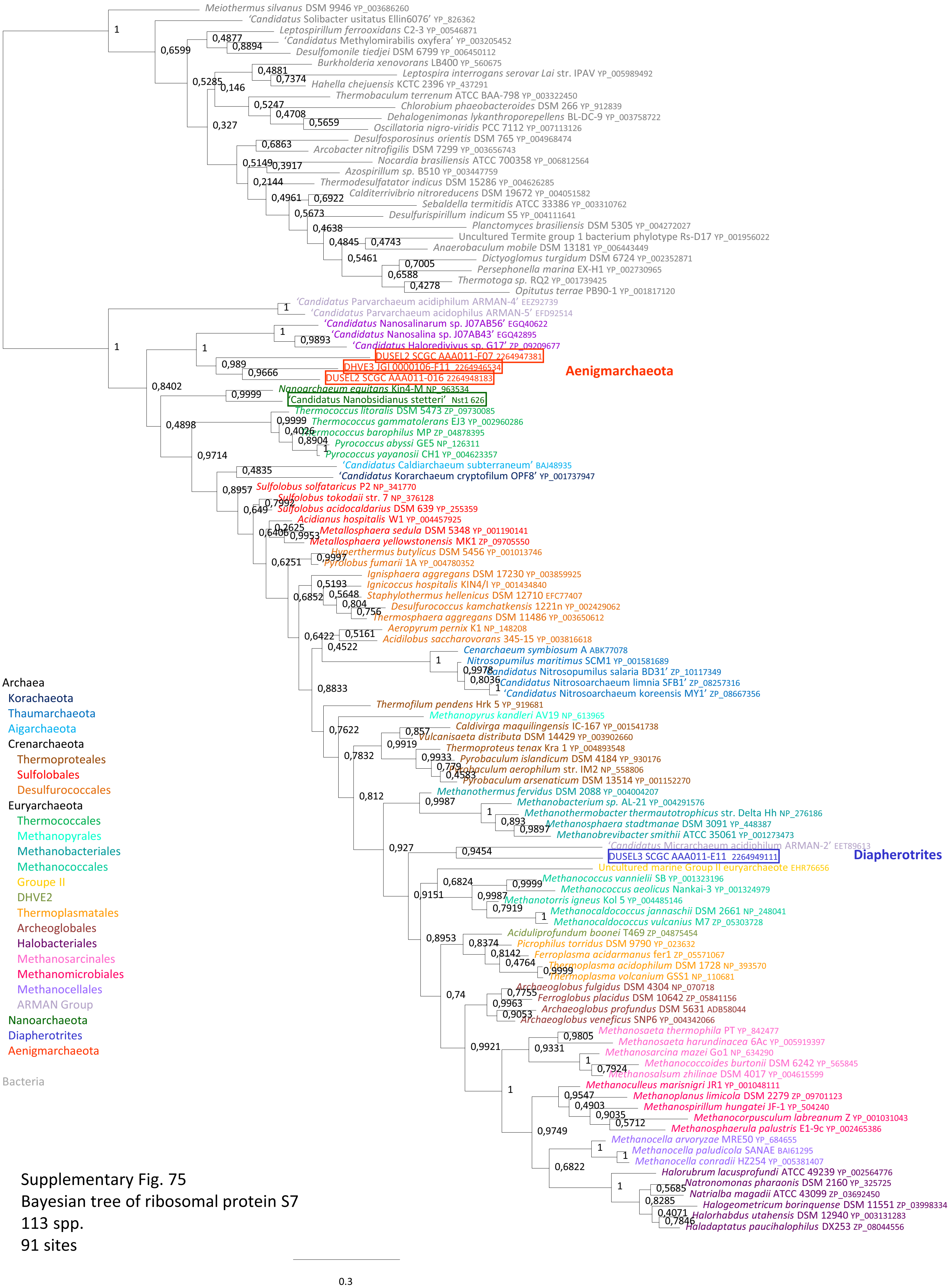


Supplementary Fig. 75
Bayesian tree of ribosomal protein S7
113 spp.
91 sites

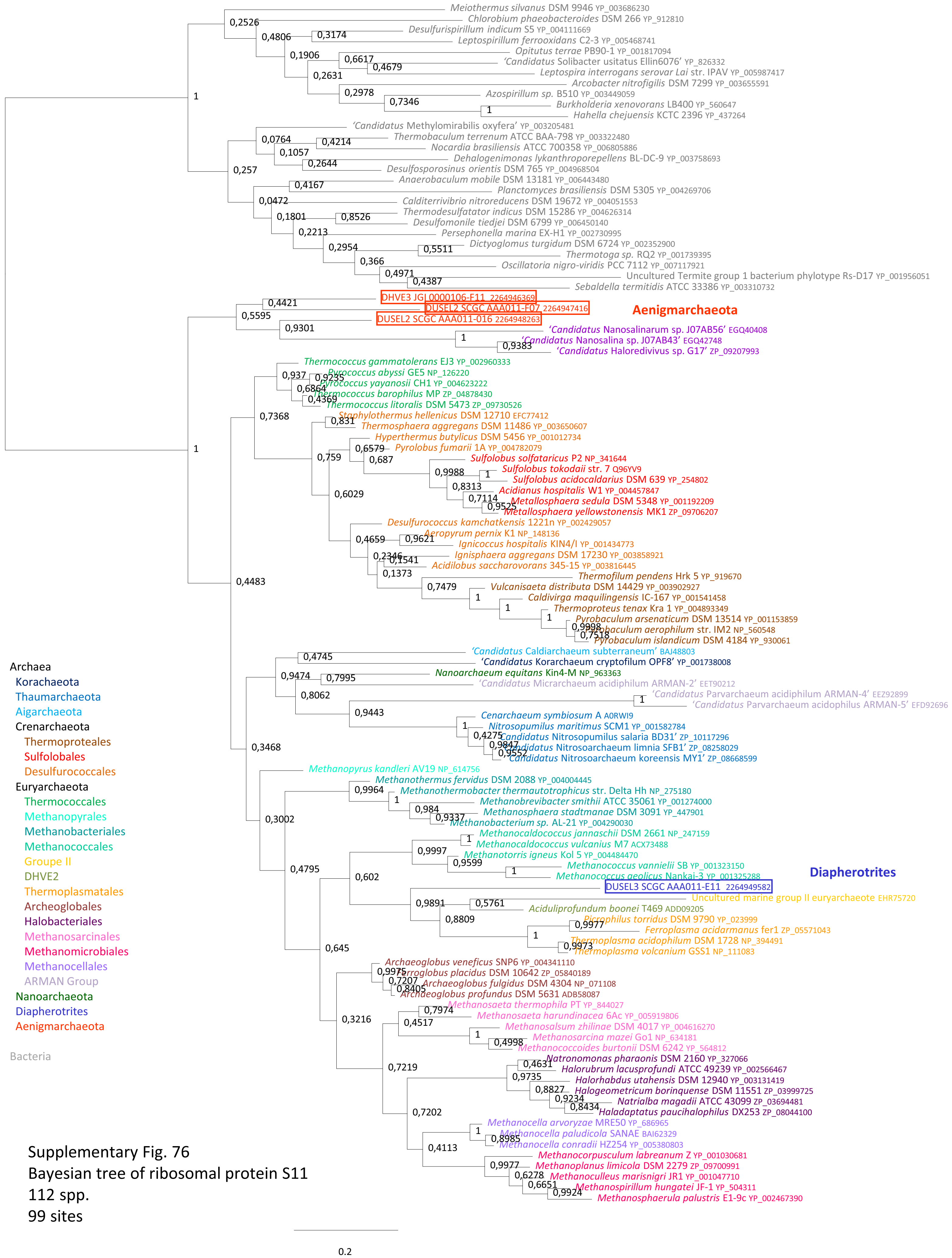


Supplementary Fig. 76
Bayesian tree of ribosomal protein S11
112 spp.
99 sites

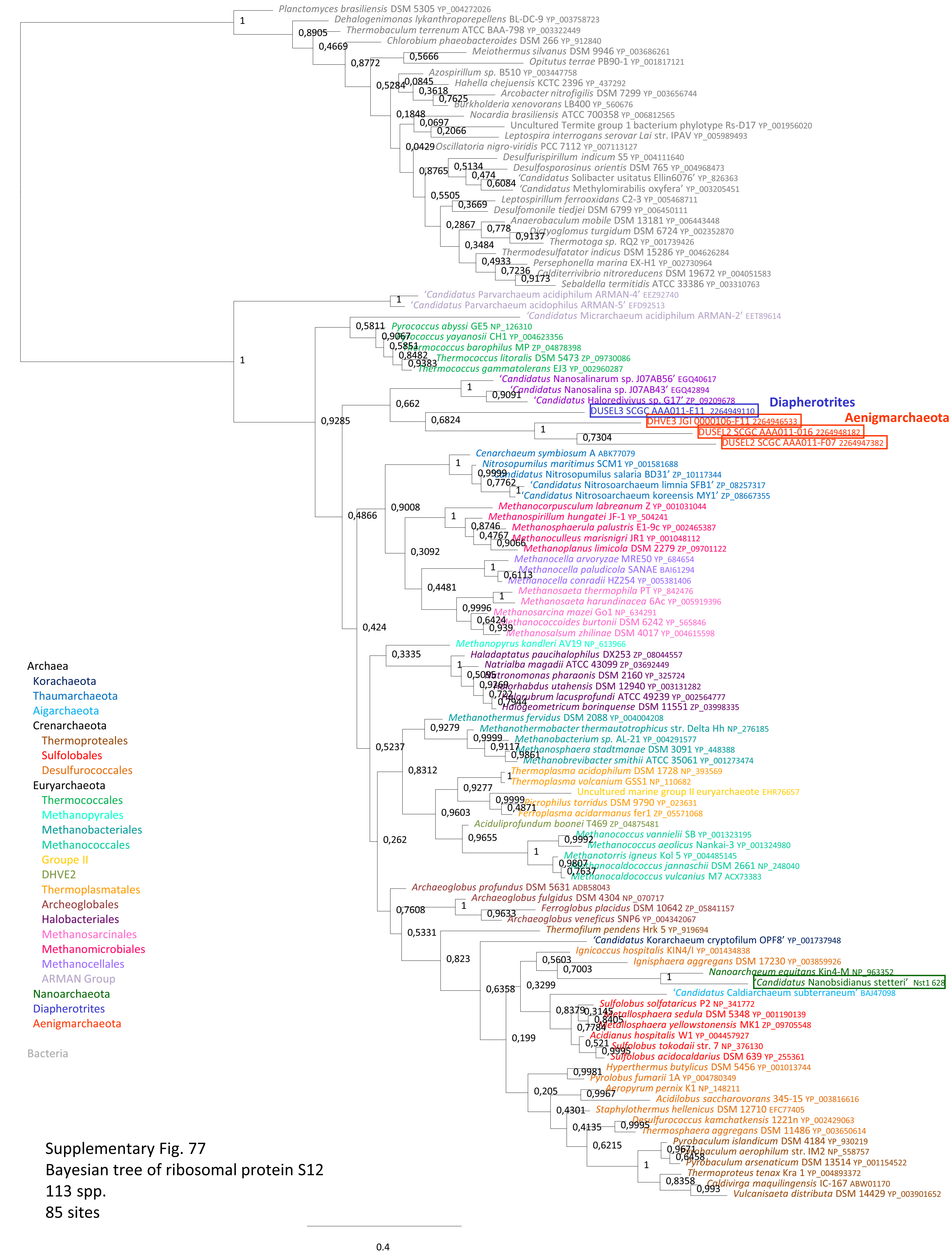


Supplementary Fig. 77
Bayesian tree of ribosomal protein S12
113 spp.
85 sites

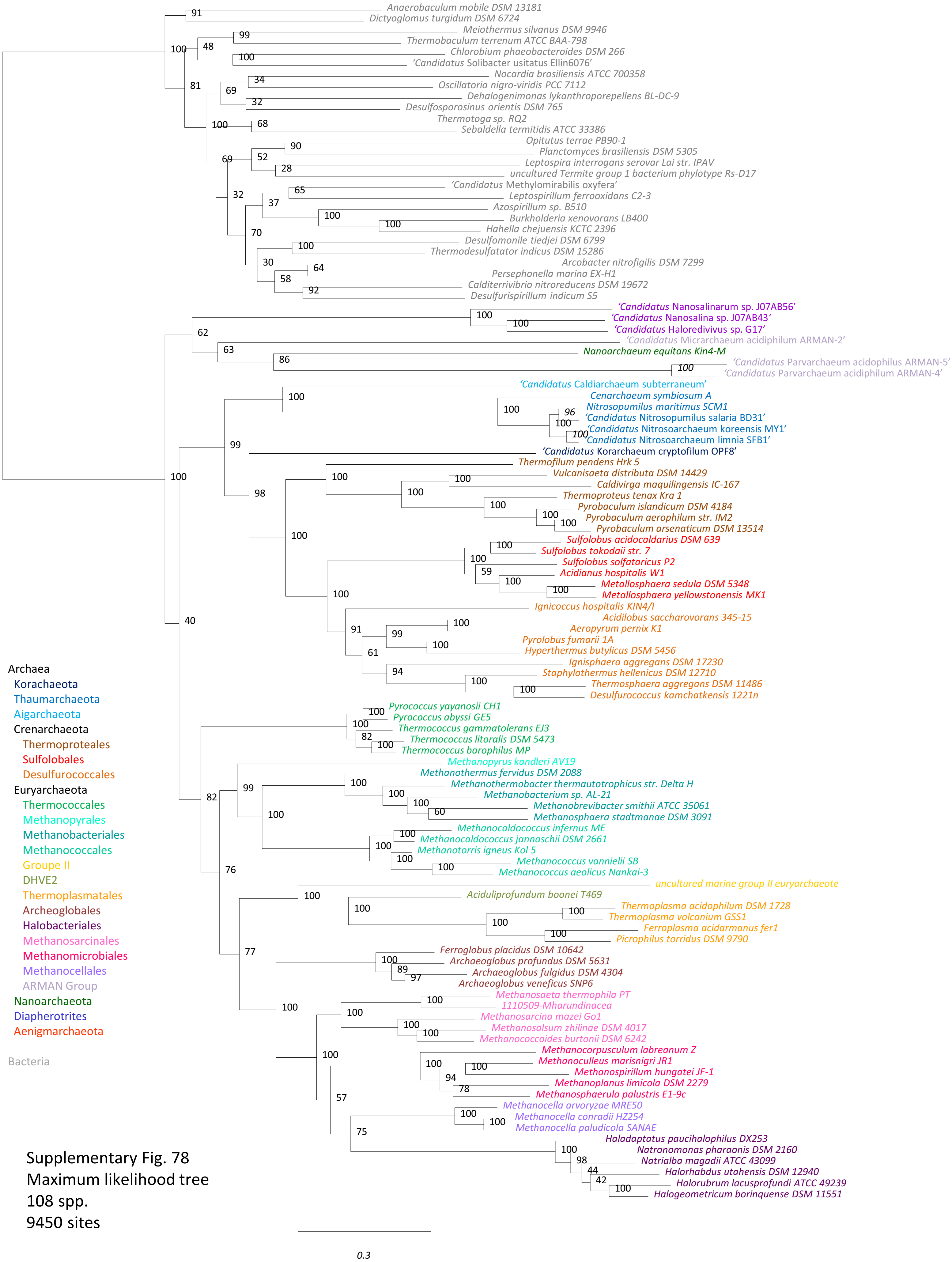


Supplementary Fig. 78
Maximum likelihood tree
108 spp.
9450 sites

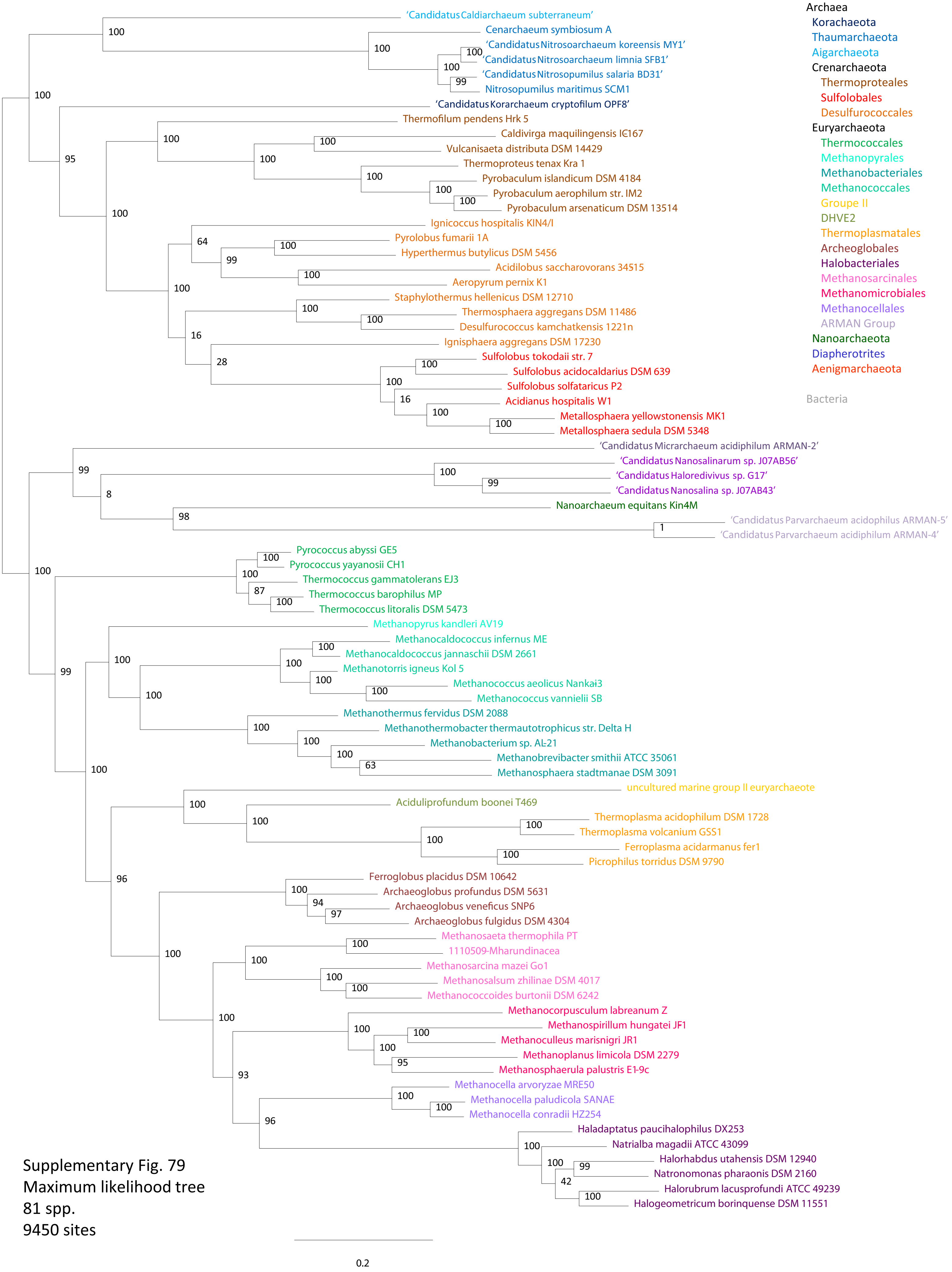


Supplementary Fig. 79
Maximum likelihood tree
81 spp.
9450 sites
